# Transcriptomic analysis of human brains with Alzheimer’s disease reveals the altered expression of synaptic genes linked to cognitive deficits

**DOI:** 10.1093/braincomms/fcab123

**Published:** 2021-06-03

**Authors:** Jamal B Williams, Qing Cao, Zhen Yan

**Affiliations:** Department of Physiology and Biophysics, State University of New York at Buffalo, Jacobs School of Medicine and Biomedical Sciences, Buffalo, NY 14203, USA; Department of Physiology and Biophysics, State University of New York at Buffalo, Jacobs School of Medicine and Biomedical Sciences, Buffalo, NY 14203, USA; Department of Physiology and Biophysics, State University of New York at Buffalo, Jacobs School of Medicine and Biomedical Sciences, Buffalo, NY 14203, USA

**Keywords:** Alzheimer’s disease, transcriptomic analyses, synaptic genes, SNARE complex, cognitive deficits

## Abstract

Alzheimer’s disease is a progressive neurodegenerative disorder associated with memory loss and impaired executive function. The molecular underpinnings causing cognitive deficits in Alzheimer’s disease are loosely understood. Here, we performed cross-study large-scale transcriptomic analyses of postmortem prefrontal cortex derived from Alzheimer’s disease patients to reveal the role of aberrant gene expression in this disease. We identified that one of the most prominent changes in prefrontal cortex of Alzheimer’s disease humans was the downregulation of genes in excitatory and inhibitory neurons that are associated with synaptic functions, particularly the SNARE-binding complex, which is essential for vesicle docking and neurotransmitter release. Comparing genomic data of Alzheimer’s disease with proteomic data of cognitive trajectory, we found that many of the lost synaptic genes in Alzheimer’s disease encode hub proteins whose increased abundance is required for cognitive stability. This study has revealed potential molecular targets for therapeutic intervention of cognitive decline associated with Alzheimer’s disease.

## Teaser

Williams and colleagues have performed large-scale transcriptomic analyses of Alzheimer’s disease patients and revealed the downregulation of genes in cortical neurons that are associated with synaptic functions, particularly the SNARE-binding complex, as the key molecular determinant for cognitive deficits in Alzheimer’s disease.

## Graphical Abstract

**Graphical Abstract:**
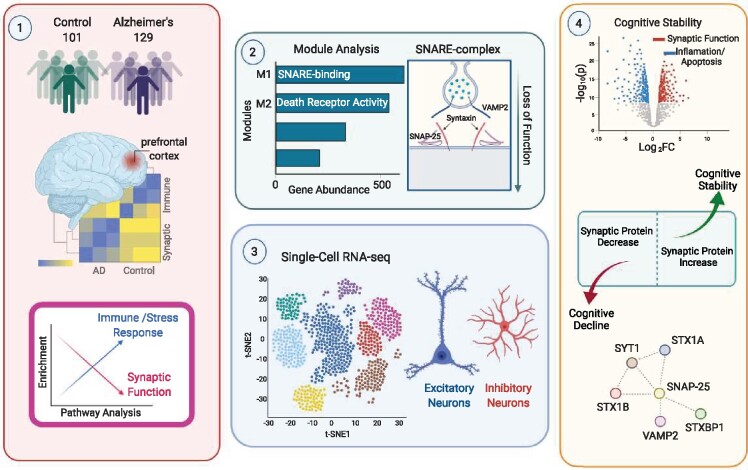

## Introduction

Alzheimer’s disease, the most prevalent neurodegenerative disorder, is marked by the progressive decline of memory and cognitive function. Alzheimer’s disease brains exhibit multiple pathological features, including amyloid plaques, neurofibrillary tangles, astrogliosis, microglia activation and the losses of neurons, neuropil and synaptic elements. However, the causative factors for cognitive decline in Alzheimer’s disease are still unclear. Genomic studies of Alzheimer’s disease human samples have revealed the transcriptional changes of a large number of genes involved in a wide range of pathways, including inflammation, apoptosis and synaptic function.^1–3^ The transcriptomic alteration is also accompanied by aberrant epigenetic modifications in Alzheimer’s disease.^3–8^ Addionally, the advancement of genomic techniques has enabled the detection of single-cell transcriptional changes.^9–14^ Yet, while numerous changes in genes and pathways are reported in Alzheimer’s disease, how the seemingly divergent findings coalesce have yet to be elucidated.

A big challenge for Alzheimer’s disease treatment is the identification of key molecules that cause cognitive impairment at the early stage before global neurodegeneration. Single-cell RNAseq of prefrontal cortex (PFC) of humans with varying degrees of Alzheimer’s disease pathology show that nearly all perturbed genes (>80% downregulated) in the ‘early-pathology’ group occur only in excitatory and inhibitory neurons, while most of the altered genes in the ‘late-pathology’ group are upregulated across cell types and primarily involved in global stress response.^13^ An unbiased proteome-wide association study of cognitive trajectory has found that cognitive stability (CS) is positively correlated with the increased abundance of proteins involving synaptic functions regardless of neurodegenerative pathologies.^15^ It prompts us to contemplate that the loss of synaptic genes important for neuronal plasticity in early-stage Alzheimer’s disease is directly responsible for synaptic dysfunction and cognitive decline.

In this study, we performed in-depth bioinformatic analyses of bulk and single-cell transcriptomic data from human Alzheimer’s disease brains, and revealed the loss of presynaptic and postsynaptic genes involved in vesicle release and synaptic transmission as the most prominent changes in PFC of Alzheimer’s disease humans. Particularly, we identified a systemic loss of genes associated with the SNARE-binding complex, which is essential for vesicle docking and neurotransmitter release.^16–18^ SNARE complex-related genes were also among the top-ranking downregulated genes in excitatory and inhibitory neurons from Alzheimer’s disease patients. Interestingly, we found that many of the lost genes in Alzheimer’s disease encode hub proteins whose increased abundance is required for CS in normal ageing.^15^ These genes provide promising targets for the intervention of cognitive trajectory in Alzheimer’s disease.

## Materials and methods

### Genomic data processing and differential expression analysis

The 230 human dorsolateral PFC samples were separated based on a final clinical diagnosis of late-onset Alzheimer’s disease (129 samples) or being healthy (101 samples) followed by postmortem pathological confirmation. The RNA microarray dataset GSE44770 ^1^ was acquired using NCBI’s public database GEO, followed by processing and analysis in Phantasus (https://artyomovlab.wustl.edu/phantasus/ Accessed 14 June 2021). Differential expression of genes between Alzheimer’s disease and control groups were obtained using the Limma analysis package. Genes with differential expression of adjusted *P*-values below 0.05 and |fold change (FC)| above 1.1 were considered as being significantly different.

### GO enrichment analysis

Enrichment analysis of differentially expressed genes (DEGs) from the RNA microarray dataset was performed using Metascape (http://metascape.org Accessed 14 June 2021), as previously reported.^19^ In addition, the Metascape multi-gene-list meta-analysis tool was used to compare common functional pathways among DEGs from bulk tissues or single excitatory or inhibitory neurons. The three gene lists were analysed for GO biological process enrichment individually and then consolidated into one list and enrichment analysis was performed again. A more detailed explanation for using Metascape to perform GO pathways analysis on multiple gene lists can be found at: https://metascape.org/gp/index.html#/menu/manual_meta Accessed 14 June 2021. In addition to enrichment bar graphs, Circos plots were also used to display the individual connections of genes and pathways between groups. Additionally, Enrichr (https://maayanlab.cloud/Enrichr/ Accessed 14 June 2021) was used to characterize molecular function changes in modular co-expression analysis. To investigate dysregulated synaptic organization in Alzheimer’s disease, we submitted the downregulated RNA microarray dataset to the synaptic ontology database SynGO (https://www.syngoportal.org/ Accessed 14 June 2021). The *brain expressed* background gene set was used to identify the enriched synaptic components in Alzheimer’s disease DEGs.

### Hub genes and network interactome

Gene sets in which hub analysis was performed were first uploaded to Cytoscape, an open-source network visualization software.^20^ An interactome of these genes were then generated using the STRING platform, a database for protein-protein interactions.^21^ Then, using the Cytoscape plugin *cytoHubba*, hub genes for each input dataset were identified and ranked.^22^ The Maximal Clique Centrality (MCC) algorithm was used to rank top-defined nodes (genes) in each dataset. The results were then visualized in Cytoscape.

### Modular co-expression analysis

Modular analysis was performed using webCEMiTool to identify distinct dysregulated co-expression networks that highlight functional changes in Alzheimer’s disease.^23^ In brief, gene expression data from the RNA microarray dataset were uploaded to CEMiTool (https://cemitool.sysbio.tools/analysis Accessed 14 June 2021), and filtered using an unsupervised sorting algorithm. Twelve modules were then generated, each containing genes with similar expression patterns.

### Gene set enrichment analysis

To compare gene expression profiles identified in our RNA microarray dataset (GSE44770) to proteins required for CS, gene set enrichment analysis (GSEA) was performed using GSEA v.4.0.3 (http://www.broadinstitute.org/gsea Accessed 14 June 2021). First, the CS markers identified by proteomic analysis^15^ were separated into two files (higher-abundance in CS and lower-abundance in CS), formatted as *gmt* files and uploaded as two *Gene Set Databases*. Then the microarray genes were ranked and formatted as a *rnk* file and uploaded as a *RankedGeneList*. These pre-ranked genes were then run on the GSEA platform, where each gene was compared to the CS dataset, accumulating a running-sum statistic. This result is represented as an enrichment score (ES), and signifies if a gene set (in our case the CS markers) is positively or negatively correlated with the gene list (RNA-microarray gene set). However, the standard value to consider is normalized enrichment score (NES), which accounts for variability in gene set sizes.

To interpret the GSEA enrichment plots, emphasis should be directed to the peak of the green curve, which represents the ES. The peak will be above 0 for positive ES values, and below 0 for negative ES values. The blue shaded regions to the left or right of the peak are called the *leading edge subset* and represent genes (vertical black lines) within the dataset that contribute to this ES.

### Quantitative real-time PCR

Postmortem human frontal cortex (Brodmann’s Area 10) from Alzheimer’s disease patients and control subjects were provided by NIH NeuroBioBank. Upon arrival, tissue was stored in a −80°C freezer until used for RNA and protein extraction. RNA was extracted from human postmortem tissue by using TRIzol RNA Isolation Reagents (Invitrogen). RNA concentration was measured with Nanodrop and equal amounts of RNA (1 µg) were reversed transcribed using iScript reverse transcription kit (Bio-Rad). Quantitative real-time PCR was performed with SYBR-Green-based reagents, detected by the iCycler iQ™ Real-Time PCR Detection System and iQ™ Supermix (Bio-Rad) according to the manufacturer’s instructions. GAPDH was used as the housekeeping gene to normalize the expression of target genes in samples. FCs in the target genes were determined by normalizing raw fluorescent values of Alzheimer’s disease group to control group using the following formula: FC = 2^−^^Δ(Δ^^*C*^_T_^)^, where Δ*C*_T_ = *C*_T(target)_ – *C*_T(GAPDH)_, and Δ(Δ*C*_T_) = Δ*C*_T(Alzheimer’s disease)_ − Δ*C*_T(control)_. *C*_T_ (threshold cycle) is defined as the fractional cycle number at which the fluorescence reaches 10× the standard deviation of the baseline. A total reaction mixture of 18 µl was amplified in a 96-well thin-wall PCR plate (Bio-Rad) using the following PCR cycling parameters: 95°C for 5 min followed by 40 cycles of 95°C for 30 s, 55°C for 30 s, and 72°C for 60 s. Primers sequences used for PCR were as follows:

STX1A (F: CGTGGAGAGCCAGACTATGT; R: CTGGAGTGGAGTGGCAGTTT), SNAP25 (F: TCCCGAGAAGCCCAGGTAAG; R: GCAGCTCACCTCGAAAACAC), VAMP2 (F: GTCTCTCCTGCGTTCCCC; R: CGACCTCACAGATGCGATCC), SNAP29 (F: CTGGCCCTCATGTACGAGTC; R: AGGGTGCCATTCTGTTCAGG), VAMP5 (F: AATAGAGTTGGAGCGGTGCC; R: AGGAGTTGGTCTGAACGCTG), STX2 (F: AAAGGCCGCATCCAGCG; R: TGCTGGTCTCCAGCTTCAT), SYP (F: GTCAGTTCCGGGTGGTCAAG; R: AAGTACACTTGGTGCAGCCT), SYT1 (F: GTGGTGGTAACTGTTTTGGACT; R: ATGTCTGACCAGTGTCGCAG).

### Western blotting of synaptic proteins

Synaptic protein isolation was performed as follows. Briefly, the human postmortem PFC tissue was lysed and homogenized in ice-cold lysis buffer (10 ml/g, 15 mM Tris, pH 7.6, 0.25 M sucrose, 1 mM EGTA, 2 mM EDTA, 25 mM NaF, 10 mM Na_4_P_2_O_7_, 10 mM Na_3_VO_4_, 1 mM PMSF, and protease inhibitor tablet). After centrifugation at 800 × *g* for 5 min to remove nuclei and large debris, the remaining supernatant was subjected to 10,000 × *g* centrifugation for 10 min. The crude synaptosome fraction (pellet) was suspended in lysis buffer containing 1% Triton X-100 and 300 mM NaCl, homogenized again, and centrifuged at 16,000 × *g* for 15 min. Triton insoluble fraction, which mainly includes membrane-associated proteins from synpatosomes, was dissolved in 1% SDS. Protein concentration was measured by the BCA assay (Thermo Fisher). Samples were boiled in 2× SDS loading buffer for 5 min, and separated on 7.5% SDS-PAGE. Western blotting of synaptic proteins was performed by incubating overnight with the following primary antibodies: STX1A (1:500, Proteintech, 66437-1-Ig), SNAP25 (1:500, Proteintech, 60159-1-Ig), VAMP2 (1:500, Proteintech, 10135-1-AP), PSD95 (1:500, Cell Signaling Technology, 2507) and ACTIN (1:1000, Santa Cruz, sc-47778). After the incubation with a secondary antibody (horseradish peroxidase-conjugated), ECL reaction was performed using enhanced chemiluminescence substrate (Thermo Scientific). Luminescence was detected by Chemidoc XRS system (Bio-Rad) and density of blots was quantified by ImageJ software (NIH).

### Immunohistochemistry

Human postmortem PFC tissue was cut into a small chunk (1 cm^3^), followed by fixing in 4% paraformaldehyde at 4°C overnight before cutting into 50 µm slices. Sections were washed in PBS (3 times, 15 min each) and blocked in 5% goat serum (1 h, RT), then stained overnight with STX1A (1:50, Proteintech, 66437–1-Ig) and NeuN (1:500, Novus Biologicals, NBP1-77686), or SNAP25 (1:50, Proteintech, 60159–1-Ig) and NeuN (1:500, Novus Biologicals, NBP1-77686), or VAMP2 (1:50, Proteintech, 10135–1-AP) and NeuN (1:500, Millipore, MAB377) at 4°C. After washing three times in PBST (1× PBS, 0.05% Tween^®^ 20), slices were incubated with two secondary antibodies (Alexa Fluor 488, 1:1000, Thermo Fisher Scientific, A-11008) and (Alexa Fluor 594, 1:1000, Thermo Fisher Scientific, A-11032) for 1 h at room temperature, followed by three washes with PBST. Slices were mounted on slides with VECTASHIELD mounting media (Vector Laboratories). Images were acquired using a Leica TCS SP8 Confocal Microscope with excitation lasers of 405 nm (DAPI), 488 nm and 594 nm wavelengths. Applying the same settings (e.g. laser power intensity) for each condition, Z-stack images were acquired and analysed by Image J. Consistent cutoff thresholds for each projected image were applied, then analysis was performed by measuring puncta intensity using the RawIntDen function and puncta area using the Total Area function in Image J. All specimens were imaged under identical laser power and analysed with identical parameters.

### Statistical analysis

Data were analysed with GraphPad Prism v.6 (GraphPad). All values are mean ± SEM. Differences between two groups were assessed with unpaired Student’s *t*-test with unequal variance.

### Data availability

The data used to compare gene expression between Alzheimer’s disease and healthy controls were from the RNA microarray public dataset deposited in GEO under the accession number GSE44770 .^1^ The data used to compare gene expression in excitatory and inhibitory neurons were from human snRNA sequencing data deposited in Synapse (https://doi.org/10.7303/syn21125841 Accessed 14 June 2021).^19^ The data of cognitive trajectory proteins were obtained from the large-scale proteomic study.^15^

## Results

### Differential gene expression analysis identifies the loss of synaptic genes in PFC of Alzheimer’s disease patients

Postmortem brain tissue was collected from the dorsolateral PFC of 129 late-onset Alzheimer’s disease patients and 101 non-demented healthy controls from the Harvard Brain Tissue Resource Center. Patients from which these samples were acquired received a clinical diagnosis of Alzheimer’s disease or were otherwise deemed healthy. These postmortem tissues underwent extensive screening for Alzheimer’s disease pathology prior to analysis. Gene expression analyses were then conducted via RNA array hybridization technology, where age, sex, RNA integrity, postmortem interval and sample pH were normalized accordingly, and deposited in NCBI’s gene expression omnibus (GEO) database.^1^

**Figure 1:**
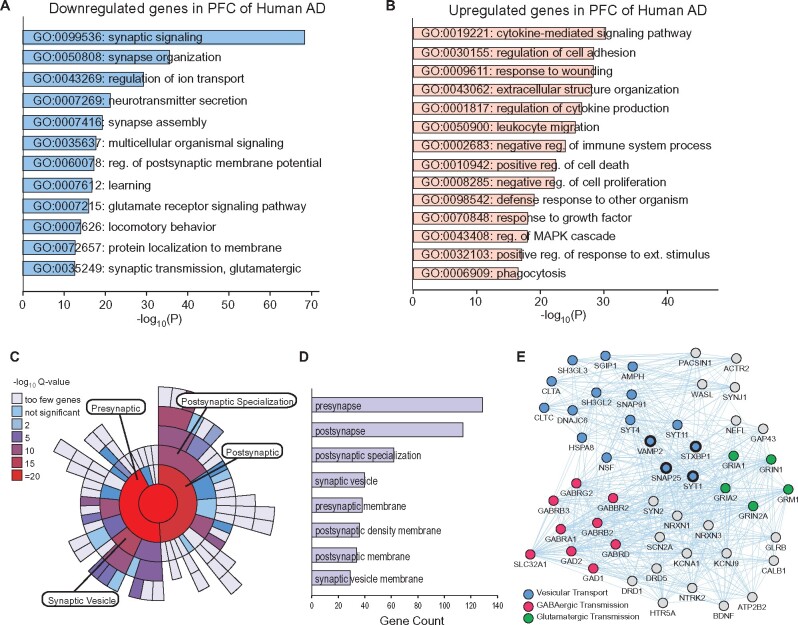
**Differential gene expression analysis of Alzheimer’s disease patients.** (**A, B**) GO analysis of molecular functions associated with downregulated (**A**) or upregulated DEGs (**B**) in PFC of Alzheimer’s disease samples. (**C**) Sunburst plot representing cellular component enrichment analysis of downregulated synaptic genes in PFC of Alzheimer’s disease samples. Higher red intensities are associated with more significant enrichments. All the identified synaptic genes are represented in the red circle at the center of the plot. Presynaptic genes are the most overrepresented synaptic subcluster. (**D**) Synaptic cellular component analysis representing gene abundance and enrichment in each synaptic cluster. (**E**) Interaction networks of top 50 downregulated synaptic hub genes in Alzheimer’s disease samples.

We downloaded this microarray gene expression dataset, then calculated and examined the value distribution for all 230 samples. The data displayed a median-centered distribution, suggesting that the dataset is normalized and cross-comparable. Analyses of the transcriptomic data with a cutoff of adjusted *P* < 0.05 and FC of 10% identified 2174 DEGs (1241 down; 933 up) (Supplementary Table 1). GO enrichment analyses of these significant DEGs indicated that the most prominently downregulated pathways in Alzheimer’s disease are synaptic signalling, synapse organization, ion transport, neurotransmitter secretion and glutamatergic synaptic transmission (Fig. 1A;Supplementary Table 2). On the other hand, the most prominently upregulated pathways in Alzheimer’s disease were those involved in immune response activation via cytokine-mediated pathways, cell adhesion and structural organization, and cell death (Fig. 1B).

With synaptic signalling being the most significantly downregulated GO category, we utilized SynGO, a synaptic gene ontology database, to further explore dysregulated synaptic pathways in Alzheimer’s disease. Among the downregulated DEGs in Alzheimer’s disease, 239 were classified as synaptic genes (Supplementary Table 3). Synaptic enrichment analyses identified presynapse as the most enriched and abundant subcellular component downregulated in Alzheimer’s disease (Fig. 1C and D). Within the presynaptic gene cluster, synaptic vesicle-related genes were the most overrepresented subcategory (Fig. 1C, −Log_10_*P*-value = 14.37).

Hub gene analysis on the 239 downregulated synaptic genes was performed to identify key genes with high connectivity in the gene network. These genes represent the central molecular constituents that are most strongly connected in the downregulated synaptic network, and are presumably most responsible for the network’s global function. We revealed hub genes to be most associated with Synaptic Vesicle Transport (Exocytosis/Endocytosis), Glutamatergic Transmission and GABAergic Transmission (Fig. 1E). Genes essential for the formation of SNARE complex, membrane fusion and exocytotic release, such as *SNAP-25*, *STXBP1* and *VAMP2*,^24–27^ are among the top-ranking nodes. Another top hub gene is *SYT1* encoding the primary calcium sensor synaptotagmin-1, which regulates SNARE zipping and fast presynaptic vesicle exocytosis.^28–30^ Hub gene analyses also revealed the downregulation of synaptic genes encoding postsynaptic receptors, transporters and enzymes mediating glutamatergic transmission (e.g. *GRIA1, GRIA2, GRIN2A* and *SLC17A6*) or GABAergic transmission (e.g. *GAD2, GABRB2* and *SLC6A1*) in Alzheimer’s disease (Fig. 1E). Together, these data indicate that the transcriptional loss of genes controlling synaptic function in PFC, particularly those encoding SNARE complex-associated proteins responsible for neurotransmitter release, is a prominent transcriptomic aberration in human Alzheimer’s disease.

**Figure 2:**
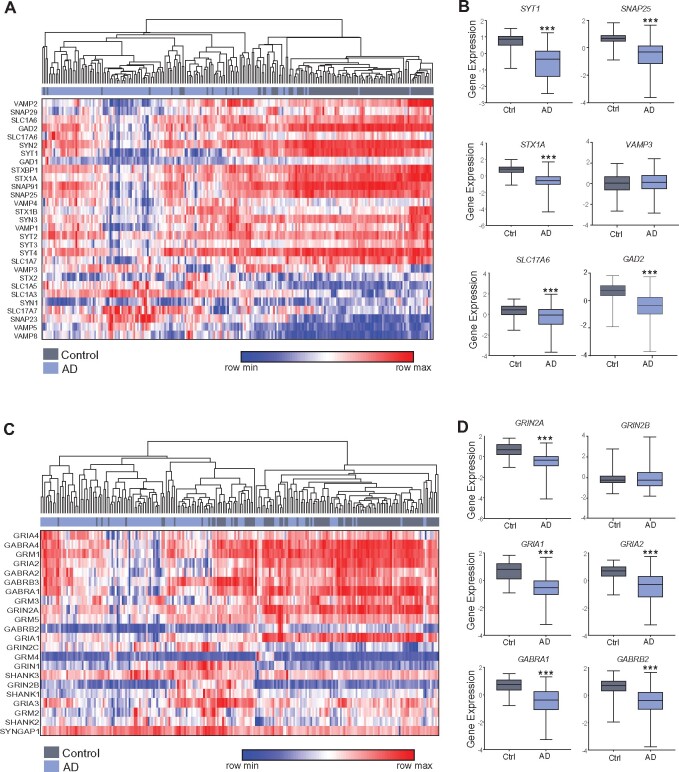
**Transcriptomic data show the loss of synaptic genes in PFC of Alzheimer’s disease patients**. (**A, C**) Heatmaps of synaptic gene expression values (row clustered by k-means) in postmortem PFC from 101 control humans and 129 Alzheimer’s disease patient samples (hierarchal clustered). The genes encode SNARE-complex components; glutamate receptors, transporters, anchoring proteins; GABA receptors, transporters or synthesizing enzymes. (**B, D**) Box plots showing the log_2_ (normalized gene expression) of selected genes (highlighted in blue) in control vs. Alzheimer’s disease humans. ***P < 0.001, *t*-test.

Because of the enrichment of synaptic molecules in downregulated genes associated with Alzheimer’s disease, we further examined presynaptic and postsynaptic genes among the 129 late-onset Alzheimer’s disease patients and 101 non-demented healthy controls. As shown in Fig. 2A and B, many of the SNARE complex genes involved in presynaptic vesicle exocytosis were significantly downregulated in Alzheimer’s disease, including *SNAP-25*, *STX1A*, *SYT1* and *VAMP2*, while *STX2, SYN1* and *VAMP3* were not changed. Some genes encoding postsynaptic glutamate receptors, GABA_A_ receptors or anchoring proteins were also significantly downregulated in Alzheimer’s disease, including *GRIN2A, GRIA1, GRIA2, GABRA1, GABRB2, GRM3* and *SHANK2*, while *GRM5, SYNGAP1, SHANK3* and *GRIN2B* were not changed (Fig. 2C and D). The downregulation of selective synaptic genes in Alzheimer’s disease suggests that these transcriptional changes are not due to the general loss of synapses.

### Gene co-expression network analysis highlights the loss of SNARE complex genes in PFC of Alzheimer’s disease patients

**Figure 3:**
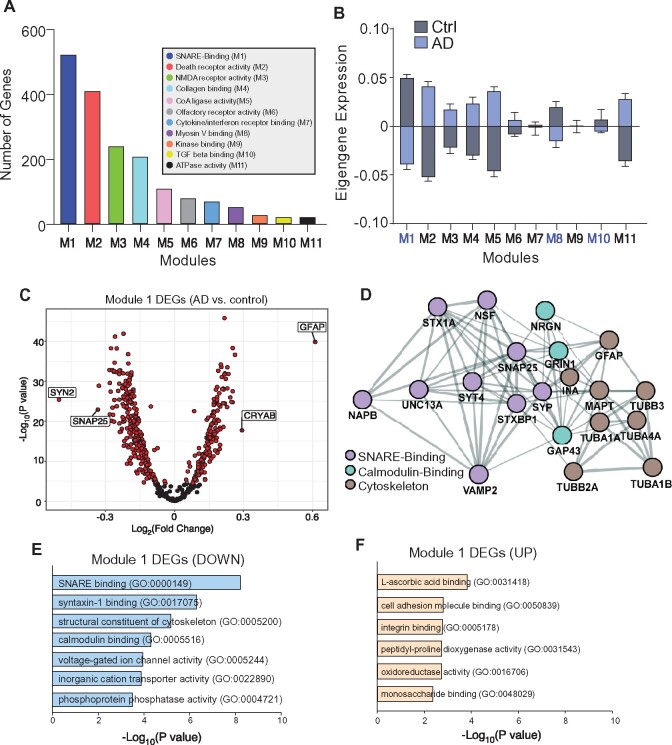
**Modular analysis of Alzheimer’s disease gene expression data.** (**A**) Gene co-expression modules for Alzheimer’s disease DEGs plotted by abundance. (**B**) Plot of mean log_2_ (normalized eigengene expression) values within each module for Alzheimer’s disease and control groups. The bi-directional plot is the mean of a summary score (representative value of the correlation between the sample, module and respective genes) across the entire group, therefore, if the Alzheimer’s disease bar is above zero, then that module is positively correlated with Alzheimer’s disease, and if the Alzheimer’s disease bar is below zero, then that module is negatively correlated with Alzheimer’s disease. (**C**) Volcano plot displaying M1 DEGs. (**D**) Interaction network of M1 DEGs in top GO molecular functional pathways. (**E, F**) GO molecular function pathways enriched in M1 downregulated (**E**) or upregulated (**F**) DEGs.

To classify gene networks that demonstrate functional enrichment in Alzheimer’s disease, we used modular co-expression analysis, which allows us to create weighted gene networks that unbiasedly classify genes whose expression is highly correlated to one another. Using the webCEMiTool algorithm, our Alzheimer’s disease DEGs were classified into 12 distinct modules (Table 1; Supplementary Table 4), with module 1 (M1) being the most abundant, which consisted of 522 DEGs (Fig. 3A). Additionally, Module 1 (SNARE-binding) shows the greatest negative difference in mean eigengene expression between Alzheimer’s disease and control samples, while Module 2 (Death receptor activity) shows the greatest positive difference between Alzheimer’s disease and control samples (Fig. 3B;Supplementary Fig. 1). It suggests that Alzheimer’s disease samples have diminished overall Module 1 gene expression and elevated overall Module 2 gene expression.

**Table 1:** Summary of modules containing co-expressed genes that represent dysregulated molecular functions in PFC of AD patients

Module	Number of genes	Molecular function	Key constituents
M1	522	SNARE-binding	*SNAP25, STX1A, STXBP1, SYT1A, VAMP2*
M2	410	death receptor activity	*TNFRSF1A, TCIRG1, PXN, LRP10, RAB13*
M3	240	NMDA glutamate receptor activity	*AHDC1, PCDHGC5, PCDHGB3, PCDHGB5, PCDHGA1*
M4	208	collagen binding	*FAM20A, PCOLCE, FOXC1, AOX1, OLFML2A*
M5	110	coA ligase activity	*SOX9, EDG1, EMX2, SLC1A3, ACSBG1*
M6	80	olfactory receptor activity	*IGSF2, KRTAP20-1, RBMY2FP, EVC, CYP7B1*
M7	71	cytokine/interferon receptor binding	*PRAMEF10, OR4K17, CLDN18, RP11-297H3.4, LOC728676*
M8	53	myosin V binding	*RAB3A, HPCA, C12orf53, MAPRE3, LOC442211*
M9	28	kinase binding	*EFS, PPP1R14A, C11orf9, DAAM2, RASGRP3*
M10	22	TGF beta binding	*CHST13, LOC374973, RNF126P1, CABC1, OR7G3*
M11	22	ATPase activity	*ABCA2, SOX10, MYO9B, PCTK3, PLXNB3*

Genes were organized into modules based on expression similarity using Pearson correlation and the Dynamic Tree Cut package in webCEMiTool, with a minimum of 20 genes per module. Molecular function for each module was assigned based on the top functional group. Key constituents are hub or top-ranking genes in each module. Complete gene list for each module can be found in Supplementary Table 2.

Among the M1 DEGs with FC of at least 10%, 198 were downregulated and 100 were upregulated. As shown in the volcano plot of 522 DEGs (Fig. 3C), *SNAP-25* (FC = −1.26) and *SYN2* (FC = −1.42), another gene integral for synaptic vesicle release, were among the most prominently downregulated M1 DEGs, while *GFAP* and *CRYAB*, both of which are involved in gliosis and glial-related pathology associated with neurodegeneration,^13^^,^^31^^,^^32^ were among the most prominently upregulated M1 DEGs.

Next, GO enrichment analysis was performed to determine the molecular function of DEGs in Module 1. SNARE-binding was identified as the most enriched pathway among the 298 significant M1 DEGs, including *SNAP-25, VAMP2, STX1A, STXBP1, SYP, NSF, UNC13A* and *NAPB*, which interact with other M1 DEGs that are enriched in cytoskeleton regulation and calmodulin binding (Fig. 3D). GO analysis of M1 downregulated DEGs confirmed that the top categories include SNARE- or syntaxin-binding, structural constituent of cytoskeleton and calmodulin binding (Fig. 3E, Supplementary Table 5). GO analysis of M1 upregulated DEGs revealed that the top categories include l-ascorbic acid binding, cell adhesion molecule binding, and integrin binding (Fig. 3F, Supplementary Table 5). These findings support our previous identification of SNARE complex genes as the top-ranking downregulated clusters in PFC of human Alzheimer’s disease, and further highlights the dysregulation of SNARE-mediated exocytotic function in Alzheimer’s disease.

GO analysis of Modules 2–11 DEGs was also carried out to better understand other functional differences between Alzheimer’s disease and control groups (Supplementary Table 6). In addition to M1, module 3 (M3) also has synaptic transmission pathways, such as NMDA glutamate and ionotropic receptor activity as top molecular functions changed in Alzheimer’s disease. Modules 2 and 7 highlight an upregulated immune response, which is consistent with Fig. 1B. Other modules show functional changes broadly related to signal transduction and ion channel activity. These data highlight a range of aberrant pathways in Alzheimer’s disease that are driven by gene dysregulation, and provide a framework that allows for future studies to target gene networks as opposed to individual gene changes.

### Single-nucleus transcriptomics reveal the loss of synaptic genes in PFC excitatory and inhibitory neurons

**Figure 4:**
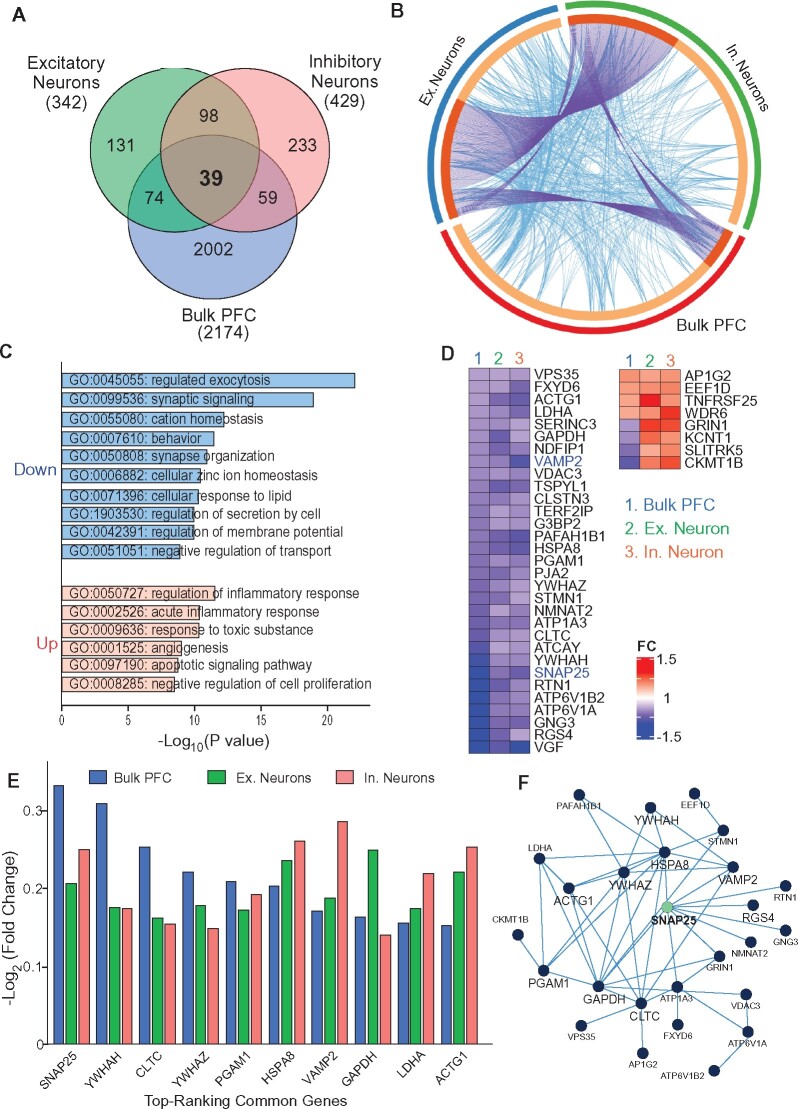
**Comparison of bulk and single neuron-specific genomic alterations of Alzheimer’s disease.** (**A**) Venn diagram representing significant DEGs from bulk PFC or single excitatory and inhibitory PFC neurons. (**B**) Circos plot of all three DEG lists from bulk PFC or single excitatory (Ex.) or inhibitory (In.) neurons, where connecting blue lines represent genes within the same enriched ontology pathway, and purple lines represent linked identical genes between groups. (**C**) GO enrichment analysis of common biological pathways from bulk PFC or single Ex. or In. neurons. (**D**) Heatmaps representing the fold change (FC) of common DEGs from bulk PFC or single Ex. or In. neurons. (**E**) Bar graphs representing FC values of top-ranking common DEGs. (**F**) Interaction network of common DEGs.

To find out whether the genetic alterations identified in RNA microarray are present in neurons, we compared bulk DEGs to neuronal DEGs acquired from single-nucleus RNAseq of Alzheimer’s disease samples.^14^ Using the same cutoffs for significance (*P* < 0.05; |FC| > 10%), we identified 39 common genes differentially expressed in all three groups—PFC bulk (2174 DEGs), PFC excitatory neurons (342 DEGs) and PFC inhibitory neurons (429 DEGs) (Fig. 4A). As shown in the Circos plot (Fig. 4B), DEGs in PFC excitatory and inhibitory neurons exhibited a greater overlap with each other as indicated by more identical altered genes (purple lines), when compared with bulk PFC DEGs. All three groups exhibited common biological pathways as indicated by connecting blue lines.

The most enriched convergent pathways among the three groups are exocytosis and synaptic signalling (Fig. 4C), confirming the loss of presynaptic function in PFC excitatory and inhibitory neurons. Among the 39 common DEGs (Fig. 4D), 17 were identified to be synaptic, predominately in the presynaptic subcluster, and the majority (31) of these common DEGs was consistently downregulated in all three groups (Supplementary Table 7).

Hub analysis was performed to identify top ranking genes that are common to bulk and specific neuronal groups with the highest intra-molecular connectivity. This unbiased ranking system allows us to identify genes that are most central within the three groups of DEGs. Interestingly, *SNAP-25* and *VAMP2* were among the central hub genes identified, highlighting the loss of SNARE-associated genes in cortical excitatory and inhibitory neurons (Fig. 4E). Two hub genes that encode 14-3-3 proteins, *YWHAH* and *YWHAZ*, were also consistently downregulated in all three groups. 14-3-3 proteins are abundant synaptic binding proteins mediating diverse processes, such as protein trafficking, glutamatergic transmission and cell signalling.^33^^,^^34^ Another hub gene decreased in all three groups, *HSPA8,* encodes a heat shock protein belonging to the heat shock protein 70 family, which is involved in protein homeostasis and signal transduction.^35^^,^^36^ The downregulation of common hub genes in bulk and neuronal groups highlights a network of dysregulated synaptic molecules involved in exocytotic function and neurotransmission in PFC of human Alzheimer’s disease (Fig. 4F).

### Gene alterations in Alzheimer’s disease are inversely related to protein changes required for CS

Next, we sought to determine whether the loss of synaptic genes in cortical neurons of Alzheimer’s disease humans is linked to cognitive impairment. To do so, we first searched for markers in PFC that are associated with CS in normal ageing. We acquired previously published human proteomic data from dorsolateral PFC of two longitudinal cohorts, Banner (104 participants tracked for 14 years) and BLSA (39 participants tracked for 20 years).^15^ In this study, long-term cognitive assessment data were used to develop a cognitive trajectory score for each subject that correlates with CS or cognitive decline. The authors then, used label-free proteomic analysis, where protein abundance from each subject was correlated with the associated cognitive trajectory score. This proteomic analysis revealed 569 unique proteins identified as being necessary for CS.^15^

**Figure 5:**
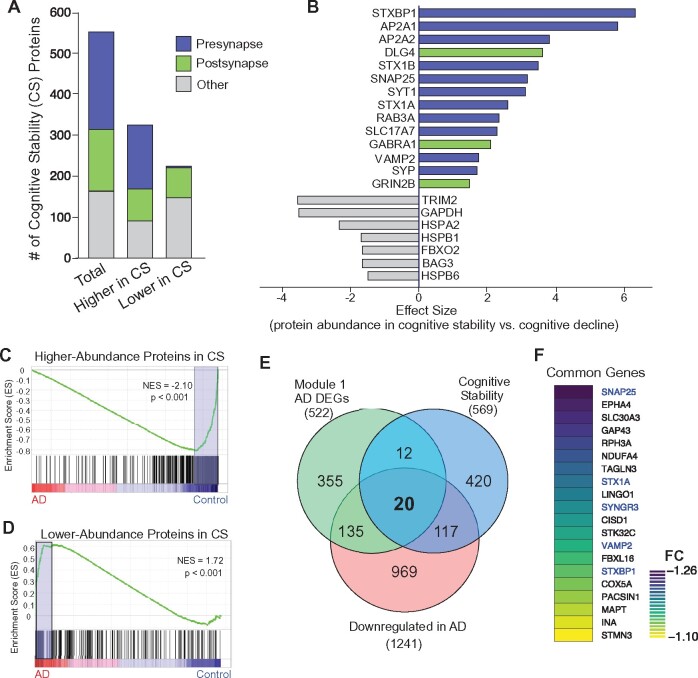
**Correlation between cognitive stability (CS) proteins and Alzheimer’s disease-altered genes**. (**A**) Bar graph showing the classification of CS proteins (569 total, 344 higher-abundance in CS, 225 lower-abundance in CS). (**B**) Bar graph displaying bi-directional effect size in key differentially expressed CS proteins in humans with CS, compared to patients with cognitive decline. Proteins with positive or negative effect sizes represent those higher-abundance or lower-abundance CS proteins, respectively. (**C, D**) GSEA plots of ranked Alzheimer’s disease DEGs, compared to higher-abundance (**C**) or lower-abundance (**D**) CS proteins. (**E**) Venn diagram representing Module 1 Alzheimer’s disease DEGs (522), CS proteins (569), and downregulated genes in Alzheimer’s disease (1241). (**F**) Heatmap of fold-change (FC) values for the 20 common targets shown in **E**. Synaptic vesicle genes are highlighted in blue.

Of these 569 identified proteins associated with cognitive trajectory, 344 proteins had increased abundance in CS (refer as higher-abundance CS proteins), while 225 proteins had a decreased abundance in CS (refer as lower-abundance CS proteins). As shown in Fig. 5A and B, the majority of higher-abundance CS proteins from this study are involved in synaptic function, including presynaptic markers associated with SNARE-mediated exocytosis (e.g. STXBP1, STX1B, SNAP-25, SYT1, STX1A, RAB3A, VAMP2, SYP), vesicle endocytosis (e.g. AP2A1, AP2A2), postsynaptic receptors and anchoring proteins (e.g. DLG4, GABRA1, GRIN2B), while the lower-abundance CS proteins were mainly inflammatory and apoptotic-related markers, including TRIM2, GAPDH, HSPA2, HSPB1 and FBXO2. These data have demonstrated the key role of high expression of synaptic proteins, especially SNARE-complex components, in maintaining CS.

Using GSEA, we discovered an inverse relationship between CS protein markers and Alzheimer’s disease genomic markers (Fig. 5C and D). Our analysis revealed that those genes encoding higher-abundance proteins in CS were significantly decreased in Alzheimer’s disease (NES = −2.10, *P* < 0.001, Fig. 5C). On the other hand, those genes encoding lower-abundance proteins in CS were significantly increased in Alzheimer’s disease (NES = 1.72, *P* < 0.001, Fig. 5D). It suggests that the gene alteration identified in Alzheimer’s disease is causally linked to the loss of CS.

Examining the Alzheimer’s disease DEGs in Module 1 (522), CS proteins (569) and downregulated genes in Alzheimer’s disease (1241), we identified 20 common targets (Fig. 5E). Synaptic vesicle genes, including *SNAP-25*, *STX1A, SYNGR3, VAMP2* and *STXBP1*, are among the common DEGs overlapping within the three groups, with *SNAP-25* having the greatest loss of expression (Fig. 5F). These genes represent a molecular network that could be targeted for therapeutic rescue of synaptic and cognitive function.

### The loss of presynaptic genes is confirmed in Alzheimer’s disease humans

**Figure 6:**
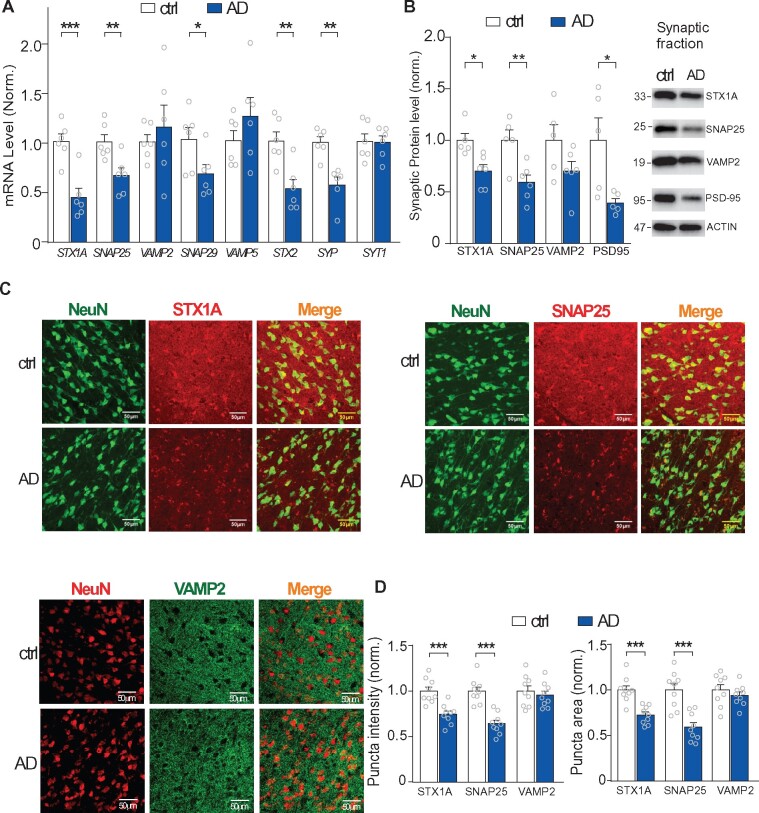
**Confirmation of the loss of synaptic genes in PFC of Alzheimer’s disease humans**. (**A**) Bar graphs showing qPCR data of the selected synaptic genes in PFC (BA10) of postmortem tissues from Alzheimer’s disease patients vs. control (Ctrl: *n* = 6, Alzheimer’s disease: *n* = 6). (**B**) Representative Western blots and quantification of synaptic proteins (STX1A, SNAP25, VAMP2 and PSD95) in the synaptic fraction of PFC from Alzheimer’s disease patients vs. control subjects (Ctrl: *n* = 5, Alzheimer’s disease: *n* = 6). (**C**) Representative immunofluorescence images of synaptic proteins (STX1A, SNAP25, VAMP2), co-stained with the neuronal marker NeuN, in PFC from Alzheimer’s disease patients vs. control subjects. scale bar, 50 µm. (**D**) Quantification of the fluorescence average intensity and puncta area of synaptic proteins in Alzheimer’s disease and controls (*n* = 9 slices from 3 humans each group). All data are presented as mean ± SEM. In all figures, *: *P* < 0.05, **: *P* < 0.01, ***: *P* < 0.001, *t*-test.

We then examined whether the high-ranking presynaptic genes downregulated in genomic sequencing are indeed decreased in Alzheimer’s disease human brains (Supplementary Table 8). Quantitative PCR (qPCR) was first conducted to examine the selected synaptic genes in PFC of postmortem tissues from Alzheimer’s disease humans and control subjects. As shown in Fig. 6A, the mRNA level of *SNAP25, STX1A, SNAP29, STX2* and *SYP* was significantly decreased in Alzheimer’s disease patients, compared to control subjects [*SNAP25*, *t*_(10)_ = 3.2, *P* < 0.01; *STX1A*, *t*_(10)_ = 4.7, *P* < 0.001; *SNAP29*, *t*_(10)_ = 2.3, *P* < 0.05; *STX2*, *t*_(10)_ = 3.7, *P* < 0.01; *SYP*, *t*_(10)_ = 4.5, *P* < 0.01, *t*-test], consistent with transcriptomic data from the large-scale human samples (129 Alzheimer’s disease and 101 controls). However, we did not detect the significant reduction of *VAMP2* mRNA in our small-scale human samples [*t*_(10)_ = 0.6, *P* = 0.52, *t*-test, 6 Alzheimer’s disease and 6 controls]. The discrepancy could be due to sample size differences and human sample variations.

Next, Western blotting was performed to examine the selected proteins in the synaptic fraction from PFC of Alzheimer’s disease humans and controls. As shown in Fig. 6B, the protein level of STX1A, SNAP25 and PSD-95 at synapses was significantly decreased in Alzheimer’s disease patients [STX1A, *t*_(9)_ = 3.2, *P* < 0.05; SNAP25, *t*_(9)_ = 3.4, *P* < 0.01; PSD-95, *t*_(9)_ = 2.7, *P* < 0.05, *t*-test], while VAMP2 protein level was not significantly changed [*t*_(9)_ = 1.7, *P* = 0.11, *t*-test]. The full blots of all examined samples are shown in Supplementary Fig. 2.

To examine the alteration of these synaptic proteins in neurons, we further performed immunostaining of SNARE-complex core proteins with the neuronal marker NeuN in human PFC slices. As shown in Fig. 6C and D, the puncta intensity and area of STX1A and SNAP25 in PFC neurons (NeuN+) were markedly reduced in Alzheimer’s disease patients [Intensity, STX1A, *t*_(16)_ = 4.7, *P* < 0.001; SNAP25, *t*_(16)_ = 6.0, *P* < 0.001; Area, STX1A, *t*_(16)_ = 4.9, *P* < 0.001; SNAP25, *t*_(16)_ = 4.9, *P* < 0.001, *t*-test], while VAMP2 puncta intensity and area were not significantly changed [Intensity, *t*_(16)_ = 0.6, *P* = 0.54; Area, *t*_(16)_ = 0.8, *P* = 0.40, *t*-test]. These data have confirmed the robust reduction of some core SNARE binding elements in PFC neurons of Alzheimer’s disease humans.

## Discussion

In this study, we performed transcriptomic analyses of postmortem prefrontal cortical tissue from Alzheimer’s disease and non-demented human patients. While previous studies have reported the complex Alzheimer’s disease-associated genetic alterations in different cell types,^1^^,^^13^^,^^14^ we aimed to identify the most prominent changes in gene networks, molecular pathways and biological processes that are directly responsible for cognitive decline in Alzheimer’s disease. Using multiple bioinformatics approaches, including module co-expression analysis, MCC gene ranking in common pathways, and GSEA analysis between genomic and proteomic datasets, we have revealed the loss of SNARE complex-related genes in cortical neurons as a potential key factor causing synaptic and cognitive deficits in Alzheimer’s disease.

Our analyses of 230 human samples reliably identified two major categories of genetic changes in Alzheimer’s disease—the downregulation of genes involved in synaptic function and the upregulation of genes involved in immune response pathways. Since the ‘early-pathology’ Alzheimer’s disease group mainly exhibits the downregulation of genes exclusively in neurons,^13^ we have focussed on the analyses of downregulated genes in Alzheimer’s disease that are potential targets for early intervention. The diminished gene category is most strongly associated with those involved in presynaptic vesicle docking, endocytosis, and exocytosis. In addition, genes involved in glutamatergic and GABAergic transmission are also among the top-ranking downregulated list.

Through co-expression modular analysis, we identified that the genes diminished in Alzheimer’s disease are most enriched in module 1 (M1) that contains SNARE-binding complex genes, including the core SNARE genes, *SNAP-25*, *STX1A* and *VAMP2*. These findings were corroborated in excitatory and inhibitory neurons, when compared to a single-cell gene expression dataset from PFC of a different Alzheimer’s disease cohort.^14^ Interestingly, proteomics studies of postmortem human brains in the PFC of patients with Alzheimer’s disease, Parkinson's disease with dementia, dementia with Lewy bodies and older adults without dementia also found that selected synaptic proteins were significantly lost in the various dementia groups, which was significantly correlated with the rate of cognitive decline.^37–39^

In addition to M1, module 8 (M8), which is involved in myosin V-mediated transport is also downregulated in Alzheimer’s disease. Myosin V is a calcium-activated actin-associated molecular motor protein that interacts with syntaxin-1A and is involved in vesicle transport and docking.^40–42^ Two of the hub genes in M8 central to synaptic function are *HPCA* and *RAB3A,* both encoding calcium-dependent proteins. HPCA is a calcium sensor primarily expressed in pyramidal neurons, which has been implicated in synaptic plasticity and memory processes.^43–46^ RAB3A is a small GTP-binding protein regulating synaptic vesicle exocytosis,^47^ and is reduced in the brain of patients with Alzheimer’s disease and other dementia.^37^^,^^48^ The loss of genes in M1 and M8 may directly underlie synaptic dysfunction in Alzheimer’s disease, the best correlate of cognitive impairment.

Among the large number of genes altered in Alzheimer’s disease, a key question is what gene changes are the potential causal factor for cognitive decline. A proteomic study of cognitive trajectory (Wingo et al., 2019) has revealed 344 proteins with the increased abundance in individuals with CS, many of which are involved in pre- and post-synaptic function, including presynaptic SNARE complex components, as well as postsynaptic receptors or anchoring proteins. It also revealed 225 proteins with the decreased abundance in individuals with CS, the majority of which are involved in apoptosis and inflammation, including heat-shock proteins and E3 ubiquitin ligases (Wingo et al., 2019). Employing GSEA analysis, we found that gene alterations in Alzheimer’s disease are inversely related to the higher- or lower-abundance proteins associated with CS. Synaptic vesicle genes, including *SNAP-25, STX1A, VAMP2and STXBP1*, are on the top list of common targets involved in Alzheimer’s disease and cognitive trajectory, further suggesting that the transcriptional downregulation of synaptic genes is directly linked to cognitive decline in Alzheimer’s disease. Thus, our study has uncovered a well-defined network of genes that could be potential targets for early disease intervention.

## Supplementary material

Supplementary material is available at *Brain Communications* online.

## Supplementary Material

fcab123_Supplementary_Data

## Abbreviations

CS =: cognitive stability
DEGs =: differentially expressed genes
ES =: enrichment score
FC =: fold change
GSEA =: gene set enrichment analysis
PFC =: prefrontal cortex
qPCR =: quantitative polymerase chain reaction
