# Supplementary material for: Transcriptomic analysis of human brains with Alzheimer’s disease reveals the altered expression of synaptic genes linked to cognitive deficits
Source: Brain Commun. 2021 Jun 3;3(3):fcab123. doi: 10.1093/braincomms/fcab123 (PMC8374979; doi:10.1093/braincomms/fcab123)
Supplement: fcab123_Supplementary_Data [file fcab123_supplementary_data.zip › Supplementary figures.pdf]

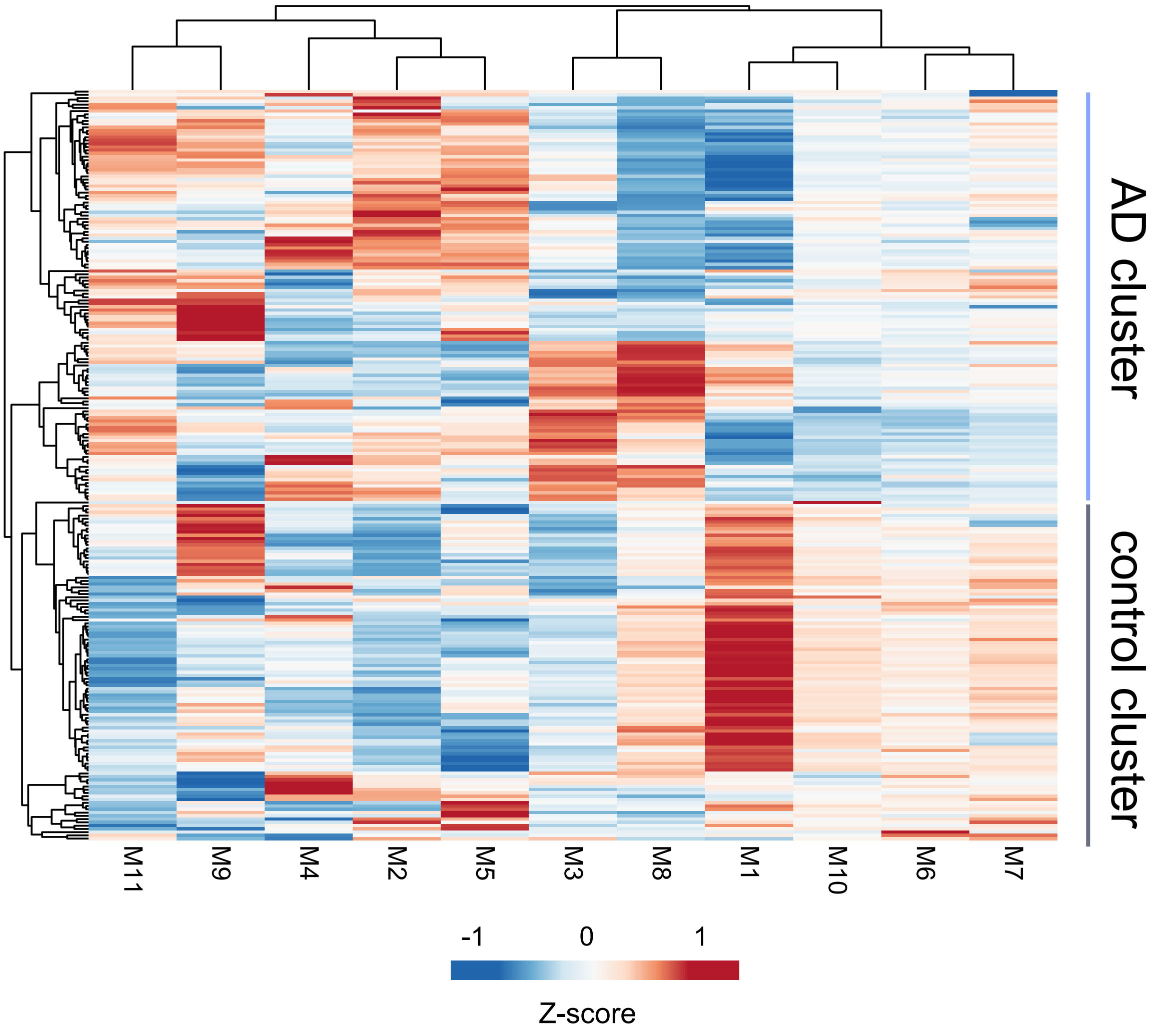

Supplementary Figure 1. Normalized eigengene expression for gene co-expression modules. Heatmap showing normalized eigengene expression values for 230 patient samples (129 AD and 101 control) across 11 gene co-expression modules associated with **Fig. 3B**.

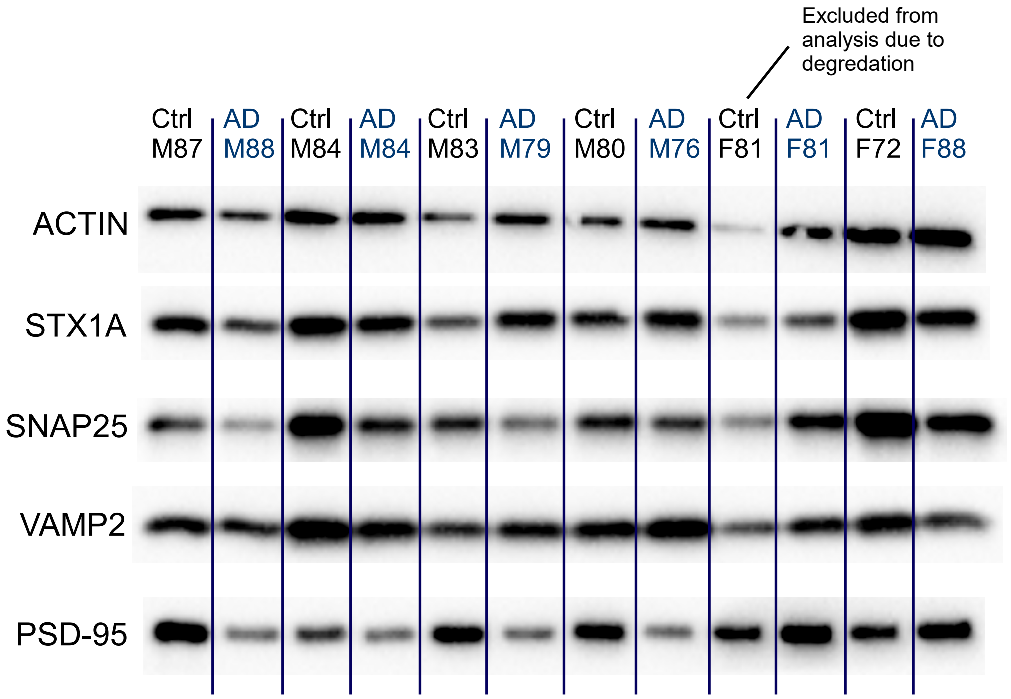

Supplementary Figure 2. Protein expression levels in PFC human AD vs control samples. Full Western blots for **Fig. 6B**, showing age and sex matched AD and control samples.
