# Supplementary material for: Transcriptomic analysis of human brains with Alzheimer’s disease reveals the altered expression of synaptic genes linked to cognitive deficits
Source: Brain Commun. 2021 Jun 3;3(3):fcab123. doi: 10.1093/braincomms/fcab123 (PMC8374979; doi:10.1093/braincomms/fcab123)
Supplement: fcab123_Supplementary_Data [file fcab123_supplementary_data.zip › Original Submission.pdf]

OXFORD UNIVERSITY PRESS | Brain Communications

Transcriptomic Analysis of Human Brains with Alzheimer's Disease Reveals the Key Determinant for Cognitive Deficits

Journal:	*Brain Communications*
Manuscript ID	BRAINCOM-2020-418
Manuscript Type:	Original Article
Date Submitted by the Author:	23-Dec-2020
Complete List of Authors:	Williams, Jamal; State University of New York at Buffalo, Department of Physiology and Biophysics<br>Cao, Qing; State University of New York at Buffalo, Department of Physiology and Biophysics<br>Yan, Zhen; State University of New York at Buffalo, Department of Physiology and Biophysics
Keywords:	Alzheimer's disease, transcriptomic analyses, synaptic genes, SNARE complex, cognitive deficits

SCHOLARONE™
Manuscripts

Transcriptomic Analysis of Human Brains with Alzheimer's Disease Reveals the Key Determinant for Cognitive Deficits

Jamal B. Williams, Qing Cao, Zhen Yan*

Department of Physiology and Biophysics, State University of New York at Buffalo, Jacobs School of Medicine and Biomedical Sciences, Buffalo, NY 14203, USA

*: Correspondence should be addressed to Dr. Zhen Yan, Department of Physiology and Biophysics, State University of New York at Buffalo, Jacobs School of Medicine and Biomedical Sciences, 955 Main St., Buffalo, NY 14203. Email: zhenyan@buffalo.edu

For Review Only

Abstracts: 146 words

Figures: 7

Tables: 1

Supplementary Tables: 4

Abstract

Alzheimer's disease (AD) is a progressive neurodegenerative disorder associated with memory loss and impaired executive function. The molecular underpinnings causing cognitive deficits in AD are loosely understood. Here, we performed cross-study large-scale transcriptomic analyses of postmortem prefrontal cortex (PFC) derived from AD patients to reveal the role of aberrant gene expression in this disease. We identified that one of the most prominent changes in PFC of AD humans was the downregulation of genes in excitatory and inhibitory neurons that are associated with synaptic functions, particularly the SNARE-binding complex, which is essential for vesicle docking and neurotransmitter release. Comparing genomic data of AD with proteomic data of cognitive trajectory, we found that many of the lost synaptic genes in AD encode hub proteins whose increased abundance is required for cognitive stability. This study has revealed potential molecular targets for therapeutic intervention of cognitive decline associated with AD.

Keywords: Alzheimer's disease; human; transcriptomic analyses; synaptic genes; SNARE complex; cognitive deficits

For Review Only

Introduction

Alzheimer's disease (AD), the most prevalent neurodegenerative disorder, is marked by the progressive decline of memory and cognitive function. AD brains exhibit multiple pathological features, including amyloid plaques, neurofibrillary tangles, astrogliosis, microglia activation, and the losses of neurons, neuropil, and synaptic elements. However, it is still unclear what are the causative factors for cognitive decline in AD. Genomic studies of AD human samples have revealed the transcriptional changes of a large number of genes involved in a wide range of pathways, including inflammation, apoptosis, and synaptic function (Miller et al., 2013; Zhang et al., 2013; Mostafavi et al., 2018; Raj et al., 2018; Bai et al., 2020). The transcriptomic alteration is also accompanied by aberrant epigenetic modification in AD (De Jager et al., 2014; Gjoneska et al., 2015; Klein et al., 2019; Nativio et al., 2018; Nativio et al., 2020). The advancement of genomic techniques has enabled the detection of single-cell transcriptional changes (Lake et al., 2016; Lake et al., 2018; Macosko et al., 2015; Mathys et al., 2019; Zhong et al., 2018; Zhou et al., 2020). While numerous changes in genes and pathways are reported in AD, how the seemingly divergent findings coalesce have yet to be elucidated.

A big challenge for AD treatment is the identification of key molecules that cause cognitive impairment at the early stage before global neurodegeneration. Single-cell RNAseq of prefrontal cortex of humans with varying degrees of AD pathology show that nearly all perturbed genes (>80% downregulated) in the 'early-pathology' group occur only in excitatory and inhibitory neurons, while most of the altered genes in 'late-pathology' group are upregulated across cell types and primarily involved in global stress response (Mathys et al., 2019). An unbiased proteome-wide association study of cognitive trajectory has found that cognitive stability is positively correlated with the increased abundance of proteins involving synaptic functions regardless of neurodegenerative pathologies (Wingo et al., 2019). It prompts us to contemplate that the loss of synaptic genes important for neuronal plasticity in early-stage AD is directly responsible for synaptic dysfunction and cognitive decline.

In this study, we performed in-depth bioinformatic analyses of bulk and single-cell transcriptomic data from human AD brains, and revealed the loss of presynaptic and postsynaptic genes involved in vesicle release and synaptic transmission as the most prominent changes in prefrontal cortex (PFC) of AD humans. Particularly, we identified a systemic loss of genes associated with the SNARE-binding complex, which is essential for vesicle docking and neurotransmitter release (Baker and Hughson, 2016; Fasshauer et al., 1998; Südhof and Rothman, 2009). SNARE complex-related genes were also among the top-ranking downregulated genes in excitatory and inhibitory neurons from AD patients. Interestingly, we

For Review Only

found that many of the lost genes in AD encode hub proteins whose increased abundance is required for cognitive stability in normal aging (Wingo et al., 2019). These genes provide promising targets for the intervention of cognitive trajectory in AD.

Materials and Methods

Genomic Data Processing and Differential Expression Analysis

The 230 human dorsolateral PFC samples were separated based on a final clinical diagnosis of late-onset AD (129 samples) or being healthy (101 samples) followed by postmortem pathological confirmation. The RNA microarray dataset GSE44770 (Zhang et al., 2013) was acquired using NCBI's public database GEO, followed by processing and analysis in Phantasus (https://artyomovlab.wustl.edu/phantasus/). Differential expression of genes between AD and control groups were obtained using the Limma analysis package. Genes with differential expression of p-values below 0.05 and |fold change (FC)| above 1.1 were considered as being significantly different.

GO Enrichment Analysis

Enrichment analysis of differentially expressed genes (DEGs) from the RNA microarray dataset was performed using Metascape (http://metascape.org), as previously reported (Zhou et al., 2019). In addition, the Metascape multi-gene-list meta-analysis tool was used to compare common functional pathways among DEGs from bulk tissues or single excitatory or inhibitory neurons. In addition to enrichment bar graphs, Circos plots were also used to display the individual connections of genes and pathways between groups. Additionally, Enrichr (https://maayanlab.cloud/Enrichr/) was used to characterize molecular function changes in modular co-expression analysis. To investigate dysregulated synaptic organization in AD, we submitted the downregulated RNA microarray dataset to the synaptic ontology database SynGO (https://www.syngoportal.org/), which was used to identify the enriched synaptic components in AD DEGs.

Hub Genes and Network Interactome

Gene sets in which hub analysis was performed were first uploaded to Cytoscape, an open source network visualization software (Shannon et al., 2003). An interactome of these genes were then generated using the STRING platform, a database for protein-protein interactions (Szklarczyk et al., 2019). Then, using the Cytoscape plugin *cytoHubba*, hub genes for each input dataset were identified and ranked (Chin et al., 2014). The Maximal Clique

For Review Only

Centrality (MCC) algorithm was used to rank top-defined nodes (genes) in each dataset. The results were then visualized in Cytoscape.

Modular Co-Expression Analysis

Modular analysis was performed using webCEMiTool to identify distinct dysregulated co-expression networks that highlight functional changes in AD (Cardozo et al., 2019). In brief, gene expression data from the RNA microarray dataset was uploaded to CEMiTool (https://cemitool.sysbio.tools/analysis), and filtered using an unsupervised sorting algorithm. 12 modules were then generated, each containing genes with similar expression patterns.

Gene Set Enrichment Analysis (GSEA)

To compare gene expression profiles identified in our RNA microarray dataset (GSE44770) to proteins required for cognitive stability (CS), GSEA was performed using GSEA v.4.0.3 (http://www.broadinstitute.org/gsea). First, the cognitive stability markers identified by proteomic analysis (Wingo et al., 2019) were separated into two files (higher-abundance in CS and lower-abundance in CS), formatted as *gmt* files and uploaded as two *Gene Set Databases*. Then the microarray genes were ranked and formatted as a *rnk* file and uploaded as a *RankedGeneList*. These pre-ranked genes were then run on the GSEA platform, where each gene was compared to the cognitive stability dataset, accumulating a running-sum statistic. This result is represented as an enrichment score (ES), and signifies if a gene set (in our case the cognitive stability markers) is positively or negatively correlated with the gene list (RNA-microarray gene set). However, the standard value to consider is normalized enrichment score (NES), which accounts for variability in gene set sizes.

To interpret the GSEA enrichment plots, emphasis should be directed to the peak of the green curve, which represents the ES. The peak will be above 0 for positive ES values, and below 0 for negative ES values. The blue shaded regions to the left or right of the peak is called the *leading edge subset* and represents genes (vertical black lines) within the dataset that contribute to this enrichment score.

Quantitative real-time PCR

Postmortem human frontal cortex (Brodmann's Area 10) from AD patients and control subjects were provided by NIH NeuroBioBank. Upon arrival, tissue was stored in a -80°C freezer until used for RNA and protein extraction. RNA was extracted from human postmortem tissue by using TRIzol RNA Isolation Reagents (Invitrogen). RNA concentration was measured

https://mc.manuscriptcentral.com/braincom

For Review Only

with Nanodrop and equal amounts of RNA (1μg) were reversed transcribed using iScript reverse transcription kit (Bio-Rad). Quantitative real-time PCR was performed with SYBR-Green-based reagents, detected by the iCycler iQ™ Real-Time PCR Detection System and iQ™ Supermix (Bio-Rad) according to the manufacturer's instructions. GAPDH was used as the housekeeping gene to normalize the expression of target genes in samples. Fold changes in the target genes were determined by normalizing raw fluorescent values of AD group to control group using the following formula: Fold change = $2^{-\Delta(\Delta C_T)}$, where $\Delta C_T = C_{T(target)} – C_{T(GAPDH)}$, and $\Delta(\Delta C_T) = \Delta C_{T(AD)} - \Delta C_{T(control)}$. C_T (threshold cycle) is defined as the fractional cycle number at which the fluorescence reaches 10x the standard deviation of the baseline. A total reaction mixture of 18 μl was amplified in a 96-well thin-wall PCR plate (Bio-Rad) using the following PCR cycling parameters: 95°C for 5 min followed by 40 cycles of 95°C for 30 secs, 55°C for 30 secs, and 72°C for 60 secs. Primers sequences used for PCR were as follows:
STX1A (F: CGTGGAGAGCCAGACTATGT; R: CTGGAGTGGAGTGGCAGTTT), SNAP25 (F: TCCCGAGAAGCCCAGGTAAG; R: GCAGCTCACCTCGAAAACAC), VAMP2 (F: GTCTCTCCTGCGTTCCCC; R: CGACCTCACAGATGCGATCC), SNAP29 (F: CTGGCCCTCATGTACGAGTC; R: AGGGTGCCATTCTGTTCAGG), VAMP5 (F: AATAGAGTTGGAGCGGTGCC; R: AGGAGTTGGTCTGAACGCTG), STX2 (F: AAAGGCCGCATCCAGCG; R: TGCTGGTCTCCAGCTTCAT), SYP (F: GTCAGTTCCGGGTGGTCAAG; R: AAGTACACTTGGTGCAGCCT), SYT1 (F: GTGGTGGTAACTGTTTTGGACT; R: ATGTCTGACCAGTGTCGCAG).

Western blotting of synaptic proteins

Synaptic protein isolation was performed as follows. Briefly, the human postmortem PFC tissue was lysed and homogenized in ice-cold lysis buffer (10 ml/g, 15 mM Tris, pH 7.6, 0.25 M sucrose, 1 mM EGTA, 2 mM EDTA, 25 mM NaF, 10 mM $Na_4P_2O_7$, 10 mM Na_3VO_4, 1 mM PMSF, and protease inhibitor tablet). After centrifugation at 800 × g for 5 min to remove nuclei and large debris, the remaining supernatant was subjected to 10,000 × g centrifugation for 10 min. The crude synaptosome fraction (pellet) was suspended in lysis buffer containing 1% Triton X-100 and 300 mM NaCl, homogenized again, and centrifuged at 16,000 × g for 15 min. Triton insoluble fraction, which mainly includes membrane-associated proteins from synpatosomes, was dissolved in 1% SDS. Protein concentration was measured by the BCA assay (Thermo Fisher). Samples were boiled in 2× SDS loading buffer for 5 min, and separated on 7.5% SDS-PAGE. Western blotting of synaptic proteins was performed by incubating overnight with the following primary antibodies: STX1A (1:500, Proteintech, 66437-1-Ig), SNAP25 (1:500,

For Review Only

Proteintech, 60159-1-Ig), VAMP2 (1:500, Proteintech, 10135-1-AP), PSD95 (1:500, Cell Signaling Technology, 2507) and GAPDH (1:2000, Cell Signaling Technology, 5174). After the incubation with a secondary antibody (horseradish peroxidase-conjugated), ECL reaction was performed using enhanced chemiluminescence substrate (Thermo Scientific). Luminescence was detected by Chemidoc XRS system (Bio-Rad) and density of blots was quantified by ImageJ software (NIH).

Immunohistochemistry

Human postmortem PFC tissue was cut into a small chunk (1 cm^3), followed by fixing in 4% paraformaldehyde at 4°C overnight before cutting into 50 μm slices. Sections were washed in PBS (3 times, 15 min each) and blocked in 5% goat serum (1 hour, RT), then stained overnight with STX1A (1:500, Proteintech, 66437-1-Ig) and NeuN (1:500, Novus Biologicals, NBP1-77686), or SNAP25 (1:500, Proteintech, 60159-1-Ig) and NeuN (1:500, Novus Biologicals, NBP1-77686), or VAMP2 (1:500, Proteintech, 10135-1-AP) and NeuN (1:500, Millipore, MAB377) at 4°C. After washing three times in PBST (1x PBS, 0.05% Tween® 20), slices were incubated with two secondary antibodies (Alexa Fluor 488, 1:1000, Thermo Fisher Scientific, A-11008) and (Alexa Fluor 594, 1:1000, Thermo Fisher Scientific, A-11032) for 1 hr at room temperature, followed by three washes with PBST. Slices were mounted on slides with VECTASHIELD mounting media (Vector Laboratories). Images were acquired by Leica TCS SP8 Confocal Microscope and analyzed by FIJI Image J (NIH). All specimens were imaged under identical laser power and analyzed with identical parameters.

Statistical analysis

Data were analyzed with GraphPad Prism v.6 (GraphPad). All values are mean ± SEM. Differences between two groups were assessed with unpaired Student's *t*-test with unequal variance.

Data Availability

The data used to compare gene expression between AD and healthy controls were from the RNA microarray public dataset deposited in GEO under the accession number GSE44770 (Zhang et al., 2013). The data used to compare gene expression in excitatory and inhibitory neurons were from human snRNA sequencing data deposited in Synapse (https://doi.org/10.7303/syn21125841) by Zhou et al., 2019. The data of cognitive trajectory proteins were obtained from the large-scale proteomic study by Wingo et al., 2019.

For Review Only

Results

Differential Gene Expression Analysis Identifies the Loss of Synaptic Genes in PFC of AD Patients.

Postmortem brain tissue was collected from the dorsolateral PFC of 129 late-onset AD patients and 101 non-demented healthy controls from the Harvard Brain Tissue Resource Center. Patients from which these samples were acquired received a clinical diagnosis of AD or were otherwise deemed healthy. These postmortem tissues underwent extensive screening for AD pathology prior to analysis. Gene expression analyses were then conducted via RNA array hybridization technology, where age, sex, RNA integrity, postmortem interval, and sample pH were normalized accordingly, and deposited in NCBI's gene expression omnibus (GEO) database (Zhang et al., 2013).

We downloaded this microarray gene expression dataset, then calculated and examined the value distribution for all 230 samples. The data displayed a median-centered distribution, suggesting that the dataset is normalized and cross-comparable. Analyses of the transcriptomic data with a cutoff of $p < 0.05$ and fold change (FC) of 10% identified 2174 differentially expressed genes (DEGs) (1241 down; 933 up). GO enrichment analyses of these significant DEGs indicated that the most prominently down-regulated genes in AD were those involved in synaptic signaling, synapse organization, ion transport, neurotransmitter secretion, and glutamatergic synaptic transmission (**Figure 1A**). On the other hand, the most prominently up-regulated genes in AD were those involved in immune response activation and cell death (**Figure 1B**).

With synaptic signaling being the most significantly downregulated GO category, we further explored dysregulated synaptic pathways in AD. Among the downregulated DEGs in AD, 239 were classified as synaptic genes (**Sup. Table 1**). Synaptic enrichment analyses identified presynapse as the most enriched and abundant subcellular component downregulated in AD (**Figure 1C** and **1D**). Within the presynaptic gene cluster, synaptic vesicle-related genes were the most overrepresented subcategory (**Figure 1C**, $-\text{Log}_{10}$ p-value = 14.37).

Hub co-expression analyses were performed on the 41 downregulated synaptic vesicle-clustered genes (**Figure 1E**), and identified ten genes (*SNAP-25, SYT1, VAMP2, STX1A, SYN2, SYT11, SLC32A1, RAB3C, SV2B,* and *SH3GL2*) that were lost in PFC of AD patients, with *SNAP-25* as the top-ranking node. Interestingly, several of these hub genes encode proteins involved in SNARE complex formation, suggesting deficits in vesicular docking and exocytosis. *SNAP-25* encodes a core component of the SNARE complex, SNAP-25, which is

For Review Only

directly involved in exocytosis and neurotransmitter release (Antonucci et al., 2016; Chapman et al., 1994; Verderio et al., 2004). *SYT1* encodes the primary calcium sensor associated with SNARE complex, synaptotagmin-1, which is responsible for fast presynaptic vesicle exocytosis (Bacaj et al., 2015; Baker et al., 2018; Geppert et al., 1994b; Wu et al., 2017). Hub co-expression analyses also revealed the downregulation of synaptic genes encoding receptors, transporters and enzymes mediating glutamatergic transmission (e.g. *GRIA1, GRIA2, GRIN2A,* and *SLC17A6*) or GABAergic transmission (e.g. *GAD2, GABRB2,* and *SLC6A1*) in AD (**Figure 1F**). Together, these data indicate that the transcriptional loss of genes controlling synaptic function in PFC, particularly those encoding SNARE complex-associated proteins responsible for neurotransmitter release, is a prominent transcriptomic aberration in human AD.

Because of the enrichment of synaptic molecules in downregulated genes associated with AD, we further examined presynaptic and postsynaptic genes among the 129 late-onset AD patients and 101 non-demented healthy controls. As shown in **Figure 2A** and **2B**, many of the SNARE complex genes involved in presynaptic vesicle exocytosis were significantly downregulated in AD, including *SNAP-25*, *STX1A*, *SYT1* and *VAMP2*, while *STX2, SYN1,* and *VAMP3* were not changed. Some genes encoding postsynaptic glutamate receptors, $GABA_A$ receptors or anchoring proteins were also significantly downregulated in AD, including *GRIN2A, GRIA1, GRIA2, GABRA1, GABRB2, GRM3,* and *SHANK2*, while *GRM5, SYNGAP1, SHANK3,* and *GRIN2B* were not changed (**Figure 2C** and **2D**). The downregulation of selective synaptic genes in AD suggests that these transcriptional changes are not due to the general loss of synapses.

Gene Co-Expression Network Analysis Highlights the Loss of SNARE Complex Genes in PFC of AD Patients.

To classify gene networks that demonstrate functional enrichment in AD, we used the modular co-expression analysis, which allowed us to create weighted gene networks that unbiasedly generate interactions of genes whose expression is highly correlated to one another. Using the webCEMiTool algorithm, our AD DEGs were classified into 12 distinct modules (**Table 1; Sup. Table 2**), with module 1 (M1) being the most abundant, which consisted of 522 DEGs (**Figure 3A**). Eigengene expression (**Figure 3B**) showed that the most enriched module for downregulated genes in AD was M1 (SNARE-binding), while the most enriched module for upregulated genes in AD was M2 (Death receptor activity).

Among the M1 DEGs with fold-change of at least 10%, 198 were downregulated and 100 were upregulated. As shown in the volcano plot of 522 DEGs (**Figure 3C**), *SNAP-25* (FC =

For Review Only

-1.26) and *SYN2* (FC = -1.42), another gene integral for synaptic vesicle release, were among the most prominently downregulated M1 DEGs, while *GFAP* and *CRYAB,* both of which are involved in gliosis and glial-related pathology associated with neurodegeneration (Liu et al., 2015; Mathys et al., 2019; Wang et al., 2011), were among the most prominently upregulated M1 DEGs.

Next, GO enrichment analysis was performed to determine the molecular functions mediated by the 298 significant M1 DEGs (**Sup. Table 3**). Interestingly, SNARE-binding was identified as the most enriched molecular function (**Figure 3D**), which included *SNAP-25, VAMP2, STX1A, STXBP1, SYP, NSF, UNC13A,* and *NAPB*. Among the 129 AD patients and 101 non-demented controls, all the eight SNARE-binding genes were significantly downregulated (**Figure 3E**). In conjunction with this, these eight genes form a highly connected network (**Figure 3F**), supporting our previous identification of SNARE complex genes as the top-ranking downregulated clusters in PFC of human AD. Other M1 DEGs were enriched in the regulation of cytoskeletal structure, calcium/calmodulin binding and ion channel activity (Figure 3D and 3E).

Single-Nucleus Transcriptomics Reveal the Loss of Synaptic Genes in PFC Excitatory and Inhibitory Neurons.

To find out whether the genetic alterations identified in RNA microarray are present in neurons, we compared bulk DEGs to neuronal DEGs acquired from single-nucleus RNAseq of AD samples (Zhou et al., 2020). Using the same cutoffs for significance ($p < 0.05$; $|FC| > 10\%$), we identified 39 common genes differentially expressed in all three groups – PFC bulk (2174 DEGs), PFC excitatory neurons (342 DEGs), and PFC inhibitory neurons (429 DEGs) (**Figure 4A**). As shown in the Circos plot (**Figure 4B**), DEGs in PFC excitatory and inhibitory neurons exhibited a greater overlap with each other as indicated by more identical altered genes (purple lines), when compared with bulk PFC DEGs. All three groups exhibited common biological pathways as indicated by connecting blue lines.

The most enriched convergent pathways among the three groups are exocytosis and synaptic signaling (**Figure 4C**), confirming the loss of presynaptic function in PFC excitatory and inhibitory neurons. Among the 39 common DEGs (**Figure 4D**), 17 were identified to be synaptic, predominately in the presynaptic subcluster, and the majority (31) of these common DEGs was consistently downregulated in all three groups (**Sup. Table 4**).

Hub analysis was performed to identify the primary driver genes common to bulk and neuronal DEGs. Interestingly, *SNAP-25* and *VAMP2* were among the central hub genes

https://mc.manuscriptcentral.com/braincom

identified, highlighting the loss of SNARE-associated genes in cortical excitatory and inhibitory neurons (**Figure 4E**). Two hub genes that encode 14-3-3 proteins, *YWHAH* and *YWHAZ*, were also consistently downregulated in all three groups. 14-3-3 proteins are abundant synaptic binding proteins mediating diverse processes, such as protein trafficking, glutamatergic transmission, and cell signaling (Cornell and Toyo-Oka, 2017; Zhang and Zhou, 2018). Another hub gene decreased in all three groups, *HSPA8,* encodes a heat shock protein belonging to the heat shock protein 70 family, which is involved in protein homeostasis and signal transduction, (Mayer and Bukau, 2005; Rosenzweig et al., 2019). The common hub genes in bulk and neuronal PFC represent the loss of a network of synaptic molecules involved in exocytotic function and neurotransmission in human AD (**Figure 4F**).

For Review Only

Gene Alterations in AD Are Inversely Related to Protein Changes Required for Cognitive Stability

Next, we sought to determine whether the loss of synaptic genes in cortical neurons of AD humans is linked to cognitive impairment. To do so, we first searched for markers in PFC that are associated with cognitive stability in normal aging. We acquired human proteomic data from dorsolateral PFC of two longitudinal cohorts, Banner (104 participants tracked for 14 years) and BLSA (39 participants tracked for 20 years) (Wingo et al., 2019). Long-term cognitive assessment data were used to develop a cognitive trajectory score for each subject that correlates with cognitive stability or cognitive decline. Then, using a label-free proteomic analysis, protein abundance from each subject was correlated with the associated cognitive trajectory score. This proteomic analysis revealed 569 unique proteins identified as being necessary for cognitive stability (Wingo et al., 2019).

Of these 569 identified proteins associated with cognitive trajectory, 344 proteins had increased abundance in cognitive stability (CS) (refer as higher-abundance CS proteins), while 225 proteins had the decreased abundance in cognitive stability (refer as lower-abundance CS proteins). As shown in **Figure 5A** and **5B**, the majority of higher-abundance CS proteins are involved in synaptic function, including presynaptic markers associated with SNARE-mediated exocytosis (e.g. STXBP1, STX1B, SNAP-25, SYT1, STX1A, RAB3A, VAMP2, SYP), vesicle endocytosis (e.g. AP2A1, AP2A2), postsynaptic receptors and anchoring proteins (e.g. DLG4, GABRA1, GRIN2B), while the lower-abundance CS proteins were mainly inflammatory and apoptotic-related markers, including TRIM2, GAPDH, HSPA2, HSPB1, and FBXO2. These data have demonstrated the key role of high expression of synaptic proteins, especially SNARE-complex components, in maintaining cognitive stability**.**

Using gene set enrichment analysis (GSEA), we discovered an inverse relationship between CS protein markers and AD genomic markers (**Figure 5C** and **5D**). Our analysis revealed that those genes encoding higher-abundance proteins in CS were significantly decreased in AD (normalized enrichment score (NES) = -2.10, $p < 0.001$, Figure 5C). On the other hand, those genes encoding lower-abundance proteins in CS were significantly increased in AD (NES = 1.72, $p < 0.001$, Figure 5D). It suggests that the gene alteration identified in AD is causally linked to the loss of cognitive stability.

Examining the AD DEGs in Module 1 (522), CS proteins (569), and downregulated genes in AD (1241), we identified 20 common targets (**Figure 5E**). Synaptic vesicle genes, including *SNAP-25*, STX1A, SYNGR3, VAMP2, and STXBP1, are enriched in the list, with *SNAP-25* having the greatest loss of expression (**Figure 5F**). These genes represent a molecular network that can be targeted for therapeutic rescue of synaptic and cognitive function.

The Loss of Presynaptic Genes Is Confirmed in AD humans.

We then examined whether the high-ranking presynaptic genes downregulated in genomic sequencing are indeed decreased in AD human brains. Quantitative PCR was first conducted to examine the selected synaptic genes in PFC of postmortem tissues from AD humans and control subjects. As shown in **Figure 6A**, the mRNA level of *SNAP25, STX1A, SNAP29, STX2* and *SYP* was significantly decreased in AD patients, compared to control subjects (*SNAP25*, $t_{(10)} = 3.2$, $p < 0.01$; *STX1A*, $t_{(10)} = 4.7$, $p < 0.001$; *SNAP29*, $t_{(10)} = 2.3$, $p < 0.05$; *STX2*, $t_{(10)} = 3.7$, $p < 0.01$; *SYP*, $t_{(10)} = 4.5$, $p < 0.01$, t-test). Next, Western blotting was performed to examine the selected proteins in the synaptic fraction from PFC of AD humans and controls. As shown in **Figure 6B**, the protein level of STX1A, SNAP25 and PSD-95 at synapses was significantly decreased in AD patients (STX1A, $t_{(9)} = 3.2$, $p < 0.05$; SNAP25, $t_{(9)} = 3.4$, $p < 0.01$; PSD-95, $t_{(9)} = 2.7$, $p < 0.05$, t-test).

To examine the alteration of these synaptic proteins in neurons, we further performed immunostaining of SNARE-complex core proteins with the neuronal marker NeuN in human PFC slices. As shown in **Figure 6C** and **6D**, the puncta intensity and area of STX1A and SNAP25 in PFC neurons (NeuN+) were markedly reduced in AD patients (Intensity, STX1A, $t_{(16)} = 4.7$, $p < 0.001$; SNAP25, $t_{(16)} = 6.0$, $p < 0.001$; Area, STX1A, $t_{(16)} = 4.9$, $p < 0.001$; SNAP25, $t_{(16)} = 4.9$, $p < 0.001$, t-test). These data have confirmed the loss of presynaptic genes in PFC neurons of AD humans.

Discussion

For Review Only

In this study, we performed transcriptomic analyses of postmortem prefrontal cortical tissue from AD and non-demented human patients (**Figure 7**). While previous studies have reported the complex AD-associated genetic alterations in different cell types (Mathys et al., 2019; Zhang et al., 2013; Zhou et al., 2020), we aimed to identify the most prominent changes in gene networks, molecular pathways and biological processes that are directly responsible for cognitive decline in AD. Using multiple bioinformatics approaches, including module co-expression analysis, MCC gene ranking in common pathways, and GSEA analysis between genomic and proteomic datasets, we have revealed the loss of SNARE complex-related genes in cortical neurons as a potential key factor causing synaptic and cognitive deficits in AD.

Our analyses of 230 human samples reliably identified two major categories of genetic changes in AD -- the downregulation of genes involved in synaptic function and the upregulation of genes involved in immune response pathways. Since the ‘early-pathology’ AD group mainly exhibits the downregulation of genes exclusively in neurons (Mathys et al., 2019), we have focused on the analyses of downregulated genes in AD that are potential targets for early intervention. The diminished gene category is most strongly associated with those involved in presynaptic vesicle docking, endocytosis, and exocytosis. In addition, genes involved in glutamatergic and GABAergic transmission are also among the top-ranking downregulated list.

Through co-expression modular analysis, we identified that the genes diminished in AD are most enriched in module 1 (M1) that contains SNARE-binding complex genes, including the core SNARE genes, *SNAP-25*, *STX1A*, and *VAMP2*. These findings were corroborated in excitatory and inhibitory neurons, when compared to a single-cell gene expression dataset from PFC of a different AD cohort (Zhou et al., 2020). Interestingly, proteomics studies of postmortem human brains in the PFC of patients with AD, Parkinson's disease (PD) with dementia, dementia with Lewy bodies and older adults without dementia also found that selected synaptic proteins were significantly lost in the various dementia groups, which was significantly correlated with the rate of cognitive decline (Bereczki et al., 2018; Bereczki et al., 2016).

In addition to M1, downregulated genes in AD are also enriched in module 8 (M8), which is involved in myosin V-mediated function. Myosin V is a calcium-activated actin-associated molecular motor protein that interacts with syntaxin-1A and is involved in vesicle transport and docking (Maschi et al., 2018; Prekeris and Terrian, 1997; Watanabe et al., 2005). Two of the hub genes in M8 central to synaptic function are *HPCA* and *RAB3A,* both encoding calcium-dependent proteins. HPCA is a calcium sensor primarily expressed in pyramidal neurons, which has been implicated in synaptic plasticity and memory processes (Burgoyne, 2007; Palmer et al., 2005; Kobayashi et al., 2005). RAB3A is a small GTP-binding protein regulating synaptic

vesicle exocytosis (Geppert et al., 1994a), and is reduced in the brain of patients with AD and other dementia (Bereczki et al., 2018). The loss of genes in M1 and M8 may directly underlie synaptic dysfunction in AD, the best correlate of cognitive impairment.

Among the large number of genes altered in AD, a key question is what gene changes are the potential causal factor for cognitive decline. A proteomic study of cognitive trajectory (Wingo et al., 2019) has revealed 344 proteins with the increased abundance in individuals with cognitive stability, many of which are involved in pre- and post-synaptic function, including presynaptic SNARE complex components, as well as postsynaptic receptors or anchoring proteins. It also revealed 225 proteins with the decreased abundance in individuals with cognitive stability, the majority of which are involved in apoptosis and inflammation, including heat-shock proteins and E3 ubiquitin ligases (Wingo et al., 2019). Employing GSEA analysis, we found that gene alterations in AD are inversely related to the higher- or lower-abundance proteins associated with cognitive stability. Synaptic vesicle genes, including *SNAP-25, STX1A, VAMP2, and STXBP1*, are on the top list of common targets involved in AD and cognitive trajectory, further suggesting that the transcriptional downregulation of synaptic genes is directly linked to cognitive decline in AD. Thus, our study has uncovered a well-defined network of genes that could be potential targets for early disease intervention.

Acknowledgement

We thank NIH NeuroBioBank for providing postmortem brain tissues from AD patients and control subjects. This work was supported by grants from the National Institutes of Health (F99NS118745 to J.B.W; R01AG064656, R01AG056060 and R21AG067597 to Z.Y).

Author contributions

J.B.W. performed bioinformatics analyses and immunoblotting experiments. He also wrote the draft. Q.C. performed qPCR and immunostaining experiments. Z.Y. supervised the project and wrote the paper.

Disclosure/ Conflict of interest

The authors declare no conflict of interests.

For Review Only

References

Antonucci, F., Corradini, I., Fossati, G., Tomasoni, R., Menna, E., and Matteoli, M. (2016). SNAP-25, a Known Presynaptic Protein with Emerging Postsynaptic Functions. Front Synaptic Neurosci *8*, 7.

Bacaj, T., Wu, D., Burre, J., Malenka, R.C., Liu, X., and Sudhof, T.C. (2015). Synaptotagmin-1 and -7 Are Redundantly Essential for Maintaining the Capacity of the Readily-Releasable Pool of Synaptic Vesicles. PLoS Biol *13*, e1002267.

Bai, B., Wang, X., Li, Y., Chen, P.-C., Yu, K., Dey, K.K., Yarbro, J.M., Han, X., Lutz, B.M., Rao, S.*, et al.* (2020). Deep Multilayer Brain Proteomics Identifies Molecular Networks in Alzheimer's Disease Progression. Neuron *105*, 975-991.e977.

Baker, K., Gordon, S.L., Melland, H., Bumbak, F., Scott, D.J., Jiang, T.J., Owen, D., Turner, B.J., Boyd, S.G., Rossi, M.*, et al.* (2018). SYT1-associated neurodevelopmental disorder: a case series. Brain *141*, 2576-2591.

Baker, R.W., and Hughson, F.M. (2016). Chaperoning SNARE assembly and disassembly. Nature Reviews Molecular Cell Biology *17*, 465-479.

Bereczki, E., Branca, R.M., Francis, P.T., Pereira, J.B., Baek, J.-H., Hortobágyi, T., Winblad, B., Ballard, C., Lehtiö, J., and Aarsland, D. (2018). Synaptic markers of cognitive decline in neurodegenerative diseases: a proteomic approach. Brain *141*, 582-595.

Bereczki, E., Francis, P.T., Howlett, D., Pereira, J.B., Höglund, K., Bogstedt, A., Cedazo-Minguez, A., Baek, J.-H., Hortobágyi, T., Attems, J.*, et al.* (2016). Synaptic proteins predict cognitive decline in Alzheimer's disease and Lewy body dementia. Alzheimer's & Dementia *12*, 1149-1158.

Burgoyne, R.D. (2007). Neuronal calcium sensor proteins: generating diversity in neuronal Ca2+ signalling. Nat Rev Neurosci *8*, 182-193.

Cardozo, L.E., Russo, P.S.T., Gomes-Correia, B., Araujo-Pereira, M., Sepúlveda-Hermosilla, G., Maracaja-Coutinho, V., and Nakaya, H.I. (2019). webCEMiTool: Co-expression Modular Analysis Made Easy. Front Genet *10*, 146.

Chapman, E.R., An, S., Barton, N., and Jahn, R. (1994). SNAP-25, a t-SNARE which binds to both syntaxin and synaptobrevin via domains that may form coiled coils. J Biol Chem *269*, 27427-27432.

Chin, C.H., Chen, S.H., Wu, H.H., Ho, C.W., Ko, M.T., and Lin, C.Y. (2014). cytoHubba: identifying hub objects and sub-networks from complex interactome. BMC Syst Biol *8 Suppl 4*, S11.

Cornell, B., and Toyo-Oka, K. (2017). 14-3-3 Proteins in Brain Development: Neurogenesis, Neuronal Migration and Neuromorphogenesis. Front Mol Neurosci *10*, 318.

De Jager, P.L., Srivastava, G., Lunnon, K., Burgess, J., Schalkwyk, L.C., Yu, L., Eaton, M.L., Keenan, B.T., Ernst, J., McCabe, C.*, et al.* (2014). Alzheimer's disease: early alterations in brain DNA methylation at ANK1, BIN1, RHBDF2 and other loci. Nature Neuroscience *17*, 1156-1163.

Fasshauer, D., Sutton, R.B., Brunger, A.T., and Jahn, R. (1998). Conserved structural features of the synaptic fusion complex: SNARE proteins reclassified as Q- and R-SNAREs. Proceedings of the National Academy of Sciences *95*, 15781-15786.

Forgrave, L.M., Ma, M., Best, J.R., and DeMarco, M.L. (2019). The diagnostic performance of neurofilament light chain in CSF and blood for Alzheimer's disease, frontotemporal

For Review Only

dementia, and amyotrophic lateral sclerosis: A systematic review and meta-analysis. Alzheimers Dement (Amst) *11*, 730-743.

Geppert, M., Bolshakov, V.Y., Siegelbaum, S.A., Takei, K., De Camilli, P., Hammer, R.E., and Sudhof, T.C. (1994a). The role of Rab3A in neurotransmitter release. Nature *369*, 493-497.

Geppert, M., Goda, Y., Hammer, R.E., Li, C., Rosahl, T.W., Stevens, C.F., and Südhof, T.C. (1994b). Synaptotagmin I: a major Ca2+ sensor for transmitter release at a central synapse. Cell *79*, 717-727.

Gjoneska, E., Pfenning, A.R., Mathys, H., Quon, G., Kundaje, A., Tsai, L.-H., and Kellis, M. (2015). Conserved epigenomic signals in mice and humans reveal immune basis of Alzheimer's disease. Nature *518*, 365-369.

Klein, H.-U., McCabe, C., Gjoneska, E., Sullivan, S.E., Kaskow, B.J., Tang, A., Smith, R.V., Xu, J., Pfenning, A.R., Bernstein, B.E.*, et al.* (2019). Epigenome-wide study uncovers large-scale changes in histone acetylation driven by tau pathology in aging and Alzheimer's human brains. Nature Neuroscience *22*, 37-46.

Kobayashi, M., Masaki, T., Hori, K., Masuo, Y., Miyamoto, M., Tsubokawa, H., Noguchi, H., Nomura, M., and Takamatsu, K. (2005). Hippocalcin-deficient mice display a defect in cAMP response element-binding protein activation associated with impaired spatial and associative memory. Neuroscience *133*, 471-484.

Lake, B.B., Ai, R., Kaeser, G.E., Salathia, N.S., Yung, Y.C., Liu, R., Wildberg, A., Gao, D., Fung, H.-L., Chen, S.*, et al.* (2016). Neuronal subtypes and diversity revealed by single-nucleus RNA sequencing of the human brain. Science *352*, 1586.

Lake, B.B., Chen, S., Sos, B.C., Fan, J., Kaeser, G.E., Yung, Y.C., Duong, T.E., Gao, D., Chun, J., Kharchenko, P.V.*, et al.* (2018). Integrative single-cell analysis of transcriptional and epigenetic states in the human adult brain. Nature Biotechnology *36*, 70-80.

Liu, Y., Zhou, Q., Tang, M., Fu, N., Shao, W., Zhang, S., Yin, Y., Zeng, R., Wang, X., Hu, G.*, et al.* (2015). Upregulation of alphaB-crystallin expression in the substantia nigra of patients with Parkinson's disease. Neurobiology of Aging *36*, 1686-1691.

Macosko, Evan Z., Basu, A., Satija, R., Nemesh, J., Shekhar, K., Goldman, M., Tirosh, I., Bialas, Allison R., Kamitaki, N., Martersteck, Emily M.*, et al.* (2015). Highly Parallel Genome-wide Expression Profiling of Individual Cells Using Nanoliter Droplets. Cell *161*, 1202-1214.

Maschi, D., Gramlich, M.W., and Klyachko, V.A. (2018). Myosin V functions as a vesicle tether at the plasma membrane to control neurotransmitter release in central synapses. Elife *7*.

Mathys, H., Davila-Velderrain, J., Peng, Z., Gao, F., Mohammadi, S., Young, J.Z., Menon, M., He, L., Abdurrob, F., Jiang, X.*, et al.* (2019). Single-cell transcriptomic analysis of Alzheimer's disease. Nature *570*, 332-337.

Mayer, M.P., and Bukau, B. (2005). Hsp70 chaperones: Cellular functions and molecular mechanism. Cellular and Molecular Life Sciences *62*, 670.

Miller, J.A., Woltjer, R.L., Goodenbour, J.M., Horvath, S., and Geschwind, D.H. (2013). Genes and pathways underlying regional and cell type changes in Alzheimer's disease. Genome Medicine *5*, 48.

Mostafavi, S., Gaiteri, C., Sullivan, S.E., White, C.C., Tasaki, S., Xu, J., Taga, M., Klein, H.-U., Patrick, E., Komashko, V.*, et al.* (2018). A molecular network of the aging human brain provides insights into the pathology and cognitive decline of Alzheimer's disease. Nature Neuroscience *21*, 811-819.

For Review Only

Nativio, R., Donahue, G., Berson, A., Lan, Y., Amlie-Wolf, A., Tuzer, F., Toledo, J.B., Gosai, S.J., Gregory, B.D., Torres, C.*, et al.* (2018). Dysregulation of the epigenetic landscape of normal aging in Alzheimer's disease. Nature Neuroscience *21*, 497-505.

Nativio, R., Lan, Y., Donahue, G., Sidoli, S., Berson, A., Srinivasan, A.R., Shcherbakova, O., Amlie-Wolf, A., Nie, J., Cui, X.*, et al.* (2020). An integrated multi-omics approach identifies epigenetic alterations associated with Alzheimer's disease. Nature Genetics *52*, 1024-1035.

Palmer, C.L., Lim, W., Hastie, P.G., Toward, M., Korolchuk, V.I., Burbidge, S.A., Banting, G., Collingridge, G.L., Isaac, J.T., and Henley, J.M. (2005). Hippocalcin functions as a calcium sensor in hippocampal LTD. Neuron *47*, 487-494.

Prekeris, R., and Terrian, D.M. (1997). Brain myosin V is a synaptic vesicle-associated motor protein: evidence for a Ca2+-dependent interaction with the synaptobrevin-synaptophysin complex. J Cell Biol *137*, 1589-1601.

Raj, T., Li, Y.I., Wong, G., Humphrey, J., Wang, M., Ramdhani, S., Wang, Y.-C., Ng, B., Gupta, I., Haroutunian, V.*, et al.* (2018). Integrative transcriptome analyses of the aging brain implicate altered splicing in Alzheimer's disease susceptibility. Nature Genetics *50*, 1584-1592.

Rosenzweig, R., Nillegoda, N.B., Mayer, M.P., and Bukau, B. (2019). The Hsp70 chaperone network. Nature Reviews Molecular Cell Biology *20*, 665-680.

Shannon, P., Markiel, A., Ozier, O., Baliga, N.S., Wang, J.T., Ramage, D., Amin, N., Schwikowski, B., and Ideker, T. (2003). Cytoscape: a software environment for integrated models of biomolecular interaction networks. Genome Res *13*, 2498-2504.

Südhof, T.C., and Rothman, J.E. (2009). Membrane Fusion: Grappling with SNARE and SM Proteins. Science *323*, 474-477.

Szklarczyk, D., Gable, A.L., Lyon, D., Junge, A., Wyder, S., Huerta-Cepas, J., Simonovic, M., Doncheva, N.T., Morris, J.H., Bork, P.*, et al.* (2019). STRING v11: protein-protein association networks with increased coverage, supporting functional discovery in genome-wide experimental datasets. Nucleic Acids Res *47*, D607-D613.

Verderio, C., Pozzi, D., Pravettoni, E., Inverardi, F., Schenk, U., Coco, S., Proux-Gillardeaux, V., Galli, T., Rossetto, O., Frassoni, C.*, et al.* (2004). SNAP-25 modulation of calcium dynamics underlies differences in GABAergic and glutamatergic responsiveness to depolarization. Neuron *41*, 599-610.

Wang, L., Colodner, K.J., and Feany, M.B. (2011). Protein misfolding and oxidative stress promote glial-mediated neurodegeneration in an Alexander disease model. J Neurosci *31*, 2868-2877.

Watanabe, M., Nomura, K., Ohyama, A., Ishikawa, R., Komiya, Y., Hosaka, K., Yamauchi, E., Taniguchi, H., Sasakawa, N., Kumakura, K.*, et al.* (2005). Myosin-Va regulates exocytosis through the submicromolar Ca2+-dependent binding of syntaxin-1A. Mol Biol Cell *16*, 4519-4530.

Wingo, A.P., Dammer, E.B., Breen, M.S., Logsdon, B.A., Duong, D.M., Troncosco, J.C., Thambisetty, M., Beach, T.G., Serrano, G.E., Reiman, E.M.*, et al.* (2019). Large-scale proteomic analysis of human brain identifies proteins associated with cognitive trajectory in advanced age. Nature Communications *10*, 1619.

Wu, D., Bacaj, T., Morishita, W., Goswami, D., Arendt, K.L., Xu, W., Chen, L., Malenka, R.C., and Sudhof, T.C. (2017). Postsynaptic synaptotagmins mediate AMPA receptor exocytosis during LTP. Nature *544*, 316-321.

For Review Only

Zhang, B., Gaiteri, C., Bodea, L.G., Wang, Z., McElwee, J., Podtelezhnikov, A.A., Zhang, C., Xie, T., Tran, L., Dobrin, R., *et al.* (2013). Integrated systems approach identifies genetic nodes and networks in late-onset Alzheimer's disease. Cell *153*, 707-720.

Zhang, J., and Zhou, Y. (2018). 14-3-3 Proteins in Glutamatergic Synapses. Neural Plast *2018*, 8407609.

Zhong, S., Zhang, S., Fan, X., Wu, Q., Yan, L., Dong, J., Zhang, H., Li, L., Sun, L., Pan, N., *et al.* (2018). A single-cell RNA-seq survey of the developmental landscape of the human prefrontal cortex. Nature *555*, 524-528.

Zhou, Y., Song, W.M., Andhey, P.S., Swain, A., Levy, T., Miller, K.R., Poliani, P.L., Cominelli, M., Grover, S., Gilfillan, S., *et al.* (2020). Human and mouse single-nucleus transcriptomics reveal TREM2-dependent and TREM2-independent cellular responses in Alzheimer's disease. Nat Med *26*, 131-142.

Zhou, Y., Zhou, B., Pache, L., Chang, M., Khodabakhshi, A.H., Tanaseichuk, O., Benner, C., and Chanda, S.K. (2019). Metascape provides a biologist-oriented resource for the analysis of systems-level datasets. Nat Commun *10*, 1523.

For Review Only

Figure legends

Figure 1. Differential gene expression analysis of AD patients.

A, B, GO analysis of molecular functions associated with downregulated (A) or upregulated DEGs (B) in PFC of AD samples. **C,** Sunburst plot representing cellular component enrichment analysis of downregulated synaptic genes in PFC of AD samples. Higher red intensities are associated with more significant enrichments. All the identified synaptic genes are represented in the red circle at the center of the plot. Presynaptic genes are the most overrepresented synaptic subcluster. **D,** Synaptic cellular component analysis representing gene abundance and enrichment in each synaptic cluster. **E, F,** Interaction networks of downregulated synaptic hub genes in AD samples.

Figure 2. Transcriptomic data show the loss of synaptic genes in PFC of AD patients.

A, C, Heatmaps of synaptic gene expression values in postmortem PFC from 101 control humans and 129 AD patient samples. The genes encode SNARE-complex components; glutamate receptors, transporters, anchoring proteins; GABA receptors, transporters or synthesizing enzymes. **B, D,** Box plots showing the expression of selected genes (highlighted in blue) in control vs. AD humans. *** $p<0.001$, t-test.

Figure 3. Modular analysis of AD gene expression data.

A, Gene co-expression modules for AD DEGs plotted by abundance. **B,** Plot of mean eigengene expression values representing summary gene expression within each module for AD and control groups. **C,** Volcano plot displaying Module 1 DEGs. **D,** GO molecular function enrichment analysis of Module 1 AD DEGs. **E,** Clustergram matrix of Module 1 top-ranking DEGs and molecular function pathways. SNARE-binding genes are highlighted in blue. **F,** Network analysis showing the interaction of downregulated SNARE-binding genes in AD.

Figure 4. Comparison of bulk and single neuron-specific genomic alterations of AD.

A, Venn diagram representing significant DEGs from bulk PFC or single excitatory and inhibitory PFC neurons. **B,** Circos plot of all three DEG lists from bulk PFC or single excitatory (Ex.) or inhibitory (In.) neurons, where connecting blue lines represent genes within the same enriched ontology pathway, and purple lines represent linked identical genes between groups. **C,** GO enrichment analysis of common biological pathways from bulk PFC or single Ex. or In. neurons. **D,** Heatmaps of the common DEGs from bulk PFC or single Ex. or In. neurons. Synaptic genes

For Review Only

are highlighted in blue. **E,** Bar graphs representing FC values of hub genes from the common DEGs. **F,** Interaction network of hub genes from the common DEGs.

Figure 5. Correlation between cognitive stability proteins and AD-altered genes.

A, Bar graph showing the classification of cognitive stability (CS) proteins (569 total, 344 higher-abundance in CS, 225 lower-abundance in CS). **B,** Bar graph displaying bi-directional effect size in key differentially expressed CS proteins in humans with cognitive stability, compared to patients with cognitive decline. Proteins with positive or negative effect sizes represent those higher-abundance or lower-abundance CS proteins, respectively. **C, D,** GSEA plots of ranked AD DEGs, compared to higher-abundance (C) or lower-abundance (D) CS proteins. **E,** Venn diagram representing Module 1 AD DEGs (522), CS proteins (569), and downregulated genes in AD (1241). **F,** Heatmap of fold-change (FC) values for the 20 common targets shown in (E). Synaptic vesicle genes are highlighted in blue.

Figure 6. Confirmation of the loss of synaptic genes in PFC of AD humans.

A, Bar graphs showing qPCR data of the selected synaptic genes in PFC (BA10) of postmortem tissues from AD patients vs. control (ctrl) subjects (n = 6 pairs). **B,** Representative Western blots and quantification of synaptic proteins (STX1A, SNPA25, VAMP2 and PSD95) in the synaptic fraction of PFC from AD patients vs. control subjects (Ctrl: n = 5, AD: n = 6). **C,** Representative immunofluorescence images of synaptic proteins (STX1A, SNAP25, VAMP2), co-stained with the neuronal marker NeuN, in PFC from AD patients vs. control subjects. scale bar, 20 μm. **D,** Quantification of the fluorescence average intensity and puncta area of synaptic proteins in AD and controls (n = 9 slices from 3 humans each group). In all figures, *: $p < 0.05$, **: $p < 0.01$, ***: $p < 0.001$, *t*-test.

Figure 7. Graphical schematic of study design and major findings. **1,** Analysis of RNA-microarray data from PFC of AD patients (129) and healthy controls (101) identifies the loss of genes in synaptic function and the increased expression of genes in immune/stress response. **2,** Co-expression modular analysis links synaptic dysregulation in AD to downregulated genes that encode constituents of SNARE-binding complex. **3,** Analysis of single-cell RNA-seq data identifies that SNARE-binding elements are consistently downregulated in cortical neurons from AD patients. **4,** Cross comparison of AD transcriptomic and cognitive trajectory proteomic datasets highlights core SNARE elements as primary factors for AD-related cognitive decline.

https://mc.manuscriptcentral.com/braincom

For Review Only

Fig. 1

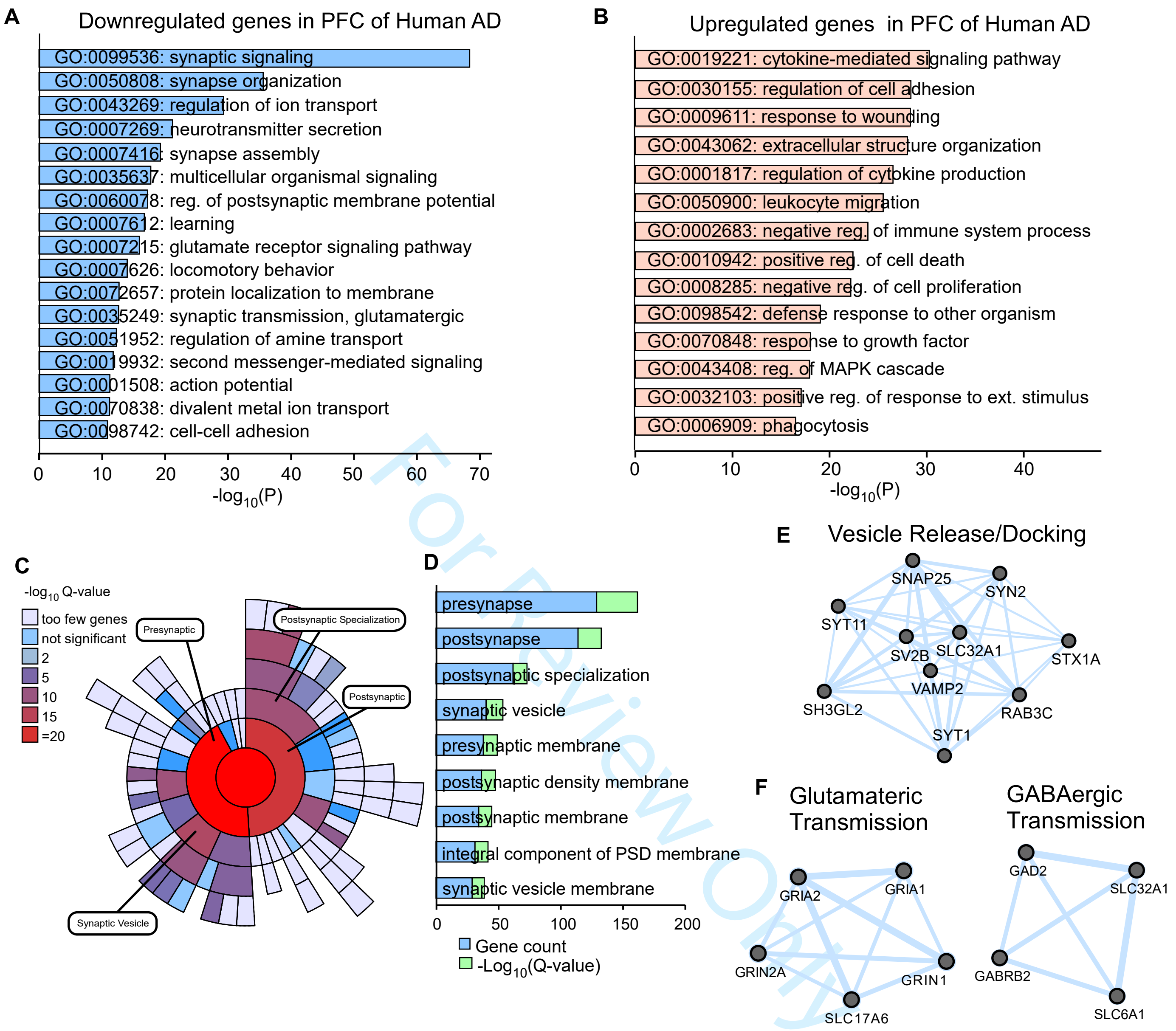

For Review Only

Fig. 2

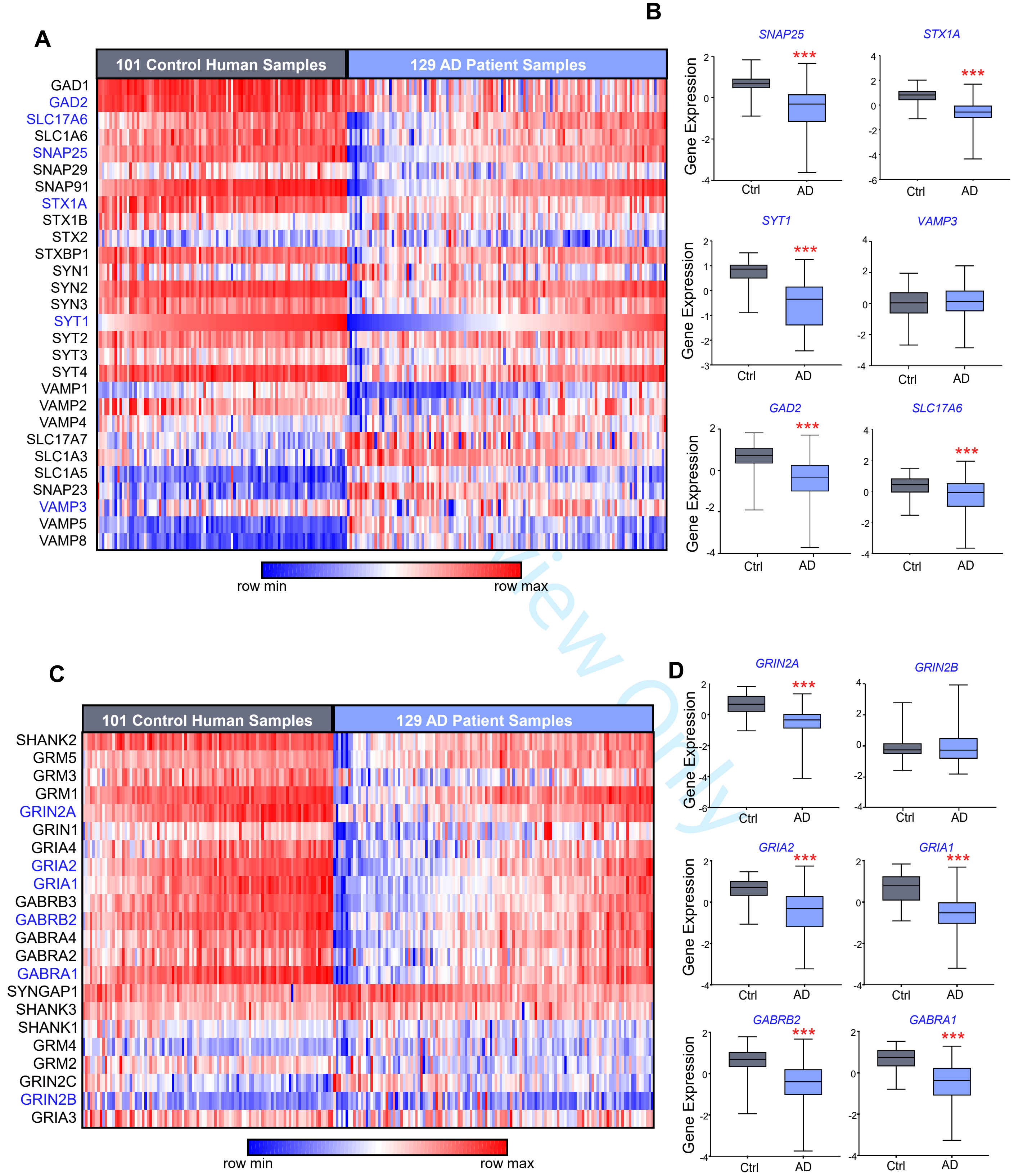

Review Only

Fig. 3

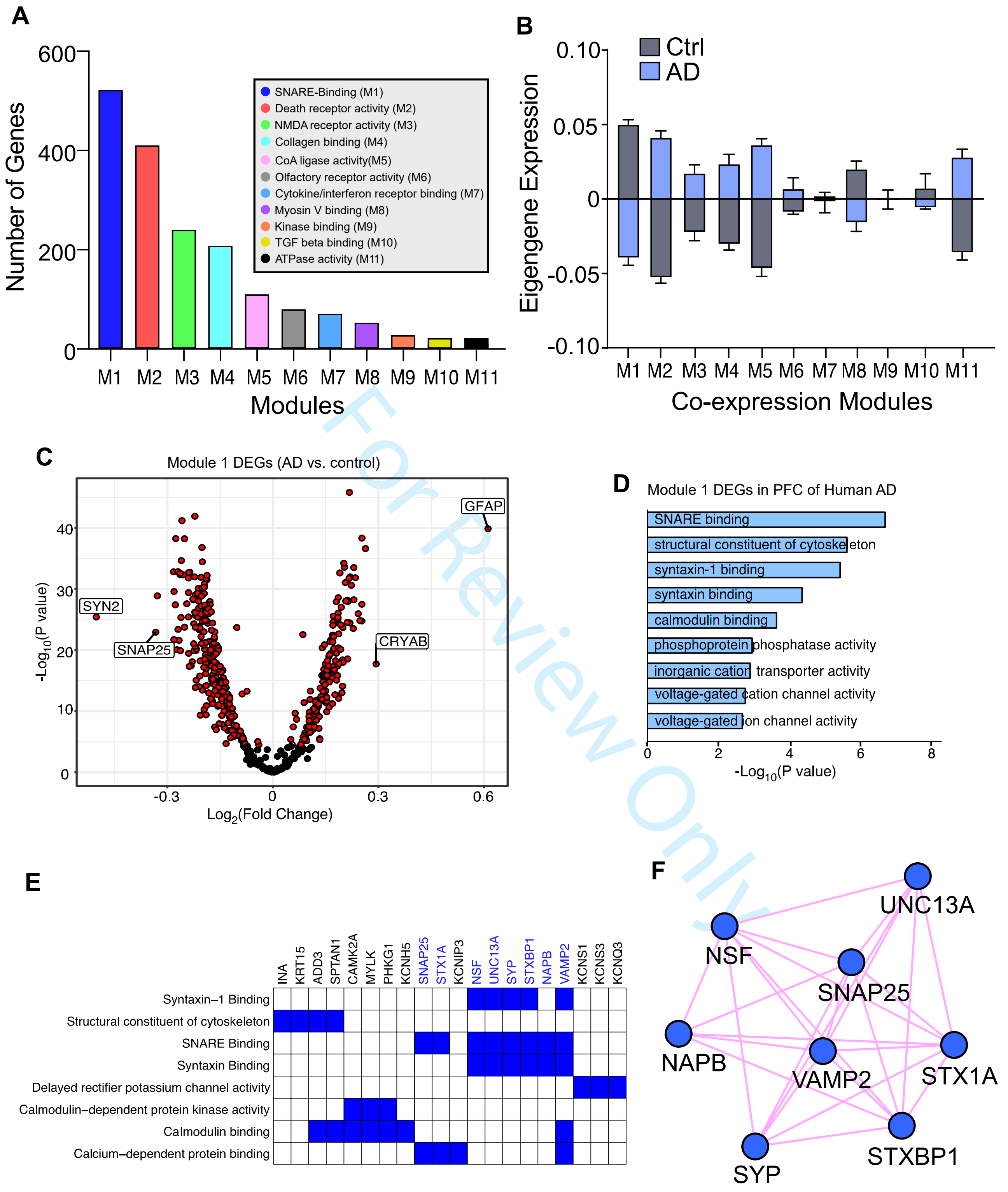

For Review Only

Fig. 4

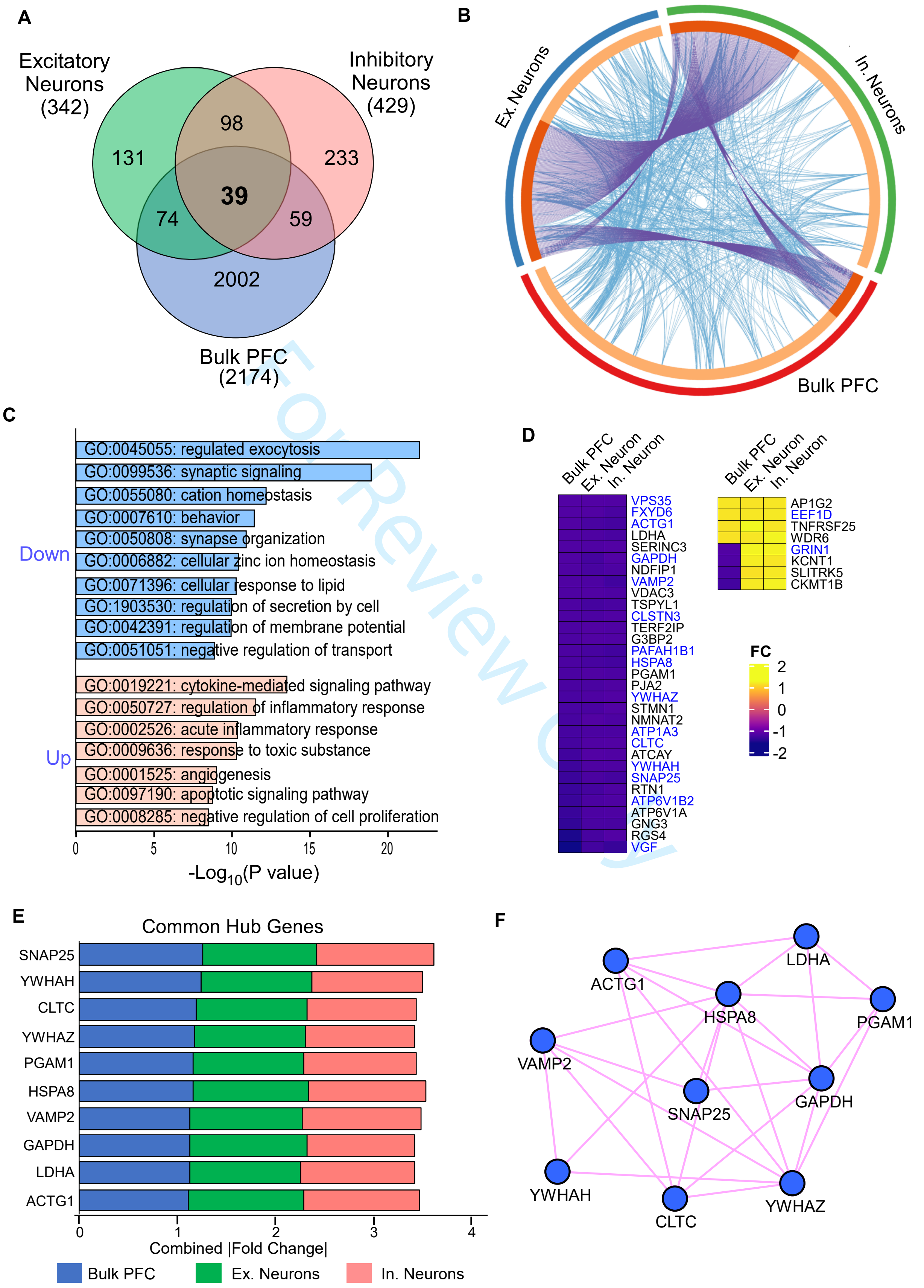

For Review Only

Fig. 5

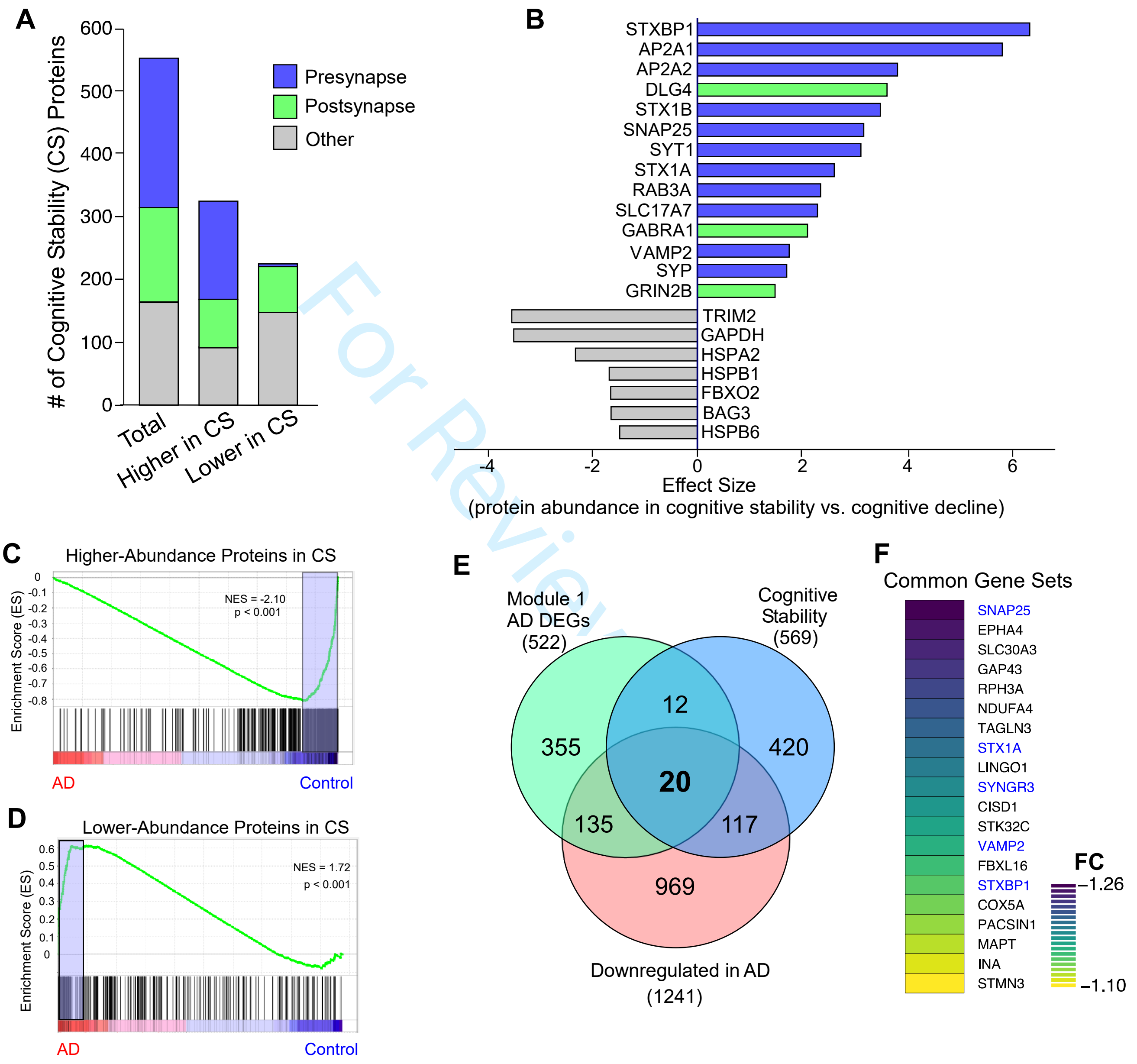

For Review

Fig. 6

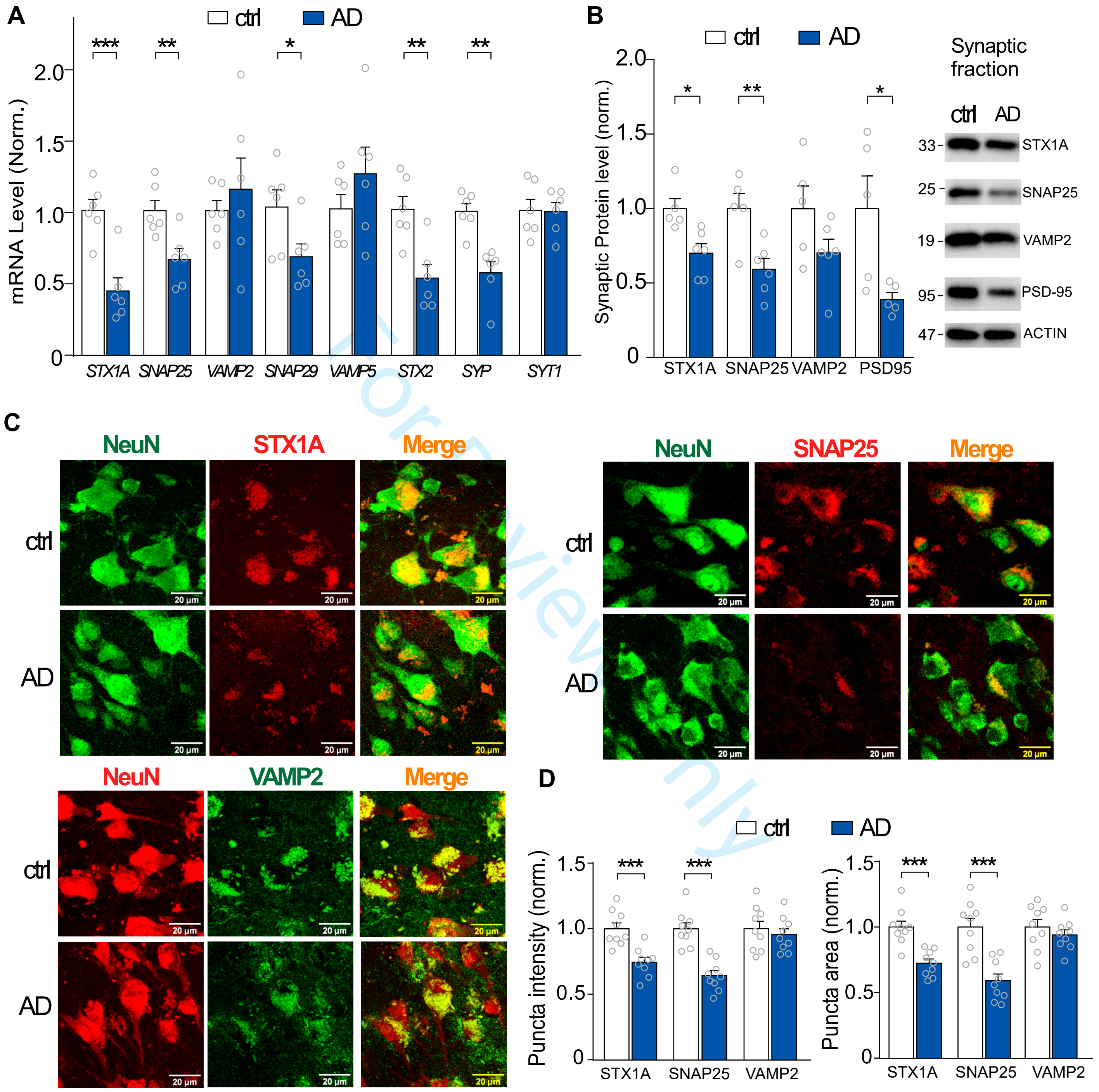
A
ctrl
AD
mRNA Level (Norm.)
2.0
1.5
1.0
0.5
0
***
**
*
**
**
STX1A
SNAP25
VAMP2
SNAP29
VAMP5
STX2
SYP
SYT1
B
ctrl
AD
Synaptic Protein level (norm.)
2.0
1.5
1.0
0.5
0
*
**
*
STX1A
SNAP25
VAMP2
PSD95
Synaptic fraction
ctrl
AD
33-
STX1A
25-
SNAP25
19-
VAMP2
95-
PSD-95
47-
ACTIN
C
NeuN
STX1A
Merge
ctrl
AD
NeuN
SNAP25
Merge
ctrl
AD
NeuN
VAMP2
Merge
ctrl
AD
20 µm
D
ctrl
AD
Puncta intensity (norm.)
1.5
1.0
0.5
0
***
***
STX1A
SNAP25
VAMP2
Puncta area (norm.)
1.5
1.0
0.5
0
***
***
STX1A
SNAP25
VAMP2

Fig. 7

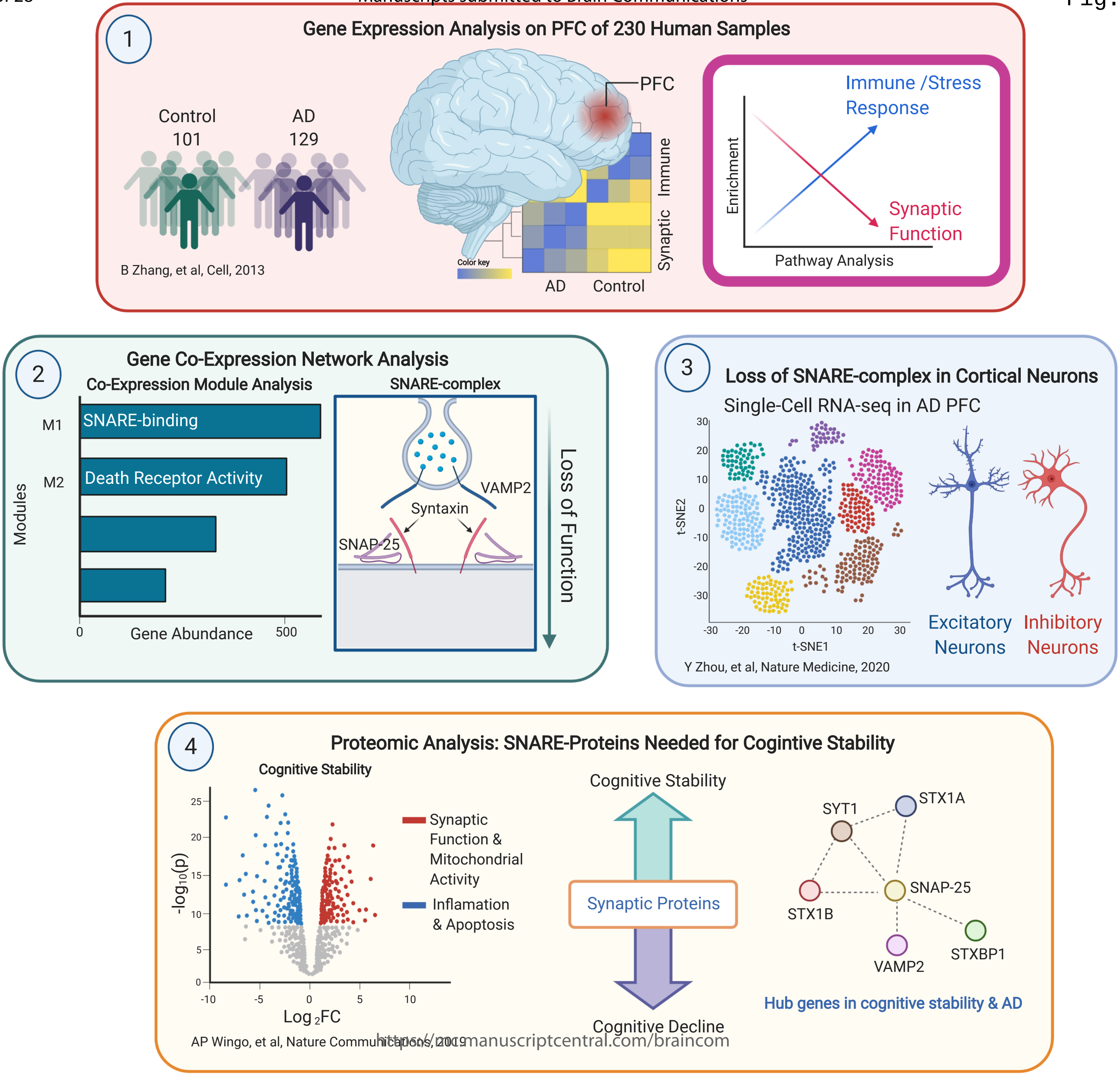

https://mc.manuscriptcentral.com/braincom

Table 1. Summary of modules containing co-expressed genes that represent dysregulated molecular functions in PFC of AD patients.

Module	Number of Genes	Molecular Function	Key Constituents
M1	522	SNARE-Binding	*SNAP25, STX1A, STXBP1, SYT1A, VAMP2*
M2	410	death receptor activity	*TNFRSF1A, TCIRG1, PXN, LRP10, RAB13*
M3	240	NMDA glutamate receptor activity	*AHDC1, PCDHGC5, PCDHGB3, PCDHGB5, PCDHGA1*
M4	208	collagen binding	*FAM20A, PCOLCE, FOXC1, AOX1, OLFML2A*
M5	110	coA ligase activity	*SOX9, EDG1, EMX2, SLC1A3, ACSBG1*
M6	80	olfactory receptor activity	*IGSF2, KRTAP20-1, RBMY2FP, EVC, CYP7B1*
M7	71	cytokine/interferon receptor binding	*PRAMEF10, OR4K17, CLDN18, RP11-297H3.4, LOC728676*
M8	53	myosin V binding	*RAB3A, HPCA, C12orf53, MAPRE3, LOC442211*
M9	28	kinase binding	*EFS, PPP1R14A, C11orf9, DAAM2, RASGRP3*
M10	22	TGF beta binding	*CHST13, LOC374973, RNF126P1, CABC1, OR7G3*
M11	22	ATPase activity	*ABCA2, SOX10, MYO9B, PCTK3, PLXNB3*

For Review Only

Genes were organized into modules based on expression similarity using Pearson correlation and the Dynamic Tree Cut package in webCEMiTool, with a minimum of 20 genes per module. Molecular function for each module was assigned based on the top functional group. Key constituents are hub or top-ranking genes in each module. Complete gene list for each module can be found in Sup. Table 2.
