# Supplementary material for: Transcriptomic analysis of human brains with Alzheimer’s disease reveals the altered expression of synaptic genes linked to cognitive deficits
Source: Brain Commun. 2021 Jun 3;3(3):fcab123. doi: 10.1093/braincomms/fcab123 (PMC8374979; doi:10.1093/braincomms/fcab123)
Supplement: fcab123_Supplementary_Data [file Revision 2-Decision Letter.pdf › Revision 2-Decision Letter.pdf]

Revision 2-Decision Letter

22-Apr-2021

Dear Dr. Yan,

It is a pleasure to accept your manuscript entitled "Transcriptomic Analysis of Human Brains with Alzheimer's Disease Reveals the Altered Expression of Synaptic Genes Linked to Cognitive Deficits" for publication in Brain Communications. Any comments made by the reviewer(s) are included below.

The next step is for your paper to go through "First Look". We will contact you again shortly if any final files or formatting changes are required for publication or if we have any queries.

If no changes are needed, your submission will be transferred to our Production Department and you will hear from the Production Editor in due course.

Video abstracts - Brain Communications encourages authors to provide a video abstract to accompany their article. These are hosted on the Brain/Brain Communications YouTube channel. If you are interested in producing a 5-min video abstract to promote your article, then please contact the Editorial Office (brcoms.editorialoffice@oup.com) for further details and guidelines.

Thank you very much for your contribution. On behalf of the Editors of Brain Communications, we look forward to your future contributions to the Journal.

Yours sincerely,

Professor Tara Spires-Jones
Founding Editor, Brain Communications

Reviewer(s)' Comments to Author:

Reviewer: 1 (Becky Carlyle)

Comments to the Author
Many thanks to the authors for making the majority of the changes as requested, I think the thought process of the paper is much clearer, and the clustered heatmaps look extremely persuasive. The immunofluorescence in Figure 6 is much clearer at this level of magnification.

I would ask that for future publications the authors revisit the western blotting guidelines I sent in previous comments from JBC, particularly the switch from a single protein loading control to a total protein stain, as the blots shown do not meet their stringent publication standards. I think the multi-modal layers of evidence are sufficient for publication here, but those blots do not stand alone as strong data.

Reviewer: 2

Comments to the Author
My thanks to the authors - my concerns have been addressed in this revision.
