# Supplementary material for: Transcriptomic analysis of human brains with Alzheimer’s disease reveals the altered expression of synaptic genes linked to cognitive deficits
Source: Brain Commun. 2021 Jun 3;3(3):fcab123. doi: 10.1093/braincomms/fcab123 (PMC8374979; doi:10.1093/braincomms/fcab123)
Supplement: fcab123_Supplementary_Data [file Original Submission - Authors Response.pdf › Original Submission - Authors Response.pdf]

Reviewer: 1

Comments to the Author
In this manuscript, Williams et al. use a series of bioinformatic tools in an analysis of older microarray data, to highlight the role that synaptic transcripts and protein play in Alzheimer's Disease pathogenesis. The outcome of this analysis is then compared with a single cell results set and a proteomic results set. This work uses a substantial number of tools and approaches, some of which I am not familiar with, but all the approaches return to the central theme that synapses are critical to Alzheimer's disease pathogenesis.
A:
We appreciate Reviewer 1's positive comments and helpful suggestions.

Major issues
1) My biggest issue with the manuscript is that is it unclear where the previous analysis of this dataset ends and this new analysis begins. In looking at the Cell 2013 paper, it seems that they used linear modeling that took into account age, PMI, sex etc in performing differential expression analysis. The analysis in this paper uses only a subset of the samples from the original paper, and I am uncertain whether the p values used in this re-analysis came from the 2013 paper, or whether a new differential expression analysis was performed. This needs clarifying more strongly in both the methods and the results.
A:
In the 2013 Cell paper by Zhang, Bin, et al., the authors used a gene-network modular analysis approach to classify pathways that describe functional changes in prefrontal cortex (PFC) of AD patients. This network approach highlights functional changes in several cell types and their associated processes. The authors directed their efforts on modules associated with immune response related to changes in microglia expressed genes (primarily TYROBP). Our current study used bioinformatic and statistical methods to focus on changes in synaptic and neuronal function in AD, not elucidated by the 2013 study. Our methods produced p values in conjunction with our own analysis. In doing so, we used a subseries (GSE44770) of their SuperSeries (GSE44772) data that was deposited in GEO. This subseries includes 230 samples specifically from PFC, while the SuperSeries also includes samples from cerebellum and visual cortex. Tissue sample assessment and normalization of several factors, including PMI, pH, and RNA integrity, were conducted by the 2013 study after acquisition from the Harvard Brain Tissue Resource Center, therefore our analysis took advantage of these efforts. However, we further analyzed this dataset using NCBI's GEO2R calculator (within GEO) to calculate and examine the value distribution for all 230 samples. The data displayed a median-centered distribution, which suggests that the dataset is normalized and cross-comparable.

2) Regardless of how the p-values were computed, it does not appear that they were corrected for multiple testing. If they were, and this is an error on my part, it should be very clearly stated in the paper in both the methods and the results. The original microarray included probes for just under 40,000 genes, and around 2000 of them were found to have a p value of 0.05 or below. This is pretty much exactly the number of genes you would expect to find by chance. pValue correction should absolutely be performed before continuing to take these genes forwards to ontology analysis.
A:
The original dataset was acquired under the GEO accession number GSE44770, in which expression values for 39,280 genes were included. Because many microarray studies contain multiple probes for the same genes, there are often replicates. We adjusted this issue by selecting one replicate (with the

maximal median expression value) for each gene, which reduced the dataset to 19,527 genes. We then used the Limma Voom package to assess differential gene expression in this dataset and produced our own analysis of the data. Using a p value cutoff of less than or equal to 0.05, we obtained 10,344 significant DEGs that were then further restricted by a fold change (FC) cutoff. Concerning the reviewer's inquiry, we also calculated the adjusted p values. Using an adj. p value of less than or equal 0.05, there were 10,057 significant DEGs. When using p (≤ 0.05) or adj p values (≤ 0.05), as well as a FC cutoff of ± 10%, either set of data gave exactly the same 2,174 significant DEGs. So these genes were used in further analyses in this study. The manuscript has been updated using adj. p <0.05.

3) For the network diagrams showing hub proteins (Figure 1 E & F) can these hubs be shown on a network background of the non-hub DEG genes in those ontological categories? These particular plots don't show much, other than the hub proteins are connected to each other. It would be more informative to see that they were, in fact, each connected to a number if other DEGs, increasing confidence in the hub analysis.

A:

It is a great point to present a more holistic view of the relationship of significant hub genes. The synaptic gene set contains 240 genes. Using the Mathews correlations coefficient (MCC), a common algorithm for parsing out hub genes (genes that are high ranking in a network based correlative assessment), we identified hub genes that are highly connected to the entire gene population. We have unbiasedly selected the top 50 hub genes to demonstrate a more informative gene network (see **revised Figure 1E**).

4) I'm not used to seeing heatmaps like those in Figure 2A and C. I think I find the lack of clustering jarring – why was the decision made to not use clustering, at least for the rows? I am unclear as to exactly how the data are scaled – are they scaled and centered, or is some kind of ranking being used? Or is there no scaling? Please make this clearer. It would also be interesting to see how well AD clusters from Normal, rather than separating them as has been done here.

A:

These heatmaps are indeed row clustered via the k-means algorithm, which is the standard for row clustering. Here we used a cluster factor of 2 that reliably separates gene expression values by high or low expression levels in their respective groups. This information has been updated in the methods and figure 2 legend. We also performed hierarchical clustering on columns, however considering the large amount of samples used here, we feel that such a representation would be better suited for representing the entire genome. The purpose of this figure is to demonstrate the expression differences in key synaptic genes between AD vs health control patients, so row clustering is more relevant.

5) The in-house confirmation experiments in Figure 6 are poorly described and need more details.

a. What are the demographics of the samples? Were they well matched for age, which is another determinant of synapse density?

b. What method of microscopy was used for the imaging? What antibodies?

c. What do the error bars on 6A show? SEM?

d. All lanes of the western blots should be shown, if there are only 11 samples total

e. The imaging focuses on the cell bodies of the neurons, and NeuN is primarily a nuclear marker for neurons. See Figure 1E and 1F here for someone who has attempted something similar using HRP methods: https://pubmed.ncbi.nlm.nih.gov/22554416/#&gid=article-figures&pid=figure-1-uid-0

A:

a) The demographics information of our detected human samples (age and sex) used in Figure 6 has been included in Supplementary Fig. 2.

b) The microscope used, a more detailed explanation of the image analysis, and antibody information has been added to the *immunohistochemistry methods* section.
c) SEM was used for all statistical analysis. We have added this information to the Figure 6 legend.
d) Full uncropped Western blots of all detected samples have been added as Supplementary Fig. 2.
e) NeuN was used here as a proxy for visualizing IHC results exclusively in neurons.

Minor issues

1) The methods mention GADPH as a western blot antibody – there is no band shown for GADPH on the figure, but there is a band for ACTIN. Which was used? For future reference, it is no longer considered best practice to normalize western blots to a single reference protein (and I have seen that GAPDH commonly changes in AD vs control), a total protein stain should be used instead. See the blot section here: http://jbcresources.asbmb.org/collecting-and-presenting-data

A:

We apologize for the error in methods. It should be ACTIN, not GADPH. It has been corrected.

2) Spelling correction needed on 1F title

A:

The spelling has been corrected.

3) Was there information on cognitive status of the cases used for the original study, or were they all neuropathology confirmed only? Given up to one thirds of community dwelling individuals may have AD-level pathology but no evidence of cognitive impairment, this is important.

A:

According to 2013 Cell paper by Zhang, Bin, et al., "All autopsied brains were collected from subjects with LOAD diagnosis or from normal nondemented subjects, for whom both the donor and the next of kin had completed the HBTRC informed consent (http://www.brainbank.mclean.org/).". However, a composite cognitive score was not able to be retrieved for these samples.

4) For figure 3B, is it possible to plot a single enrichment in AD value for each module rather that the bi-directional plots? It's very difficult to visually compare the two blots and assess the enrichment.

A:

Fig. 3B is from eigengene expression values assigned to every sample in every module. This "summary score" is a representative value of the correlation between the sample, module and respective genes. The bi-directional plots are the mean of that score across the entire group (AD or control). Therefore, if the AD bar is above zero, then that module is positively correlated with AD, and if the AD bar is below zero, then that module is negatively correlated with AD, and we have added such explanations. We are unable to find a way to represent this information as a single value.

5) The process of Figure 3 currently seems a bit circular. I'm not familiar with the webCEMiTool, but it looks like it probably identified covarying genes/proteins, places them into modules, then look for over-representation of GO terms in these modules – this is your Figure 3A. So then when you take proteins from M1 and do your own GO analysis to find the same GO category at the top, this seems a little circular. Perhaps you could make it a little clearer what Figure 3D adds to the analysis?

A:

The reviewer is correct here in how genes are assigned to particular modules. We assigned the functional role of each module, then deposited the genes in each module into the ontology database EnrichR and assigned the top GO category as the major functional group of that module (Fig. 3A). Because Module 1 is the main module negatively correlated with AD, we further explored more of the

top functional groups for Module 1 (Fig. 3D). From there we further investigated how the top (most significant) genes in Module 1 fit into these GO categories (Fig. 3E), where we can see how the categories and respective genes are similar or distinct.

6) It would be great to have a bigger version of plot 4B (Circos plot) in the supplement with the gene names on the exterior of the circle – I don't find this unlabelled version to be particularly informative.

A:

It is difficult to assign the gene names on the exterior for this particular type of circus plot, because we are taking three distinct gene sets and showing a visualization of how well they intersect with each other. In a recent paper of our group where there is a single genome being represented, we were able to produce such a plot (Cao Q et al., 2020, Science Advances, 6(50), eabc8096. Fig. 5C). In Fig. 4D, we have listed the identified overlapping genes between the three groups.

7) Figure 4E as a stacked plot is very difficult to interpret – would be better unstacked. Stacking works well in 5A where you're talking about a proportion of a whole, less well in comparing fold changes across different cell types.

A:

As suggested, we have **revised Figure 4E** to make the bar graph unstacked and more clearly display fold change differences.

8) Not a note for this paper but more general advice: I would advise the author to start moving away from using web tools and to hard code their analyses in their preferred coding language. Code can then be provided through github, bitbucket or similar. Many of these tools have associated R packages and integration, for example. This would help with reproducibility and access to the data for higher-impact papers in their future, and also helps them build analytical pathways in house that can be accessed repeatedly much more easily than using web tools.

A:

We appreciate the reviewer's valuable comments here and will take heed to such advice. This is really a great point.

Reviewer: 2

Comments to the Author

In this manuscript, the authors conduct an integrative analysis of microarray, single nucleus RNA-seq, and proteomics data sets, to study the role of aberrant gene expression in AD and its relation to cognitive trajectory. My comments will mainly pertain to the bioinformatics analyses performed, terminology used, and the figures; I feel that some improvements can be made to clarify these.

I would also make the overall comment that I would be careful to ensure that the strength of statements and central claims made (for example, that the key determinant for cognitive deficits in Alzheimer's has been revealed) are fully supported and justified by the analyses performed.

A:

We appreciate Reviewer 2's meticulous evaluation and valuable comments.

Specific comments:

page 8, "Analyses of the transcriptomic data with a cutoff of p < 0.05 and fold change (FC) of 10% identified 2174 differentially expressed genes (DEGs) (1241 down; 933 up). "

Is the p-value cut-off of < 0.05 mentioned here a raw p-value, or a multiple-testing corrected "adjusted" p-value? If it is an adjusted p-value, this should be explicitly mentioned, and if not, it should be mentioned why a multiple-testing corrected p-value threshold was not used (since this would be standard practice). It would also be a valuable resource to include the full differential expression results (p-values, adjusted p-values, fold changes) as a Supplemental Table.

A:

The original dataset was acquired under the GEO accession number GSE44770, in which expression values for 39,280 genes were included. Because many microarray studies contain multiple probes for the same genes, there are often replicates. We adjusted this issue by selecting one replicate (with the maximal median expression value) for each gene, which reduced the dataset to 19,527 genes. We then used the Limma Voom package to assess differential gene expression in this dataset and produced our own analysis of the data. Using a p value cutoff of less than or equal to 0.05, we obtained 10,344 significant DEGs that were then further restricted by a fold change (FC) cutoff. Concerning the reviewer's inquiry, we also calculated the adjusted p values. Using an adj. p value of less than or equal 0.05, there were 10,057 significant DEGs. When using p (≤ 0.05) or adj p values (≤ 0.05), as well as a FC cutoff of ± 10%, either set of data gave exactly the same 2,174 significant DEGs. So these genes were used in further analyses in this study. The manuscript has been updated using adj. p <0.05.

page 8, "GO enrichment analyses of these significant DEGs indicated that the most prominently down-regulated genes in AD were …. (Figure 1A). … (Figure 1B)"

"Prominently" here might suggest that the genes annotated with these GO terms are the genes with the smallest p-values or largest fold changes, but that is not necessarily the case. I think it might be better to simply say that these GO terms were the most significantly enriched (unless a statement about p-values or fold changes *is* being made).

A:

We have revised it by saying that these GO terms were the most significantly enriched.

page 8, "With synaptic signaling being the most significantly downregulated GO category, we further explored dysregulated synaptic pathways in AD. …. (Figure 1C and 1D). … (Figure 1C, -Log10 p-value = 14.37)."

It would be good to state what background gene set was used for the synaptic enrichment analysis (e.g. brain expressed genes vs only synaptic genes) as this makes a difference to interpretation of the enrichment p-values obtained.

A:

We have added the statement about what background gene set was used for the synaptic enrichment analysis.

page 8, "Hub co-expression analyses were performed on the 41 downregulated synaptic vesicle-clustered genes (Figure 1E), … with SNAP-25 as the top-ranking node."

It would be great to state more explicitly, particularly for the benefit of non-bioinformatician readers, what hub co-expression analysis constitutes. I think from the Methods section, that this involves using

the STRING database to construct an interaction network of the 41 down-regulated synaptic vesicle-clustered genes, and then using MCC to identify hub genes in this network? But I'm not clear what role co-expression plays in this?
A:
Figures 1E and 1F have been consolidated to highlight broader interactions of downregulated synaptic genes in AD (see **revised Fig. 1E**), as suggested by reviewer 1. In addition, we have revised the manuscript to more clearly explain what hub genes are and the relevance in our studies.

page 9, "Hub co- expression analyses also revealed the downregulation of synaptic genes encoding receptors, transporters and enzymes mediating glutamatergic transmission (e.g. GRIA1, GRIA2, GRIN2A, and SLC17A6) or GABAergic transmission (e.g. GAD2, GABRB2, and SLC6A1) in AD (Figure 1F)."

I'm unsure as to which sets of genes formed input for the hub analyses that produced the networks in Figure 1F? Also, would it be more correct to say that the genes listed (i.e. GRIA1, GRIA2 etc) were identified as hub genes from these gene sets, rather than that hub analysis revealed the downregulation of these gene per-se?
A:
These comments have been addressed alongside the previous comments.

page 9, "Because of the enrichment of synaptic molecules in downregulated genes associated with AD, we further examined presynaptic and postsynaptic genes among the 129 late-onset AD patients and 101 non-demented healthy controls. …. The downregulation of selective synaptic genes in AD suggests that these transcriptional changes are not due to the general loss of synapses."

I am not an expert in synapse biology, however I understand that this paragraph concludes that the selective down-regulation of synaptic genes argues for transcriptional changes which are not simply due to the general loss of synapses. Would I understand correctly that there is also selective down-regulation of SNARE complex genes, with some SNARE complex genes being *not* changed in AD brains (e.g. STX2, SYN1, VAMP3) - if so, would it be worth commenting on possible reasons and meaning for only selective down-regulation of these SNARE complex genes?
A:
Based on our current analysis and previous studies (e.g. Mathys et al., 2019, Nature), we hypothesize that loss of synaptic function in AD is not just a product of the general loss of synapses. Instead, we suggest that there are specific attenuations of synaptic components/pathways that directly influence synaptic function. Here we show that the reduced expression of core SNARE components is one of the dysregulated pathways that causes synaptic dysfunction in AD. There are other synaptic genes that are either unchanged or even increased in AD.

page 9, "To classify gene networks that demonstrate functional enrichment in AD, we used the modular co-expression analysis, which allowed us to create weighted gene networks that unbiasedly generate interactions of genes whose expression is highly correlated to one another."

I might avoid the word "interactions" here - if I understand correctly, co-expression analysis derives modules of genes which share similar expression patterns; however, it does not determine that the products of those genes interact with each other.
A:
We have avoided the word "interactions" here, based on this reviewer's suggestion.

page 9, "Eigengene expression (Figure 3B) showed that the most enriched module for downregulated genes in AD was M1 (SNARE-binding), while the most enriched module for upregulated genes in AD was M2 (Death receptor activity)."

Here, Module 1 has been labelled as "SNARE-binding", I presume as a result of this being the top term for the module in the GO enrichment analysis mentioned a couple of paragraphs later (similarly for the labels for the other modules). I think it would be good to state that explicitly here. This is particularly necessary because, if I have understood correctly from Supplementary Table 3, only 9 out of the 522 genes in Module 1 are annotated with the GO term "SNARE binding"? I think it would be good to add a graph to Figure 3 indicating the number, or proportion, of genes in each module that are annotated with the term with which that module is labelled.

Also, I'm not sure that the word "enriched" should necessarily be used in this sentence. Figure 3B shows (I believe) that mean eigengene expression in Module 1 shows the greatest negative difference between AD and control samples, compared to all other modules (and similarly, Module 2 shows the greatest positive difference in mean eigengene expression between AD and control samples, compared to all other modules); however, I'm not sure that this says anything about enrichment of one particular set of genes compared to another. (I would understand "enrichment" to indicate that a statistical enrichment test had been performed).

A:

We have removed the term "enriched" to more clearly explain changes in eigengene expression for each module. Fig. 3B does show that Module 1 shows the greatest negative difference in mean eigengene expression between AD and control samples, while Module 2 shows the greatest positive difference between AD and control samples.

page 9, "Among the M1 DEGs with fold-change of at least 10%, 198 were downregulated and 100 were upregulated."

I am a bit confused here. M1 is a module of 522 genes which share a common expression pattern across samples. Therefore I would have expected genes in M1 to show a similar pattern of expression in AD samples as compared to control samples; however some genes in M1 are strongly up-regulated in AD vs control, and some are strongly down-regulated in AD vs control. I may well be misunderstanding but how can, for example, GFAP and SYN2 share a common-expression pattern across samples, when GFAP is higher in AD vs control and SYN2 is lower in AD vs control? It may be worth including, in the Supplemental Material, plots of gene expression across all samples (for M1 at least), highlighting the expression pattern of the eigengene, and a few example genes such as GFAP and SYN2, to show how their expression pattern is related.

A:

We appreciate the concern in this comment, and feel the reviewer's question highlights where some confusion may exist. Eigengene expression is a summary score, representing a single quantitative value for a group of elements. So here, each sample has a score for each module, representing how each sample is related to each module. When you take the mean of these scores for each group, we can see how well AD or Ctrl is associated with that module, or more directly compare the expression levels of each module for AD vs Ctrl. As shown in figure 3B, AD has a much lower eigengene expression in M1 when compared to control. A heatmap containing normalized eigengene expression values for patient samples in each module has been provided (see new **Sup. Fig 1**).

page 10, "In conjunction with this, these eight genes form a highly connected network (Figure 3F), supporting our previous identification of SNARE complex genes as the top-ranking downregulated clusters in PFC of human AD"

Could it be clarified what was the network analysis that produced Figure 3F? (Was it looking for interactions between these genes in STRING?). Again, I am not a synaptic biology expert, but is it surprising that these gene products all interact with each other, if they form part of the SNARE complex? Also I am not sure that the first part of this sentence ("these eight genes form a highly connected network") does necessarily logically support the second part ("identification of SNARE complex genes as the top-ranking downregulated clusters in PFC of human AD").
A:
The network analysis that produced Figure 3F utilized STRING to visualize the connection between genes contained in the SNARE-binding process. Because this figure panel represents the SNARE-binding functional group, this network is expected to be highly connected. However, the focus of the entire figure is to demonstrate that in using an alternative bioinformatic approach (modular analysis), then narrowing down to the top module (M1), we came up with similar findings as in Figure 1. We chose to create a network of the SNARE-binding genes to emphasize that core SNARE molecules are of particular importance in AD. We have reworded as "Among the 129 AD patients and 101 non-demented controls, all the eight SNARE-binding genes were significantly downregulated (**Figure 3E** and **3F**). These findings support our previous identification of SNARE complex genes as the top-ranking downregulated clusters in PFC of human AD, and further highlights the dysregulation of SNARE-mediated exocytotic function in AD".

page 10, "The most enriched convergent pathways among the three groups are exocytosis and synaptic signaling (Figure 4C), confirming the loss of presynaptic function in PFC excitatory and inhibitory neurons."

I wasn't sure what group of genes has been used to produced the enrichment results in Fig 4C - could this be clarified (is it the 39 common D.E. genes)?
A:
Here, all three DEG lists (Bulk (463), Ex. Neuron (342), In. Neuron (429)) were deposited to Metaescape. From here, the Metaescape software consolidated the gene list into one "FINAL" list, in which enrichment analysis was then performed as followed: "When multiple gene lists are provided, all lists are merged into one list called "_FINAL". A term may be found enriched in several individual gene lists and/or in the _FINAL gene list, and the best p-value among them is chosen as the final p-value. The pathway/process clusters that are found to be of interest (either shared or unique based on specific list enrichment) are used to prioritize the genes that fall into those clusters (membership is presented as 1/0 binary columns in the Excel spreadsheet). Note that individual gene lists containing more than 3000 genes are ignored during the enrichment analysis to avoid superficial terms; this is because long gene lists are often not random and generally trigger too many terms that are not of direct relevance to the biology under study." The methods were updated to more clearly explain how this pathway analysis on multiple gene list was performed.

page 10, "Hub analysis was performed to identify the primary driver genes common to bulk and neuronal DEGs."

While hub analysis ranks genes in cliques derived from interaction networks, I feel that it may be going too far based on this bioinformatic analysis to make statements that could be interpreted as causal (i.e. that these are "primary driver genes").
A:
We agree and have changed "primary driver genes" to “genes with the highest intra-molecular connectivity”.

page 11, "The common hub genes in bulk and neuronal PFC represent the loss of a network of synaptic molecules involved in exocytotic function and neurotransmission in human AD (Figure 4F)."

I think that I might say a "down-regulation" of this network, rather than "loss", unless expression of these genes is so reduced as to be nearly zero. (This comment applies generally).
A:
We have replaced “loss” with “down-regulation”, and rephrased the sentence.

page 12, "Synaptic vesicle genes, including SNAP-25, STX1A, SYNGR3, VAMP2, and STXBP1, are enriched in the list, with SNAP-25 having the greatest lost of expression."

Again, I would take the word "enrichment" to suggest that a statistical test for enrichment has been performed. If this is not the case, then I would suggest rewording.
A:
We have reworded the text and excluded “enrichment”.

page 12, "These genes represent a molecular network that can be targeted for therapeutic rescue of synaptic and cognitive function."

This is a strong statement, and I think that I would wish to see more mechanistic and causal studies beyond the bioinformatic analyses performed, in order to support. I would suggest that this statement might be made less emphatic.
A:
We have reworded the text as suggested.

page 12, "Quantative PCR was first conducted to examine the selected synaptic genes in PFC of postmortem tissues from AD human and control subjects."

Could it be explained further (a) what was the basis for choosing these particular genes in Fig 6A? (b) why only a subset of these were taken forward for Western blotting in Fig 6B, and PSD-95 included in Fig 6B but not in Fig 6A?

It would also be worth commenting on the fact that some genes previously found to be significantly down-regulated in AD (VAMP2, SYT1) were not down-regulated here, and a gene previously found to be not changed in AD (STX2) was down-regulated here.
A:
The basis of choosing three core SNARE components (STX1A, VAMP2, and SNAP-25) for Western blot and immunohistochemistry analysis is that the three genes were downregulated in our transcriptomic analysis and the three proteins are associated with cognitive stability. We have commented on the consistency and discrepancy of some genes in the revised manuscript.

page 13, "Since the 'early-pathology' AD group main exhibits the downregulation of genes exclusively in neurons, we have focused on the analyses of downregulated genes in AD that are potential targets for early intervention."

Comment: it is not clear to me that the genes studied here are necessarily potential targets for early intervention (although they may indeed be mainly neuronally-expressed), since they have been derived from studies of post-mortem tissue, where the changes seen in neuronally-expressed genes may be different from those seen in early disease?
A:
The scRNAseq data from PFC of individuals with varying degrees of AD pathology (Mathys et al., Nature, 2019) found that genes downregulated at early stages were neuron-specific, whereas genes upregulated at late stages were common across cell types and primarily involved in the global stress response. Since the alterations of neuronally-expressed synaptic genes appear early in pathological progression, we think they provide an early intervention target for AD treatment.

page 13, "Through co-expression modular analysis, we identified that the genes diminished in AD are most enriched in module 1 that contains SNARE-binding complex genes, including the core SNARE genes SNAP-25, STX1A and VAMP2."

However, VAMP2 was found *not* to be decreased in the quantitative PCR and Western blotting performed in this study. Could the authors comment on this?
A:
Our genomic analysis of a large number of human samples has discovered that all three core SNARE genes (*SNAP25*, *STX1A* and *VAMP2*) are downregulated. We did not find a significant reduction of *VAMP2* in qPCR and WB of our small scale human samples, which is likely due to the sample size differences and human sample variations. We have added comments on this.

page 13, "In addition to M1, downregulated genes in AD are also enriched in module 8..."

Again, it is not clear to me in what sense the word "enriched" is being used here - has a statistical test for enrichment been performed? If what is meant is that the mean eigengene expression in control samples in M8 is higher than in AD samples, I would argue against using the word enrichment.
A:
The term “enriched” has been removed. We have rephrased to indicate that M8 is also a module with low expression in AD.

Further Figure comments:

In general, I feel that the figures could benefit from more detailed legends describing in more depth what is being plotted (however, I don’t know if there are word count limits in play here).
A:
We have added more explanations in some legends.

Figures 1A, 1B, 1C. It would be nice to include tables of these enrichment results in the Supplement Materials, in a similar manner to Sup. Table 3.
A:
Enrichment results have been added and updated (see **new Supplementary Tables 1 & 2**).

Figure 1D. I'm a bit confused by what this graph is showing - is the total length of the bars equals to the number of genes in the category *plus* the -log10(q-value) ? If so, I don't think these are appropriate quantities for summing together. The number of genes and significance of enrichment would be better shown separately.

A:

We have modified Figure 1D as a bar graph representing only gene count (see **revised Fig. 1D**). The enrichment (-$\log_{10}$(q value)) has been shown in Figure 1C.

Figures 1E, 1F. What does the thickness of the lines linking genes signify?

A:

The thickness of the lines in the previous networks signified a weighted correlation between two genes. We have since updated this figure based on previous suggestions, and the updated figure no longer has these weighted connections (see **revised Fig. 1E**).

Figure 1F. There is a typo - "Glutamateric" should read "Glutamatergic"?

A:

We have corrected the typo.

Figures 2A, 2C. Are these heatmaps ordered (vertically) by significance (or magnitude perhaps?) of differential gene expression? It would be good to state this in the figure legend if so.

A:

Figures 2A and 2C are row clustered by k-means. This has been more clearly explained in Fig. 2 legend.

Figures 2B, 2D. For the benefit of those less familiar with microarray analysis, it would be good to state exactly what is being plotted on the y-axis - are these log2 intensities?

A:

The values shown here are $\log_2$ (gene expression, normalized to the average of controls). This information has been updated in the Figure 2 legend.

Figure 3A. As previously stated, it would be good to indicate what proportion or number of the genes in each module are annotated with the indicated GO terms. It would also to be good to include in the Supplemental Material tables analogous to Sup. Table 3, giving the GO analysis for the other modules (even perhaps just the top 5 or 10 terms for each).

A:

In Table 1, we have provided information for each module, including functional description of each module, the top genes in each module and the number of genes in each module.

Figure 3B. As for Figs 2B, 2D, it would be good to indicate exactly what quantity is being plotted for eigengene expression on the y-axis here.

A:

Fig. 3B y-axis is $\log_{10}$(normalized eigengene expression). We have clarified it in the figure legend.

Figure 3E. What was the criterion for the genes chosen here - are they the most significantly down-regulated? If so, could this be explicitly stated? Also, to avoid confusion, it might be helpful to use different colours for indicating membership in the clustergram on the one hand, and to highlight the names of the SNARE binding genes on the other.

A:

Fig. 3E, the criterion for the genes selected here are as followed: Module 1 genes were deposited in EnrichR where GO molecular function enrichment was performed. From there genes from the input list were sorted by significance, and the top 20 genes were selected and compared to the top 10 most enriched terms, with the blue boxes indicating gene occupancy in the GO category. We have used blue color for highlighting SNARE-related terms (see **revised Fig. 3E**). A more thorough explanation has been described in the methods section.

Figure 3F. The appearance of this network (and that in Figure 4F) is different to those in Figures 1E, F - does this indicate a different type of network analysis?
A:
Figure 3F is not highlighting hub genes but instead showing the interconnection of the SNARE-binding pathway. Figure 4F has been updated based on reviewer 1's comments (a network with only hub genes provides limited information) to reflect genes overlapped between the three groups, in addition to highlighting SNAP25 as a top-ranking hub gene (see **revised Fig. 4F**).

Figure 4D. The colours in this heatmap (dark blue or yellow) appear to indicate that all the indicated genes are either up-regulated almost exactly 2-fold, or down-regulated exactly 2-fold. Is that true?
A:
Thanks for pointing out a mishap on our part with our scale when the heatmap was produced. We have adjusted the scale to appropriately account for the correct range, and have replaced this heatmap with the more precise one (see **revised Fig. 4D**).

Figure 4E. I'm not sure that it makes sense to me to plot the "combined absolute fold change" for these genes. I think that it would be better to use separate bars for the fold changes of the three data sets, and to plot the log2 fold change for each.
A:
We have separated the bar graphs and plotted $\log_2$ fold change values (see **revised Fig. 4E**).

Figure 5F. This is titled "Common Gene Sets" - should that just be "Common genes" (i.e. the 20 common genes shown in Figure 5E).
A:
Fig. 5F has been changed to "Common genes".

Figure 6A. The legend states "n = 6 pairs" - are these samples actually paired, or should that read "ctrl: n=6, AD: n=6"?
A:
We have changed to "ctrl: n=6, AD: n=6".

Figure 7 (part 4). There is a typo - "Cognitive" should read "Cognitive".
A:
The typo in Fig. 7 (part 4) has been corrected.

Editorial Office comments:

-Please supply a graphical abstract. A graphical abstract is a visual representation of the central finding or methodology of the paper. It will be displayed in the table of contents. Authors should strive to make the graphical abstract informative, interesting, visually appealing, and straightforward (no caption

needed, no abbreviations except gene names). Please upload as a separate high resolution image file (tiff preferred). The technical requirements for the graphical abstracts are:
--size of the submitted image: 1200 pixels square at 300dpi
--font of the text: Arial 12-16 point (smaller fonts will not be legible online)

A:

A graphical abstract (image and text) has been supplied.

-Please add an Abbreviated Summary (up to 50 words) of your paper that captures the main purpose and conclusions of the work. The summary should state the main results written in the third person (‘[1st author surname] et al. report that …’) and conclusion. This summary will be used in the contents list online and should not contain any abbreviations other than those on the accepted abbreviations list and accepted gene/protein names, making it accessible to the non-specialist. Please upload it as a Word document. Allowed abbreviations: AIDS; ANOVA; AMPA; ATP; A,T,C,G; CNS; CSF; CT; DNA; ECG; EEG; EMG; GABA; HIV; MRI; NMDA; PET; PCR; RNA.

A:

An Abbreviated Summary has been added.

- To comply with our ethical standards, the appropriate checklist must be included (see Reporting Guideline section within 'Instructions to authors').

A:

The checklist has been included.

-Please provide the genders of the patients in the study.

A:

The genders of the patients in the study have been provided.

- Fig6b: please, provide Full- size and uncropped blots/gels as Supplementary material.

A:

Fig. 6b: The Full- size and uncropped blots/gels of all detected samples have been provided (see **new Supplementary Figure 2**).
