# Supplementary material for: Transcriptomic analysis of human brains with Alzheimer’s disease reveals the altered expression of synaptic genes linked to cognitive deficits
Source: Brain Commun. 2021 Jun 3;3(3):fcab123. doi: 10.1093/braincomms/fcab123 (PMC8374979; doi:10.1093/braincomms/fcab123)
Supplement: fcab123_Supplementary_Data [file Original Submission-Decision Letter.pdf › Original Submission-Decision Letter.pdf]

Original Submission – Decision Letter

14-Jan-2021

Dear Dr. Yan,

Thank you for your recent submission BRAINCOM-2020-418 entitled "Transcriptomic Analysis of Human Brains with Alzheimer’s Disease Reveals the Key Determinant for Cognitive Deficits" to Brain Communications.

Your manuscript has been reviewed, and while the reviewers and editorial team appreciate many aspects of the paper, they raise some concerns that you will need to address in order for the paper to be considered for publication.

If you can address these concerns, I invite you to submit a revised manuscript and a response to each of the reviewers' comments. The reviewers’ comments are included at the bottom of this message. In particular for Brain Communications, please be sure to thoroughly address the statistical issues raised (correction for multiple testing) and to carefully interpret your data to be sure conclusions are fully supported.

In addition to the reviewers' comments, please note our editorial comments added below to comply with our requirements for presentation of scientific data at Brain Communications.

Please format your revised manuscript according to our Instructions to Authors https://academic.oup.com/braincomms/pages/General_Instructions – a summary of key points is attached to help with this.

To revise your manuscript, click the link below or log into https://mc.manuscriptcentral.com/braincom and enter your Author Centre, where you will find your manuscript title listed under "Manuscripts with Decisions." Under "Actions," click on "Create a Revision." Your manuscript number has been appended to denote a revision.

*** PLEASE NOTE: This is a two-step process. After clicking on the link, you will be directed to a webpage to confirm. ***

https://mc.manuscriptcentral.com/braincom?URL_MASK=60642fcd2839487197a1582bb8fdfb38

Please upload a revised manuscript with changes tracked with track changes or in coloured text.

When submitting your revised manuscript, you will be asked to respond to the reviewer(s) comments. Please be as specific as possible in your response to all of the comments.

The editorial team at Brain Communications aims to increase transparency in scientific publishing and to demonstrate the value that peer review brings to the scientific process. Please therefore consider whether you are willing to have reviewer(s)’ comments on your paper and your response published as supplementary data with the paper if it is accepted. This is entirely optional and will only be done if both the authors and reviewers agree. While submitting your revision there will be an opportunity to review or update the answer you gave previously, if necessary.

IMPORTANT: Your original files are available to you when you upload your revised manuscript. Please delete any redundant files before completing the submission.

Because we are trying to facilitate timely publication of manuscripts submitted to Brain Communications, we would appreciate your revised manuscript as soon as possible. If it is not possible for you to resubmit within 3 months, the option to submit a revision will expire, and we would then consider your revised paper as a new submission. However, if you need a little more time, please get in touch and we can extend this deadline for you.

Once again, thank you for submitting your manuscript to Brain Communications and I look forward to receiving your revision.

Yours sincerely,

Prof. Tara Spires-Jones
Editor, Brain Communications

Reviewer(s)' Comments to Author:

Reviewer: 1 (Becky Carlyle)

Comments to the Author
In this manuscript, Williams et al. use a series of bioinformatic tools in an analysis of older microarray data, to highlight the role that synaptic transcripts and protein play in Alzheimer's Disease pathogenesis. The outcome of this analysis is then compared with a single cell results set and a proteomic results set. This work uses a substantial number of tools and approaches, some of which I am not familiar with, but all the approaches return to the central theme that synapses are critical to Alzheimer's disease pathogenesis.

Major issues
1) My biggest issue with the manuscript is that is it unclear where the previous analysis of this dataset ends and this new analysis begins. In looking at the Cell 2013 paper, it seems that they used linear modeling that took into account age, PMI, sex etc in performing differential expression analysis. The analysis in this paper uses only a subset of the samples from the original paper, and I am uncertain whether the p values used in this re-analysis came from the 2013 paper, or whether a new differential expression analysis was performed. This needs clarifying more strongly in both the methods and the results.
2) Regardless of how the p-values were computed, it does not appear that they were corrected for multiple testing. If they were, and this is an error on my part, it should be very clearly stated in the paper in both the methods and the results. The original microarray included probes for just under 40,000 genes, and around 2000 of them were found to have a p value of 0.05 or below. This is pretty much exactly the number of genes you would expect to find by chance. pValue correction should absolutely be performed before continuing to take these genes forwards to ontology analysis.
3) For the network diagrams showing hub proteins (Figure 1 E & F) can these hubs be shown on a network background of the non-hub DEG genes in those ontological categories? These particular plots don't show much, other than the hub proteins are connected to each other. It would be more informative to see that they were, in fact, each connected to a number if other DEGs, increasing confidence in the hub analysis.
4) I'm not used to seeing heatmaps like those in Figure 2A and C. I think I find the lack of clustering jarring – why was the decision made to not use clustering, at least for the rows? I am unclear as to exactly how the data are scaled – are they scaled and centered, or is some kind of ranking being used? Or is there no scaling? Please make this clearer. It would also be interesting to see how well AD clusters from Normal, rather than separating them as has been done here.
5) The in-house confirmation experiments in Figure 6 are poorly described and need more details.
a. What are the demographics of the samples? Were they well matched for age, which is another determinant of synapse density?
b. What method of microscopy was used for the imaging? What antibodies?
c. What do the error bars on 6A show? SEM?
d. All lanes of the western blots should be shown, if there are only 11 samples total
e. The imaging focuses on the cell bodies of the neurons, and NeuN is primarily a nuclear marker for neurons. See Figure 1E and 1F here for someone who has attempted something similar using HRP methods: https://pubmed.ncbi.nlm.nih.gov/22554416/#&gid=article-figures&pid=figure-1-uid-0

Minor issues
1) The methods mention GADPH as a western blot antibody – there is no band shown for GADPH on the figure, but there is a band for ACTIN. Which was used? For future reference, it is no longer considered best practice to normalize western blots to a single reference protein (and I have seen that GAPDH commonly changes in AD vs control), a total protein stain should be used instead. See the blot section here: http://jbcresources.asbmb.org/collecting-and-presenting-data

2) Spelling correction needed on 1F title

3) Was there information on cognitive status of the cases used for the original study, or were they all neuropathology confirmed only? Given up to one thirds of community dwelling individuals may have AD-level pathology but no evidence of cognitive impairment, this is important.

4) For figure 3B, is it possible to plot a single enrichment in AD value for each module rather that the bi-directional plots? It's very difficult to visually compare the two blots and assess the enrichment.

5) The process of Figure 3 currently seems a bit circular. I'm not familiar with the webCEMiTool, but it looks like it probably identified covarying genes/proteins, places them into modules, then look for over-representation of GO terms in these modules – this is your Figure 3A. So then when you take proteins from M1 and do your own GO analysis to find the same GO category at the top, this seems a little circular. Perhaps you could make it a little clearer what Figure 3D adds to the analysis?

6) It would be great to have a bigger version of plot 4B (Circos plot) in the supplement with the gene names on the exterior of the circle – I don't find this unlabelled version to be particularly informative.

7) Figure 4E as a stacked plot is very difficult to interpret – would be better unstacked. Stacking works well in 5A where you're talking about a proportion of a whole, less well in comparing fold changes across different cell types.

8) Not a note for this paper but more general advice: I would advise the author to start moving away from using web tools and to hard code their analyses in their preferred coding language. Code can then be provided through github, bitbucket or similar. Many of these tools have associated R packages and integration, for example. This would help with reproducibility and access to the data for higher-impact papers in their future, and also helps them build analytical pathways in house that can be accessed repeatedly much more easily than using web tools.

Reviewer: 2

Comments to the Author
In this manuscript, the authors conduct an integrative analysis of microarray, single nucleus RNA-seq, and proteomics data sets, to study the role of aberrant gene expression in AD and its relation to cognitive trajectory. My comments will mainly pertain to the bioinformatics analyses performed, terminology used, and the figures; I feel that some improvements can be made to clarify these.

I would also make the overall comment that I would be careful to ensure that the strength of statements and central claims made (for example, that the key determinant for cognitive deficits in Alzheimer's has been revealed) are fully supported and justified by the analyses performed.

Specific comments:

page 8, "Analyses of the transcriptomic data with a cutoff of $p < 0.05$ and fold change (FC) of 10% identified 2174 differentially expressed genes (DEGs) (1241 down; 933 up). "

Is the p-value cut-off of < 0.05 mentioned here a raw p-value, or a multiple-testing corrected "adjusted" p-value? If it is an adjusted p-value, this should be explicitly mentioned, and if not, it should be mentioned why a multiple-testing corrected p-value threshold was not used (since this would be standard practice). It would also be a valuable resource to include the full differential expression results (p-values, adjusted p-values, fold changes) as a Supplemental Table.

page 8, "GO enrichment analyses of these significant DEGs indicated that the most prominently down-regulated genes in AD were those involved in synaptic signaling, synapse organization, ion transport, neurotransmitter secretion, and glutamatergic synaptic transmission (Figure 1A). On the other hand, the most prominently up- regulated genes in AD were those involved in immune response activation and cell death (Figure 1B)"

"Prominently" here might suggest that the genes annotated with these GO terms are the genes with the smallest p-values or largest fold changes, but that is not necessarily the case. I think it might be better to simply say that these GO terms were the most significantly enriched (unless a statement about p-values or fold changes *is* being made).

page 8, "With synaptic signaling being the most significantly downregulated GO category, we further explored dysregulated synaptic pathways in AD. Among the downregulated DEGs in AD, 239 were classified as synaptic genes (Sup. Table 1). Synaptic enrichment analyses identified presynapse as the most enriched and abundant subcellular component downregulated in AD (Figure 1C and 1D). Within the presynaptic gene cluster, synaptic vesicle-related genes were the most overrepresented subcategory (Figure 1C, -Log10 p-value = 14.37)."

It would be good to state what background gene set was used for the synaptic enrichment analysis (e.g. brain expressed genes vs only synaptic genes) as this makes a difference to interpretation of the enrichment p-values obtained.

page 8, "Hub co-expression analyses were performed on the 41 downregulated synaptic vesicle-clustered genes (Figure 1E), and identified ten genes (SNAP-25, SYT1, VAMP2, STX1A, SYN2, SYT11, SLC32A1, RAB3C, SV2B, and SH3GL2) that were lost in PFC of AD patients, with SNAP-25 as the top-ranking node."

It would be great to state more explicitly, particularly for the benefit of non-bioinformatician readers,

what hub co-expression analysis constitutes. I think from the Methods section, that this involves using the STRING database to construct an interaction network of the 41 down-regulated synaptic vesicle-clustered genes, and then using MCC to identify hub genes in this network? But I'm not clear what role co-expression plays in this?

page 9, "Hub co- expression analyses also revealed the downregulation of synaptic genes encoding receptors, transporters and enzymes mediating glutamatergic transmission (e.g. GRIA1, GRIA2, GRIN2A, and SLC17A6) or GABAergic transmission (e.g. GAD2, GABRB2, and SLC6A1) in AD (Figure 1F)."

I'm unsure as to which sets of genes formed input for the hub analyses that produced the networks in Figure 1F? Also, would it be more correct to say that the genes listed (i.e. GRIA1, GRIA2 etc) were identified as hub genes from these gene sets, rather than that hub analysis revealed the downregulation of these gene per-se?

page 9, "Because of the enrichment of synaptic molecules in downregulated genes associated with AD, we further examined presynaptic and postsynaptic genes among the 129 late-onset AD patients and 101 non-demented healthy controls. As shown in Figure 2A and 2B, many of the SNARE complex genes involved in presynaptic vesicle exocytosis were significantly downregulated in AD, including SNAP-25, STX1A, SYT1 and VAMP2, while STX2, SYN1, and VAMP3 were not changed. Some genes encoding postsynaptic glutamate receptors, GABAA receptors or anchoring proteins were also significantly downregulated in AD, including GRIN2A, GRIA1, GRIA2, GABRA1, GABRB2, GRM3, and SHANK2, while GRM5, SYNGAP1, SHANK3, and GRIN2B were not changed (Figure 2C and 2D). The downregulation of selective synaptic genes in AD suggests that these transcriptional changes are not due to the general loss of synapses."

I am not an expert in synapse biology, however I understand that this paragraph concludes that the selective down-regulation of synaptic genes argues for transcriptional changes which are not simply due to the general loss of synapses. Would I understand correctly that there is also selective down-regulation of SNARE complex genes, with some SNARE complex genes being *not* changed in AD brains (e.g. STX2, SYN1, VAMP3) - if so, would it be worth commenting on possible reasons and meaning for only selective down-regulation of these SNARE complex genes?

page 9, "To classify gene networks that demonstrate functional enrichment in AD, we used the modular co-expression analysis, which allowed us to create weighted gene networks that unbiasedly generate interactions of genes whose expression is highly correlated to one another."

I might avoid the word "interactions" here - if I understand correctly, co-expression analysis derives modules of genes which share similar expression patterns; however, it does not determine that the products of those genes interact with each other.

page 9, "Eigengene expression (Figure 3B) showed that the most enriched module for downregulated genes in AD was M1 (SNARE-binding), while the most enriched module for upregulated genes in AD was M2 (Death receptor activity)."

Here, Module 1 has been labelled as "SNARE-binding", I presume as a result of this being the top term for the module in the GO enrichment analysis mentioned a couple of paragraphs later (similarly for the labels for the other modules). I think it would be good to state that explicitly here. This is particularly necessary because, if I have understood correctly from Supplementary Table 3, only 9 out of the 522 genes in Module 1 are annotated with the GO term "SNARE binding"? I think it would be good to add a graph to Figure 3 indicating the number, or proportion, of genes in each module that are annotated with the term with which that module is labelled.

Also, I'm not sure that the word "enriched" should necessarily be used in this sentence. Figure 3B shows (I believe) that mean eigengene expression in Module 1 shows the greatest negative difference between AD and control samples, compared to all other modules (and similarly, Module 2 shows the greatest positive difference in mean eigengene expression between AD and control samples, compared to all other modules); however, I'm not sure that this says anything about enrichment of one particular set of genes compared to another. (I would understand "enrichment" to indicate that a statistical enrichment test had been performed).

page 9, "Among the M1 DEGs with fold-change of at least 10%, 198 were downregulated and 100 were upregulated."

I am a bit confused here. M1 is a module of 522 genes which share a common expression pattern across samples. Therefore I would have expected genes in M1 to show a similar pattern of expression in AD samples as compared to control samples; however some genes in M1 are strongly up-regulated in AD vs

control, and some are strongly down-regulated in AD vs control. I may well be misunderstanding but how can, for example, GFAP and SYN2 share a common-expression pattern across samples, when GFAP is higher in AD vs control and SYN2 is lower in AD vs control? It may be worth including, in the Supplemental Material, plots of gene expression across all samples (for M1 at least), highlighting the expression pattern of the eigengene, and a few example genes such as GFAP and SYN2, to show how their expression pattern is related.

page 10, "In conjunction with this, these eight genes form a highly connected network (Figure 3F), supporting our previous identification of SNARE complex genes as the top-ranking downregulated clusters in PFC of human AD"

Could it be clarified what was the network analysis that produced Figure 3F? (Was it looking for interactions between these genes in STRING?). Again, I am not a synaptic biology expert, but is it surprising that these gene products all interact with each other, if they form part of the SNARE complex? Also I am not sure that the first part of this sentence ("these eight genes form a highly connected network") does necessarily logically support the second part ("identification of SNARE complex genes as the top-ranking downregulated clusters in PFC of human AD").

page 10, "The most enriched convergent pathways among the three groups are exocytosis and synaptic signaling (Figure 4C), confirming the loss of presynaptic function in PFC excitatory and inhibitory neurons."

I wasn’t sure what group of genes has been used to produced the enrichment results in Fig 4C - could this be clarified (is it the 39 common D.E. genes)?

page 10, "Hub analysis was performed to identify the primary driver genes common to bulk and neuronal DEGs."

While hub analysis ranks genes in cliques derived from interaction networks, I feel that it may be going too far based on this bioinformatic analysis to make statements that could be interpreted as causal (i.e. that these are "primary driver genes").

page 11, "The common hub genes in bulk and neuronal PFC represent the loss of a network of synaptic molecules involved in exocytotic function and neurotransmission in human AD (Figure 4F)."

I think that I might say a "down-regulation" of this network, rather than "loss", unless expression of these genes is so reduced as to be nearly zero. (This comment applies generally).

page 12, "Synaptic vesicle genes, including SNAP-25, STX1A, SYNGR3, VAMP2, and STXBP1, are enriched in the list, with SNAP-25 having the greatest lost of expression."

Again, I would take the word "enrichment" to suggest that a statistical test for enrichment has been performed. If this is not the case, then I would suggest rewording.

page 12, "These genes represent a molecular network that can be targeted for therapeutic rescue of synaptic and cognitive function."

This is a strong statement, and I think that I would wish to see more mechanistic and causal studies beyond the bioinformatic analyses performed, in order to support. I would suggest that this statement might be made less emphatic.

page 12, "Quantative PCR was first conducted to examine the selected synaptic genes in PFC of postmortem tissues from AD human and control subjects."

Could it be explained further (a) what was the basis for choosing these particular genes in Fig 6A? (b) why only a subset of these were taken forward for Western blotting in Fig 6B, and PSD-95 included in Fig 6B but not in Fig 6A?

It would also be worth commenting on the fact that some genes previously found to be significantly down-regulated in AD (VAMP2, SYT1) were not down-regulated here, and a gene previously found to be not changed in AD (STX2) was down-regulated here.

page 13, "Since the 'early-pathology' AD group main exhibits the downregulation of genes exclusively in neurons, we have focused on the analyses of downregulated genes in AD that are potential targets for early intervention."

Comment: it is not clear to me that the genes studied here are necessarily potential targets for early

intervention (although they may indeed be mainly neuronally-expressed), since they have been derived from studies of post-mortem tissue, where the changes seen in neuronally-expressed genes may be different from those seen in early disease?

page 13, "Through co-expression modular analysis, we identified that the genes diminished in AD are most enriched in module 1 that contains SNARE-binding complex genes, including the core SNARE genes SNAP-25, STX1A and VAMP2."

However, VAMP2 was found *not* to be decreased in the quantitative PCR and Western blotting performed in this study. Could the authors comment on this?

page 13, "In addition to M1, downregulated genes in AD are also enriched in module 8…"

Again, it is not clear to me in what sense the word "enriched" is being used here - has a statistical test for enrichment been performed? If what is meant is that the mean eigengene expression in control samples in M8 is higher than in AD samples, I would argue against using the word enrichment.

Further Figure comments:

In general, I feel that the figures could benefit from more detailed legends describing in more depth what is being plotted (however, I don't know if there are word count limits in play here).

Figures 1A, 1B, 1C

It would be nice to include tables of these enrichment results in the Supplement Materials, in a similar manner to Sup. Table 3.

Figure 1D

I'm a bit confused by what this graph is showing - is the total length of the bars equals to the number of genes in the category *plus* the -log10(q-value) ? If so, I don't think these are appropriate quantities for summing together. The number of genes and significance of enrichment would be better shown separately.

Figures 1E, 1F

What does the thickness of the lines linking genes signify?

Figure 1F

There is a typo - "Glutamateric" should read "Glutamatergic"?

Figures 2A, 2C

Are these heatmaps ordered (vertically) by significance (or magnitude perhaps?) of differential gene expression? It would be good to state this in the figure legend if so.

Figures 2B, 2D

For the benefit of those less familiar with microarray analysis, it would be good to state exactly what is being plotted on the y-axis - are these log2 intensities?

Figure 3A

As previously stated, it would be good to indicate what proportion or number of the genes in each module are annotated with the indicated GO terms. It would also to be good to include in the Supplemental Material tables analogous to Sup. Table 3, giving the GO analysis for the other modules (even perhaps just the top 5 or 10 terms for each).

Figure 3B

As for Figs 2B, 2D, it would be good to indicate exactly what quantity is being plotted for eigengene expression on the y-axis here.

Figure 3E

What was the criterion for the genes chosen here - are they the most significantly down-regulated? If

so, could this be explicitly stated? Also, to avoid confusion, it might be helpful to use different colours for indicating membership in the clustergram on the one hand, and to highlight the names of the SNARE binding genes on the other.

Figure 3F

The appearance of this network (and that in Figure 4F) is different to those in Figures 1E, F - does this indicate a different type of network analysis?

Figure 4D

The colours in this heatmap (dark blue or yellow) appear to indicate that all the indicated genes are either up-regulated almost exactly 2-fold, or down-regulated exactly 2-fold. Is that true?

Figure 4E

I'm not sure that it makes sense to me to plot the "combined absolute fold change" for these genes. I think that it would be better to use separate bars for the fold changes of the three data sets, and to plot the log2 fold change for each.

Figure 5F

This is titled "Common Gene Sets" - should that just be "Common genes" (i.e. the 20 common genes shown in Figure 5E).

Figure 6A

The legend states "n = 6 pairs" - are these samples actually paired, or should that read "ctrl: n=6, AD: n=6"?

Figure 7 (part 4)

There is a typo - "Cogintive" should read "Cognitive".

Editorial Office comments:

-Please supply a graphical abstract. A graphical abstract is a visual representation of the central finding or methodology of the paper. It will be displayed in the table of contents. Authors should strive to make the graphical abstract informative, interesting, visually appealing, and straightforward (no caption needed, no abbreviations except gene names). Please upload as a separate high resolution image file (tiff preferred). The technical requirements for the graphical abstracts are:
--size of the submitted image: 1200 pixels square at 300dpi
--font of the text: Arial 12-16 point (smaller fonts will not be legible online)

-Please add an Abbreviated Summary (up to 50 words) of your paper that captures the main purpose and conclusions of the work. The summary should state the main results written in the third person ('[1st author surname] et al. report that …') and conclusion. This summary will be used in the contents list online and should not contain any abbreviations other than those on the accepted abbreviations list and accepted gene/protein names, making it accessible to the non-specialist. Please upload it as a Word document. Allowed abbreviations: AIDS; ANOVA; AMPA; ATP; A,T,C,G; CNS; CSF; CT; DNA; ECG; EEG; EMG; GABA; HIV; MRI; NMDA; PET; PCR; RNA.

- To comply with our ethical standards, the appropriate checklist must be included (see Reporting Guideline section within 'Instructions to authors').

-Please provide the genders of the patients in the study.

- Fig6b: please, provide Full- size and uncropped blots/gels as Supplementary material.
