# Supplementary material for: Transcriptomic analysis of human brains with Alzheimer’s disease reveals the altered expression of synaptic genes linked to cognitive deficits
Source: Brain Commun. 2021 Jun 3;3(3):fcab123. doi: 10.1093/braincomms/fcab123 (PMC8374979; doi:10.1093/braincomms/fcab123)
Supplement: fcab123_Supplementary_Data [file Revision 1 – Decision Letter.pdf › Revision 1 – Decision Letter.pdf]

Revision 1 – Decision Letter

22-Feb-2021

Dear Dr. Yan,

Thank you for your recent revised submission BRAINCOM-2020-418.R1 entitled "Transcriptomic Analysis of Human Brains with Alzheimer's Disease Reveals the Key Determinant for Cognitive Deficits" to Brain Communications.

The original reviewers have reviewed your revision, and while they agree that the paper is improved, there are several remaining issues that you will need to address. Therefore, I invite you to respond to the reviewers' comments and revise your manuscript. The reviewers' comments are included at the bottom of this message.

In addition to the reviewers' comments, please note our editorial comments added below to comply with our requirements for presentation of scientific data at Brain Communications.

Please format your revised manuscript according to our Instructions to Authors https://academic.oup.com/braincomms/pages/General_Instructions – a summary of key points is attached to help with this.

To revise your manuscript, click the link below or log into https://mc.manuscriptcentral.com/braincom and enter your Author Centre, where you will find your manuscript title listed under "Manuscripts with Decisions." Under "Actions," click on "Create a Revision." Your manuscript number has been appended to denote a revision.

*** PLEASE NOTE: This is a two-step process. After clicking on the link, you will be directed to a webpage to confirm. ***

https://mc.manuscriptcentral.com/braincom?URL_MASK=7837fa5888ab4fe6bb90f80febda16a9

Please upload a revised manuscript with changes tracked using track changes or in coloured text.

When submitting your revised manuscript, you will be asked to respond to the reviewer(s) comments. Please be as specific as possible in your response to all of the comments.

The editorial team at Brain Communications also aims to increase transparency in scientific publishing and to demonstrate the value that peer review brings to the scientific process. Please therefore consider whether you are willing to have reviewer comments on your paper and your response published as supplementary data with the paper if it is accepted. This is entirely optional and will only be done if both the authors and reviewers agree. When submitting your revision you will have the opportunity to update the answer you entered previously.

IMPORTANT: Your original files are available to you when you upload your revised manuscript. Please delete any redundant files before completing the submission.

Because we are trying to facilitate timely publication of manuscripts submitted to Brain Communications, we would appreciate your revised manuscript as soon as possible. If it is not possible for you to resubmit within 3 months, the option to submit a revision will expire, and we would then consider your revised paper as a new submission. However, if you need a little more time, please get in touch and we can extend this deadline for you.

Once again, thank you for submitting your manuscript to Brain Communications and I look forward to receiving your revision.

Yours sincerely,

Prof. Tara Spires-Jones
Editor, Brain Communications

Reviewer(s)' Comments to Author:

Reviewer: 1 (Becky Carlyle)

Comments to the Author

I thank the authors for their diligent response to my review comments, a good number of which were adopted. I still have some remaining issues, the most substantial concerning the biological follow ups in Figure 6. These are attached in a separate file.

Reviewer: 2

Comments to the Author
My thanks to the authors for the changes that they have made to the manuscript in response to both reviewers' comments. I still have a few concerns (mostly related to the gene co-expression network analysis), which I think should be relatively easy to address.

<i>page 8, "With synaptic signaling being the most significantly downregulated GO category, we further explored dysregulated synaptic pathways in AD. .... (Figure 1C and 1D). ... (Figure 1C, -Log10 p-value = 14.37)."

It would be good to state what background gene set was used for the synaptic enrichment analysis (e.g. brain expressed genes vs only synaptic genes) as this makes a difference to interpretation of the enrichment p-values obtained.

A:
We have added the statement about what background gene set was used for the synaptic enrichment analysis.</i>

My apologies if I have missed it, or have been unclear in my previous comment, but I would still appreciate a statement of the background gene set used in the SynGo enrichment analysis. My assumption would be that the whole set of brain expressed genes has been used as the background, as this is the default on the SynGo website (the alternative would be a custom gene list supplied by the user).

<i>page 8, "Hub co-expression analyses were performed on the 41 downregulated synaptic vesicle-clustered genes (Figure 1E), ... with SNAP-25 as the top-ranking node."

It would be great to state more explicitly, particularly for the benefit of non-bioinformatician readers, what hub co-expression analysis constitutes. I think from the Methods section, that this involves using the STRING database to construct an interaction network of the 41 down-regulated synaptic vesicle-clustered genes, and then using MCC to identify hub genes in this network? But I'm not clear what role co-expression plays in this?

A:
Figures 1E and 1F have been consolidated to highlight broader interactions of downregulated synaptic genes in AD (see revised Fig. 1E), as suggested by reviewer 1. In addition, we have revised the manuscript to more clearly explain what hub genes are and the relevance in our studies.</i>

Many thanks for the extra explanation of hub gene analysis. I am still unsure about the use of the word "co-expression" in the sentence following the new text, i.e. "Hub co-expression analyses also revealed…". Co-expression would imply to me that genes are linked in the network because they share similar expression patterns across samples; however, I believe that genes are linked in the network because their protein products are known or predicted to interact in the STRING database. It is certainly possible that I have misunderstood, but if not, should the word "co-expression" be removed here?

I think that it would also be great to expand the legend for Figure 1E to explain that the top 50 hub genes are being shown (as was detailed by the authors in a response to Reviewer 1).

<i>page 9, "Among the M1 DEGs with fold-change of at least 10%, 198 were downregulated and 100 were upregulated."

I am a bit confused here. M1 is a module of 522 genes which share a common expression pattern across samples. Therefore I would have expected genes in M1 to show a similar pattern of expression in AD samples as compared to control samples; however some genes in M1 are strongly up-regulated in AD vs control, and some are strongly down-regulated in AD vs control. I may well be misunderstanding but how can, for example, GFAP and SYN2 share a common-expression pattern across samples, when GFAP is higher in AD vs control and SYN2 is lower in AD vs control? It may be worth including, in the Supplemental Material, plots of gene expression across all samples (for M1 at least), highlighting the expression pattern of the eigengene, and a few example genes such as GFAP and SYN2, to show how their expression pattern is related.

A:

We appreciate the concern in this comment, and feel the reviewer's question highlights where some confusion may exist. Eigengene expression is a summary score, representing a single quantitative value for a group of elements. So here, each sample has a score for each module, representing how each sample is related to each module. When you take the mean of these scores for each group, we can see how well AD or Ctrl is associated with that module, or more directly compare the expression levels of each module for AD vs Ctrl. As shown in figure 3B, AD has a much lower eigengene expression in M1 when compared to control. A heatmap containing normalized eigengene expression values for patient samples in each module has been provided (see new Sup. Fig 1).</i>

I apologise for any lack of clarity in my previous comments: I still have a concern remaining that, for example, GFAP and SYN2 (which are regulated in <i>opposite</i> directions in AD vs control) are contained within the same co-expression module.

Further to their answer could the authors possibly confirm whether they have constructed a <i>signed</i> or an <i>unsigned</i> co-expression network (unfortunately I am not familiar with webCEMiTool, and hence do not know which type of network it is constructing). If their gene co-expression network is indeed unsigned, then that would alleviate my concern about GFAP and SYN2 being contained within the same module (because anti-correlation of expression across samples would then count as linkage in the network, and hence it would not necessarily be surprising to find these two genes in the same module).

However, if the network is a signed co-expression network, I would remain worried that GFAP and SYN2 (one of which is up-regulated, the other down-regulated) could be contained within the same module for the following reason. In my previous experience of using WGCNA (a similar tool) for constructing co-expression networks, I have sometimes found there to be a "default" module which contains all genes which did not fit in any other module, and which did not really share a common expression pattern, and hence this module is less likely to be of biological interest. If the network that has been constructed is a signed co-expression network - and hence the expression of any genes in the same module should be positively correlated with each other - my worry is that M1 might be such a module (and thus of more limited biological interest).

<i>page 12, "These genes represent a molecular network that can be targeted for therapeutic rescue of synaptic and cognitive function."

This is a strong statement, and I think that I would wish to see more mechanistic and causal studies beyond the bioinformatic analyses performed, in order to support. I would suggest that this statement might be made less emphatic.

A:
We have reworded the text as suggested.</i>

My apologies if I am misreading, but this sentence appears to be unchanged (p 12, lines 43-44)? I would suggest perhaps changing "can" to "could" or "might".

<i>Figure 3A. As previously stated, it would be good to indicate what proportion or number of the genes in each module are annotated with the indicated GO terms. It would also to be good to include in the Supplemental Material tables analogous to Sup. Table 3, giving the GO analysis for the other modules (even perhaps just the top 5 or 10 terms for each).

A:
In Table 1, we have provided information for each module, including functional description of each module, the top genes in each module and the number of genes in each module.</i>

I still feel that a potential misunderstanding by the reader could be avoided here, by including somewhere an indication of how many (or which) genes in each module are annotated with the term that has been used to describe that module's molecular function. While this information is available for Module 1 (by corrrelating with Supplemental Table 5), it is not, as far as I can telll, available for the other modules. Two ways of addressing this would be (i) to include a column in Table 1 indicating the number of genes in the module annotated with the term denoted in the "Molecular Function" column, or (ii) by including a sheet in the Supplementary spreadsheet, analogous to Supplemental Table 5, giving the top few terms in the GO analysis for every module.

——

I also noticed a couple of typos in the new revision:

p 4, line 38: Metascpae -> Metascape

p 9, line 13: reveled -> revealed
p 21, line 40: SNPA25 -> SNAP25

Editor Comments:
The tile: "Transcriptomic Analysis of Human Brains with Alzheimer's Disease Reveals the Key Determinant for Cognitive Deficits" goes beyond the what is demonstrated in the paper (i.e. the key determinant for cognitive deficits in Alzheimer's has not necessarily been revealed). Please revise the title to more accurately reflect your results.
