# Supplementary material for: Transcriptomic analysis of human brains with Alzheimer’s disease reveals the altered expression of synaptic genes linked to cognitive deficits
Source: Brain Commun. 2021 Jun 3;3(3):fcab123. doi: 10.1093/braincomms/fcab123 (PMC8374979; doi:10.1093/braincomms/fcab123)
Supplement: fcab123_Supplementary_Data [file Revision 1-Authors Response.pdf › Revision 1-Authors Response.pdf]

Reviewer(s)' Comments to Author:

Reviewer: 1

Comments to the Author
I thank the authors for their diligent response to my review comments, a good number of which were adopted. I still have some remaining issues, the most substantial concerning the biological follow ups in Figure 6. These are attached in a separate file.

4) I'm not used to seeing heatmaps like those in Figure 2A and C. I think I find the lack of clustering jarring – why was the decision made to not use clustering, at least for the rows? I am unclear as to exactly how the data are scaled – are they scaled and centered, or is some kind of ranking being used? Or is there no scaling? Please make this clearer. It would also be interesting to see how well AD clusters from Normal, rather than separating them as has been done here.

I disagree that row clustering best shows the expression differences in key synaptic genes between AD versus healthy control. If these genes are the ones that are most differentially expressed, they should be able to do a reasonable job of clustering the samples. This unbiased approach is much more convincing than column ordering according to disease status. My comment about scaling and centering was also not responded to – if you are not getting good clustering of the samples, it may be because these values are not scaled and centered – which means general expression patterns may be driving the clustering more than the differential abundance. I would like to see a scaled and centered plot with columns that are hierarchically clustered, I think there's a good chance this is much more persuasive than the current plot.
A:
Figure 2A and **2C** have been updated to show a scaled and centered plot with columns that are hierarchically clustered. The gene expression data used for the heatmaps in panels A and C was first log2 normalized then Z-scored. These data were then represented as a matrix, followed by selection of key pre- and post- synaptic markers. The data has been both row and column clustered. Row clustering was carried out via k-means sorting algorithm, and hierarchal clustering carried out via Euclidean distance, applied to column (sample) data, demonstrating the relationship between patient samples.

5) The in-house confirmation experiments in Figure 6 are poorly described and need more details. a. What are the demographics of the samples? Were they well matched for age, which is another determinant of synapse density? b. What method of microscopy was used for the imaging? What antibodies? c. What do the error bars on 6A show? SEM? d. All lanes of the western blots should be shown, if there are only 11 samples total e. The imaging focuses on the cell bodies of the neurons, and NeuN is primarily a nuclear marker for neurons. See Figure 1E and 1F here for someone who has attempted something similar using HRP methods:

1) The demographic details for Figure 6 have not been added to the supplementary Figure 2, there is only a western blot
A:
We have added a table with demographic information (see new **Sup. Table 8**).

2) The uncropped western blot is very disappointing. It highlights both the difficulty of using a single protein marker of a loading control, and the issues with only showing two columns of a multi column blot in a main figure. The Actin loading control varies by a

very large amount. Loading controls can be accurately used to correct for small differences between loading, but these differences are very large, with the densest bands getting very close to the non-linear range. This blot needs repeating with better, even loading, and ideally, a total protein stain as a loading control. I doubt that there is something funny going on specifically with Actin here, but it is possible.

A:

The Western blots are protein levels at the synaptic fraction of PFC from human samples, not proteins in the total lysates. Human samples usually have big variations. We compared the raw WB signal of actin in the synaptic fraction of PFC from control vs. AD samples, and found no significant differences (raw WB intensity normalized to control group, control: 1.0±0.17, n=5, AD: 1.05±0.12, n=6, p=0.81, two-tailed unpaired t-test with unequal variances), so we used actin as the loading control. We quantified the level of proteins normalized to the actin loading control in each sample, and some proteins show significant reduction in AD humans (e.g. SNAP-25, STX1A, PSD-95), while others did not reach the significance (e.g. VAMP2). None of these bands are in the non-linear range detected by the machine. The reduction of synapses and many synaptic proteins in AD humans makes us unable to use the total protein stain of synaptic fraction as the loading control.

3) I think my point was missed about using NeuN as a co-stain. NeuN labels the nuclei of neurons, not the synapses. The majority of the signal you are measuring therefore in these image fields is coming from the cell body or the nuclei, not the synapse. Given your entire paper is about the importance of the synapse, you actually need to be looking at synapses. You have identified synaptic markers that don't change with AD – why not co-stain with one of those, and look at a decrease in co-localization of signal with the marker that doesn't change?

A:

We have revised the immunostaining data (see new **Fig. 6C**) using a lower magnification (40x) to allow the better demonstration of synaptic protein-labeled puncta at all synapses, including those on spines, dendritic shafts and cell bodies. NeuN is used to show where the neurons are. We have found the significant reduction of STX1A and SNAP25 puncta, but not VMAP2 puncta, in AD samples, suggesting that the approach is sensitive enough to detect specific changes. We have also done the co-staining of STX1A or SNAP25 with another synaptic marker NR2B, and found that it is hard to tell individual neurons, so we have kept the NeuN data in Fig. 6C.

5) The process of Figure 3 currently seems a bit circular. I'm not familiar with the webCEMiTool, but it looks like it probably identified covarying genes/proteins, places them into modules, then look for over-representation of GO terms in these modules – this is your Figure 3A. So then when you take proteins from M1 and do your own GO analysis to find the same GO category at the top, this seems a little circular. Perhaps you could make it a little clearer what Figure 3D adds to the analysis?

This rebuttal is a clearer explanation of the thought process for this figure, but it still doesn't come through in the paper. I think this also calls back to Reviewer 2 mentioning that although SNARE proteins are the most enriched protein in Module 1, the number of proteins in this category is still low. This could be mentioned, and then used as a reason for looking at what other categories were also enriched in this module (something that WGCNA doesn't do out of the box

A:

Figure 3D-3F have been updated to more clearly represent a logical progression of our module analysis. Now Fig. 3A shows an overview of modular functional groups; Fig. 3B shows relative expression of each module in AD vs. control; Fig. 3C shows the distribution of Module 1 DEGs

(up- and down- regulated in AD); Fig. 3D shows a network displaying the relationship and interaction between Module 1 DEGs from top GO functional groups; Fig. 3E shows top GO pathways from Module 1 downregulated DEGs; Fig. 3F shows top GO pathways from Module 1 upregulated DEGs. We have updated Results and Figure Legends accordingly.

Reviewer: 2

Comments to the Author
My thanks to the authors for the changes that they have made to the manuscript in response to both reviewers' comments. I still have a few concerns (mostly related to the gene co-expression network analysis), which I think should be relatively easy to address.

<i>page 8, "With synaptic signaling being the most significantly downregulated GO category, we further explored dysregulated synaptic pathways in AD. .... (Figure 1C and 1D). ... (Figure 1C, -Log10 p-value = 14.37)."

It would be good to state what background gene set was used for the synaptic enrichment analysis (e.g. brain expressed genes vs only synaptic genes) as this makes a difference to interpretation of the enrichment p-values obtained.

My apologies if I have missed it, or have been unclear in my previous comment, but I would still appreciate a statement of the background gene set used in the SynGo enrichment analysis. My assumption would be that the whole set of brain expressed genes has been used as the background, as this is the default on the SynGo website (the alternative would be a custom gene list supplied by the user).
A:
We have added a statement that "brain expressed" background gene set was used in the SynGo enrichment analysis.

<i>page 8, "Hub co-expression analyses were performed on the 41 downregulated synaptic vesicle- clustered genes (Figure 1E), ... with SNAP-25 as the top-ranking node."

It would be great to state more explicitly, particularly for the benefit of non-bioinformatician readers, what hub co-expression analysis constitutes. I think from the Methods section, that this involves using the STRING database to construct an interaction network of the 41 down-regulated synaptic vesicle- clustered genes, and then using MCC to identify hub genes in this network? But I'm not clear what role co-expression plays in this?

Many thanks for the extra explanation of hub gene analysis. I am still unsure about the use of the word "co-expression" in the sentence following the new text, i.e. "Hub co-expression analyses also revealed…". Co-expression would imply to me that genes are linked in the network because they share similar expression patterns across samples; however, I believe that genes are linked in the network because their protein products are known or predicted to interact in the STRING database. It is certainly possible that I have misunderstood, but if not, should the word "co-expression" be removed here?
A:
We agree that "co-expression" is not a very clear term, so we have removed it.

I think that it would also be great to expand the legend for Figure 1E to explain that the top 50 hub genes are being shown (as was detailed by the authors in a response to Reviewer 1).
A:

We have expanded the figure legend to describe that the top 50 hub genes are being shown.

<i>page 9, "Among the M1 DEGs with fold-change of at least 10%, 198 were downregulated and 100 were upregulated."

I am a bit confused here. M1 is a module of 522 genes which share a common expression pattern across samples. Therefore I would have expected genes in M1 to show a similar pattern of expression in AD samples as compared to control samples; however some genes in M1 are strongly up-regulated in AD vs control, and some are strongly down-regulated in AD vs control. I may well be misunderstanding but how can, for example, GFAP and SYN2 share a common-expression pattern across samples, when GFAP is higher in AD vs control and SYN2 is lower in AD vs control? It may be worth including, in the Supplemental Material, plots of gene expression across all samples (for M1 at least), highlighting the expression pattern of the eigengene, and a few example genes such as GFAP and SYN2, to show how their expression pattern is related.

I apologise for any lack of clarity in my previous comments: I still have a concern remaining that, for example, GFAP and SYN2 (which are regulated in <i>opposite</i> directions in AD vs control) are contained within the same co-expression module.

Further to their answer could the authors possibly confirm whether they have constructed a <i>signed</i> or an <i>unsigned</i> co-expression network (unfortunately I am not familiar with webCEMiTool, and hence do not know which type of network it is constructing). If their gene co-expression network is indeed unsigned, then that would alleviate my concern about GFAP and SYN2 being contained within the same module (because anti-correlation of expression across samples would then count as linkage in the network, and hence it would not necessarily be surprising to find these two genes in the same module).

However, if the network is a signed co-expression network, I would remain worried that GFAP and SYN2 (one of which is up-regulated, the other down-regulated) could be contained within the same module for the following reason. In my previous experience of using WGCNA (a similar tool) for constructing co-expression networks, I have sometimes found there to be a "default" module which contains all genes which did not fit in any other module, and which did not really share a common expression pattern, and hence this module is less likely to be of biological interest. If the network that has been constructed is a signed co-expression network - and hence the expression of any genes in the same module should be positively correlated with each other - my worry is that M1 might be such a module (and thus of more limited biological interest).
A:
After a thorough look into the webCEMiTool and its associated manuscript (Sepúlveda-Hermosilla, G., et al (2019). webCEMiTool: Co-expression Modular Analysis Made Easy.), we noticed that the creators of the tool don't explicitly describe whether the module analysis is signed or unsigned, however, based on the information gathered (including from other groups who've published with this tool), we deduce that these tools utilized unsigned network analysis. This would explain why modules produced by webCEMiTool can contain genes that are up-regulated and genes that are down-regulated in the same module.

<i>page 12, "These genes represent a molecular network that can be targeted for therapeutic rescue of synaptic and cognitive function."

This is a strong statement, and I think that I would wish to see more mechanistic and causal studies beyond the bioinformatic analyses performed, in order to support. I would suggest that

this statement might be made less emphatic.

My apologies if I am misreading, but this sentence appears to be unchanged (p 12, lines 43-44)? I would suggest perhaps changing "can" to "could" or "might".
A:
We have changed “can” to “could” to make the statement less emphatic.

<i>Figure 3A. As previously stated, it would be good to indicate what proportion or number of the genes in each module are annotated with the indicated GO terms. It would also to be good to include in the Supplemental Material tables analogous to Sup. Table 3, giving the GO analysis for the other modules (even perhaps just the top 5 or 10 terms for each).

I still feel that a potential misunderstanding by the reader could be avoided here, by including somewhere an indication of how many (or which) genes in each module are annotated with the term that has been used to describe that module’s molecular function. While this information is available for Module 1 (by corrrelating with Supplemental Table 5), it is not, as far as I can telll, available for the other modules. Two ways of addressing this would be (i) to include a column in Table 1 indicating the number of genes in the module annotated with the term denoted in the "Molecular Function" column, or (ii) by including a sheet in the Supplementary spreadsheet, analogous to Supplemental Table 5, giving the top few terms in the GO analysis for every module.
A:
In addition to GO analysis of Module 1 (see revised **Sup. Table 5**), we have added a supplemental table that highlights the top functional groups and associated genes in Module 2-11 (see new **Sup. Table 6**).
——

I also noticed a couple of typos in the new revision:

p 4, line 38: Metascpae -> Metascape
p 9, line 13: reveled -> revealed
p 21, line 40: SNPA25 -> SNAP25
A:
All are corrected.

Editor Comments:
The tile: "Transcriptomic Analysis of Human Brains with Alzheimer’s Disease Reveals the Key Determinant for Cognitive Deficits" goes beyond the what is demonstrated in the paper (i.e. the key determinant for cognitive deficits in Alzheimer's has not necessarily been revealed). Please revise the title to more accurately reflect your results.
A:
We have changed the title to "Transcriptomic Analysis of Human Brains with Alzheimer’s Disease Reveals the Altered Expression of Synaptic Genes Linked to Cognitive Deficits"
